# A universal method for automated gene mapping

**DOI:** 10.1186/gb-2005-6-2-r19

**Published:** 2005-01-17

**Authors:** Peder Zipperlen, Knud Nairz, Ivo Rimann, Konrad Basler, Ernst Hafen, Michael Hengartner, Alex Hajnal

**Affiliations:** 1 Institute of Molecular Biology, University of Zürich, Winterthurerstrasse 190, CH-8057 Zürich, Switzerland; 2 Institute of Zoology, University of Zürich, Winterthurerstrasse 190, CH-8057 Zürich, Switzerland

## Abstract

Small insertions or deletions (InDels) constitute a ubiquituous class of sequence polymorphisms found in eukaryotic genomes. Here, we present an automated high-throughput genotyping method that relies on the detection of fragment-length polymorphisms (FLPs) caused by InDels. The protocol utilizes standard sequencers and genotyping software. We have established genome-wide FLP maps for both *Caenorhabditis elegans *and *Drosophila melanogaster *that facilitate genetic mapping with a minimum of manual input and at comparatively low cost.

## Abstract 2

A high-throughput method for genotyping by mapping InDels. This method has been used to create fragment-length polymorphism maps for *Drosophila* and *C. elegans*.

## Background

For humans and model organisms, such as worms and flies, the availability of high-density sequence polymorphism maps greatly facilitates the rapid mapping and cloning of genes [1-3]. Key advantages of most molecular polymorphisms are the fact that they are codominant and in general phenotypically neutral. The vast majority of sequence polymorphisms are single-nucleotide polymorphisms (SNPs).

The most direct approach for SNP detection is sequencing of a PCR product spanning the polymorphism, but this is too costly and labor intense for high-throughput genotyping. For this reason, several different strategies and methods have been developed in order to detect SNPs more efficiently. In general, assays can be grouped into strategies, where the nature of the SNP is determined by directly analyzing the primary PCR product and those that require a secondary assay performed on the primary amplification product [4-6]. An important strategy of the first group is the 5' nuclease assay, where allele-specific, dual-labeled fluorescent TaqMan probes guarantee specificity [7]. However, the need for two dual-labeled fluorescent probes, expensive specialized chemistry and specialized machinery increase the costs per assay of this approach significantly. Similarly, denaturing high-performance liquid chromatography (DHPLC) also analyses the primary amplification product [8]. This approach is based on melting differences of homo- versus heteroduplex DNA fragments under increasingly denaturing conditions and requires no specific labeling of the PCR fragments. However, conditions have to be optimized for every assay, throughput is limited and specialized equipment is required. DHPLC has been used in small-scale genotyping projects in *Drosophila melanogaster *[9].

Of the methods that detect the SNP in a secondary assay, restriction fragment length polymorphism (RFLP) analysis are very popular [10]. For this purpose, only those SNPs that alter a restriction site are analyzed. A great advantage of RFLP analysis is that no specialized equipment is needed and it can be carried out in every laboratory. RFLP maps recently established for *Caenorhabditis elegans *and *Drosophila *are used regularly in genotyping projects [2,3,11]. However, RFLP analysis requires significant manual input. Moreover, the use of different restriction enzymes with different reaction requirements adds another level of complexity that makes this method difficult to automate. Primer-extension-based technologies have also gained some prominence [12]. Here, a primer that anneals right next to the polymorphism is extended by one polymorphism-specific terminator nucleotide. Extension products are analyzed by size or, alternatively, by differences in the behavior of incorporated versus non-incorporated terminator nucleotides under polarized fluorescent light [13]. Swan and colleagues [14] have developed a set of fluorescence polarization-template directed incorporation (FP-TDI) assays for *C. elegans*. However, this approach is labor intensive and requires specialized chemistry and equipment. Using DNA microarrays, large numbers of SNPs can be analyzed in parallel, but the number of individuals that can be analyzed is low because of the high cost per chip [15,16].

Besides SNPs, short tandem repeats (STRs) or microsatellites represent another class of sequence polymorphisms used for genotyping [17-21]. STRs result in fragment length differences that are either detected on gel-based or capillary sequencers or high-resolution hydrogels (Elchrom Scientific Inc.). One advantage of STRs over SNPs is that they are highly polymorphic and are thus ideal for measuring the degree of variability in natural populations. STRs are, however, present at much lower density than SNPs and are therefore not suitable for high-resolution mapping of genes.

Interestingly, a significant proportion of the currently available polymorphisms are caused by small insertions or deletions (InDels). Weber *et al*. [22] identified a genome-wide set of about 2,000 human InDel polymorphisms and estimated that InDels comprise at least 8% and up to 20% of all human polymorphisms. This is in line with the findings of Berger and co-workers [2] who found that 16.2% of polymorphisms in *Drosophila *are of the InDel type. Also, two independent studies in *C. elegans *found that InDels constitute between 25% and 28% of all polymorphisms [3,14]. In addition, those studies found that the vast majority of InDels are due to 1-2 base-pair (bp) differences (65% in *Drosophila *[2], 84% in *C. elegans *[3]).

To take full advantage of this class of small InDel polymorphisms, we have developed a strategy that allows us to detect most, if not all, InDels by analyzing the lengths of primary PCR products on a capillary sequencer at single base-pair resolution. We call these assays fragment length polymorphism (FLP) assays. Importantly, this approach can easily be automated on standard robotic pipetting platforms as it involves a simple PCR reaction setup. Furthermore, allele calling is performed automatically using the Applied Biosystems GeneMapper software commonly used for genotyping STRs (Materials and methods).

To demonstrate the feasibility of this strategy, we have validated 112 evenly spaced FLP assays at 3 centimorgan (cM) resolution in *C. elegans *(one every 0.9 megabase-pair (Mbp)) and 54 FLP assays at 4 cM resolution for the *Drosophila *autosomes. This set of FLP assays allows us to rapidly map mutations to small chromosomal subregions with a minimum of manual input. Furthermore, we provide a list of predicted InDels for which additional assays can be readily designed in the chromosomal subregion of interest. Those non-validated FLPs enhance the resolution of the map by a factor of 5.6 and 17.9, respectively.

We show the usefulness of this approach by identifying novel alleles of previously characterized genes. In summary, we have taken advantage of a publicly available dataset to adapt a technology widely used for STR analysis to genetic mapping. Thanks to the complete automation of genotyping, this approach is considerably faster, more reliable and cheaper than previously used mapping strategies in *C. elegans *or *Drosophila*.

## Results and discussion

### Detection of fragment length polymorphisms (FLPs)

To detect a FLP, the region of interest is amplified in a standard PCR reaction with one fluorescently labeled primer, and the PCR products are directly analyzed on a capillary sequencer. Fragment sizes are determined automatically relative to an internal size standard with AppliedBiosystem's GeneMapper software (for details see Materials and methods). The software then allocates fragment sizes to previously calibrated genotypes.

**Figure 1:**
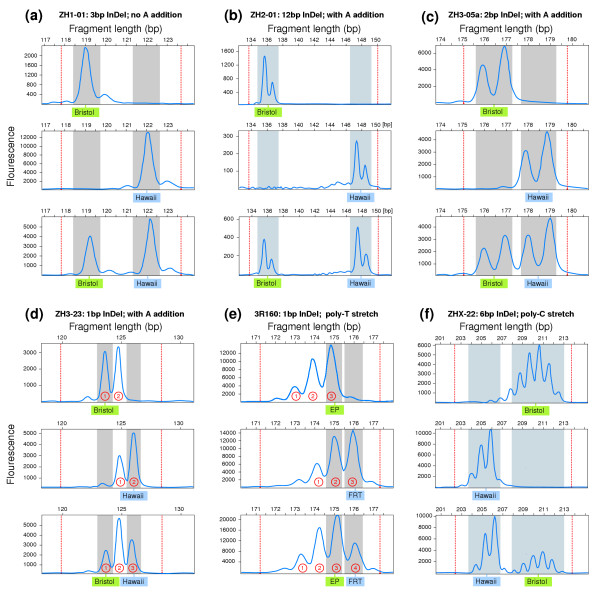
FLP detection of InDels of various sizes in homozygotes and heterozygotes. In each panel the top two graphs show the homozygotes and the bottom graph the heterozygote. Gray shaded areas mark the defined expected allele lengths and red lines indicate the borders of a predefined window of expected allele lengths. **(a-c) **Detection of InDels in *C. elegans *that show increasing levels of adenosine (A) addition. (a) 3-bp InDel *ZH1-01 *with no A addition; (b) 12-bp InDel *ZH2-01 *with A addition; (c) 2-bp InDel *ZH3-05a *with A addition. **(d) **1-bp InDel *ZH3-23 *in *C. elegans *with A addition. An unambiguous allele-call can be made, irrespectively of the level of A addition: both homozygous samples consist of two peaks at different positions, whereas the heterozygous animal exhibits three peaks. **(e) **The 1-bp InDel *3R160 *in *Drosophila *runs over a 12-13 nucleotide poly(T) stretch and exhibits stutter bands. Even in this case, a clear allele-call can be made (three peaks in homozygous and four peaks in heterozygous animals). **(f) **The 6-bp InDel *ZHX-22 *in *C. elegans *occurs in a poly(C) stretch and the FLP graph displays stutter bands. As expected, the longer fragment exhibits a higher degree of stuttering.

Taq polymerase has the tendency to catalyze the addition of adenosine (A) to the 3' end of PCR products. This activity could make it difficult to achieve the single base-pair resolution required to assay all available InDels and may hamper allele-calling [23]. However, we have found that the sensitivity of a capillary sequencer and the genotyping software is sufficient to allow for unambiguous allele assignment even for 'difficult' sequences exhibiting 3' A addition. The examples shown in Figure 1a-d illustrate that robust genotyping is feasible for 1-bp InDels even when 3' A addition occurs. Another problem is the stuttering of the polymerase when it encounters poly(N) stretches. However, larger InDels are reliably detected by the software in poly(N) stretches (Figure 1f), and in a few difficult cases visual inspection can even resolve and unambiguously assign 'stuttering' 1-bp InDels according to the location and number of peaks (Figure 1e).

Genotyping with FLP assays is extremely accurate. In a control experiment, we genotyped all 96 samples of the fly strains FRT42B and EP0755 for the 1-bp InDel *2R090 *and 231 samples homo- and heterozygous for the *C. elegans *Bristol and Hawaii backgrounds, respectively, for the 1-bp InDel *ZH5-16. 2R090 *exhibits both stuttering and A addition and hence is especially difficult to resolve (see Additional data file 8). The genotype was correctly and automatically assigned by GeneMapper in all 423 assays. Thus, automated genotyping based on FLPs is sensitive down to single base-pair resolution and is extremely robust. The accuracy of FLP mapping is comparable to other methods such as TaqMan (error rate less than 1 in 2,000 [24]), minisequencing (99.5% [25]), and pyrosequencing (97.3 % [25]).

### *C. elegans *and *Drosophila *FLP maps

**Figure 2:**
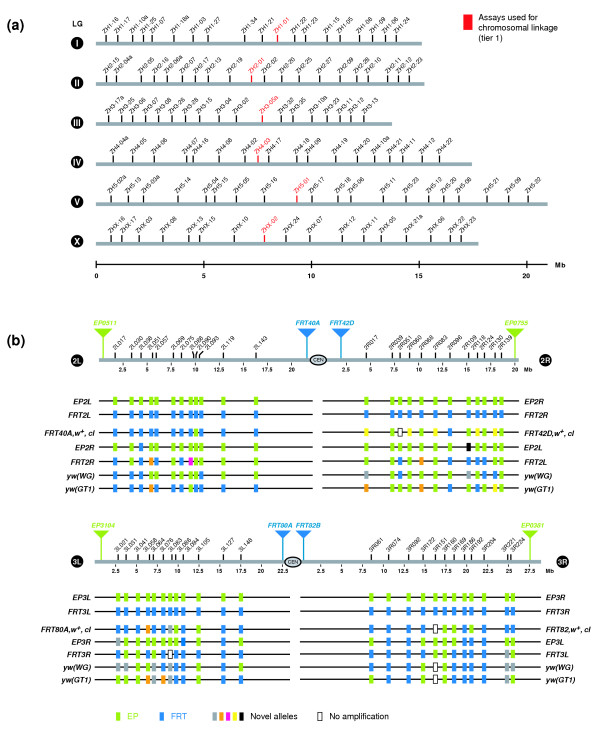
*C. elegans *and *Drosophila *FLP maps. **(a) **The *C. elegans *FLP map. Marker names comprise a *ZH *prefix followed by the chromosome number and a unique identifier number. Markers used in first-level assays (Tier 1) for determination of chromosomal linkage are in red, those used for second-level assays (Tier 2) for higher resolution mapping are in black. **(b) **The *Drosophila *FLP map of chromosomes 2 and 3. The FRT sites and EP elements are symbolized by blue and green triangles, respectively. The strains that were genotyped are shown below each chromosome. Green indicates the EP genotype, blue the FRT genotypes and new alleles are shown in other colors.

In *C. elegans*, genetic experiments are performed almost exclusively in the background of the standard wild-type strain N2 (*C. elegans *variety Bristol) [26]. For gene mapping experiments, the polymorphic strain CB4856 (*C. elegans*, variety Hawaii) has proved extremely useful [3]. When compared to N2, CB4856 contains on average one SNP every 840 bp and approximately 25% of all polymorphisms are InDels [14]. Starting from the dataset previously published by Wicks *et al. *[3], 112 FLPs that are evenly spaced on the physical map of *C. elegans *were validated to date (Figure 2a). The confirmation rate of the predicted InDels was 88% (*n *= 169). Most failures to detect a FLP are probably due to original sequencing errors. The average distance between neighboring FLP assays is about 0.9 Mbp. This physical distance corresponds to about 3 cM, assuming 300 kb per map unit, and encompasses between 100 and a maximum of 500 genes (Figure 2a). The length of the amplicons ranges from 100 to 444 bp, and the fragment length differences are between 1 and 21 bp (Additional data file 9). If necessary, another 2,454 predicted InDels are available to increase the mapping resolution down to 50 kbp on average (Additional data files 12-17).

To establish a *Drosophila *FLP map, a set of 54 FLP assays (12 to 17 per arm of the two major autosomes) was validated from the list of polymorphisms identified by Berger *et al*. [2] (Figure 2b, and Additional data file 10); high-resolution X-chromosomal SNP and FLP maps have yet to be established. Similarly to *C. elegans*, the confirmation rate of the predicted *Drosophila *InDels was 80% (*n *= 30). Furthermore, another 509 InDels are predicted at 248 sites for which an assay can be established to discriminate between EP and FRT strains (Additional data file 18). The validated *Drosophila *FLP assays were evenly spaced on the genetic map with an average distance between neighboring assays of about 4 cM, corresponding to an average resolution of 1.77 Mbp on the physical map encompassing 95,55 Mbp [27,28]. Taking into account the non-validated InDels, the maximal average resolution is currently 314 kb or 0.7 cM. On the left arm of chromosome 3, where the genetic map is inexact, FLPs were spaced on the physical map assuming colinearity between the two maps. The length of amplicons ranges from 99 to 365 bp, and the size difference ranges from 1 to 54 bp (Additional data file 9).

Our *Drosophila *FLP assays are in part derived from a set of InDels of size difference 7 bp or more (termed PLPs by Berger *et al*. [2]). However, since 86.8% of all *Drosophila *InDels exhibit a length difference of one to six nucleotides [2], so far only a small subset of the available InDels has been covered. The approach presented here significantly increases the number of possible FLP assays for genotyping and offers a greater flexibility and higher resolution.

### FLP mapping of *C. elegans *genes

**Figure 3:**
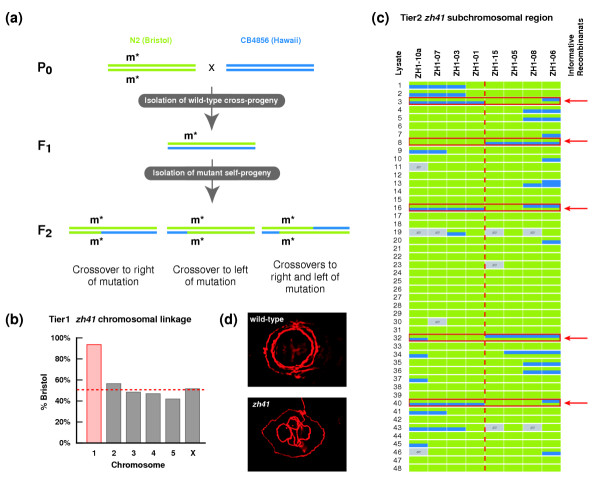
FLP mapping in *C. elegans*. **(a) **Crossing scheme used to map mutations generated in the N2 Bristol background. The different classes of recombinants recovered in the F_2 _generation are shown. **(b) **Analysis of the *zh41 *mutation with Tier 1 assays establishes linkage to chromosome I. **(c) **Analysis with Tier 2 places *zh41 *between assays *ZH1-01 *and *ZH1-15*. ND, no data as a result of PCR reaction failure. **(d) **Ventral views of the vulva in wild-type and *zh41 *L4 larvae stained with the adherens junction antibody MH27 [44]. In the wild type, the vulval cells have fused to generate the torroids that appear as concentric rings. *zh41 *mutants exhibit the same fusion defects observed in other *lin-11 *alleles [33].

To demonstrate the usefulness of the *C. elegans *FLP map, we mapped three previously characterized mutations on chromosome II that exhibit diverse phenotypes. Those were the centrally located *let-23(sy1) *allele that causes an 80% penetrant *vulvaless *phenotype [29], *rol-1(e91) *in the middle of the left chromosome arm, which causes the animals to roll around their body axis [30], and the *unc-52(e444) *mutation located at the right end of the chromosome, which results in a paralyzed phenotype [31]. Mutant hermaphrodites were crossed with CB4856 males, and wild-type F_1 _cross-progeny was selected (F_1 _self-progeny would exhibit a mutant phenotype). Finally, mutant self-progeny was isolated in the F_2 _generation and used for genotyping (Figure 3a). To minimize the number of PCR reactions, we pursued a two-step strategy. First, we determined chromosomal linkage by analyzing 16 individual F_2 _animals (corresponding to 32 chromosomes in total) with one centrally located FLP assay per chromosome (Tier 1, Figure 2a). This allowed us to establish clear linkage to chromosome 2 for all three mutations (Additional data file 2). Surprisingly, the *rol-1(e91) *mutation showed linkage to the X chromosome of N2 in addition to chromosome II. This pseudo-linkage could be due to a suppressor of the Rol phenotype present on the CB4856 X chromosome. In a second step, 48 F_2 _animals for each mutation were analyzed with eight FLP assays along chromosome 2 (Tier 2, Figure 2a). In this way, we could narrow down the three mutations to the correct chromosomal subregions (Additional data files 3-5). We used the same strategy to map the *zh41 *mutation that was identified in a forward genetic screen for mutants exhibiting a loss of *egl-17::gfp *expression in the vulval cell linage ([32] and I. Rimann and A. Hajnal, unpublished work). Analysis with Tier 1 established linkage to chromosome 1 (Figure 3b), and Tier 2 narrowed down the candidate region to an interval of 2.2 Mbp containing 498 genes (Figure 3c). The phenotype of *zh41 *animals is similar to the phenotype caused by loss-of-function mutations in *lin-11*, which maps to the same interval in the center of chromosome I [33]. Like *lin-11 *mutants, *zh41 *animals exhibit a penetrant protruding vulva (Pvl) phenotype, and staining of the adherens junctions with the MH27 antibody showed defects in the formation of the vulval torroid rings (Figure 3d) [33]. Subsequent sequencing of the *lin-11 *locus in *zh41 *animals revealed a point mutation that results in a change of leucine 274 to phenylalanine. Furthermore, *zh41 *failed to complement *lin-11(n389)*, indicating that the *zh41 *mutation in the *lin-11 *open reading frame (ORF) is responsible for the vulval phenotype.

In cases where a mutation maps to an interval that contains no obvious candidate gene, we first screen for additional informative recombinants by FLP analysis and then refine the map position by extracting more FLPs from our set of non-validated InDels (Additional data files 12-17) and by genotyping existing SNPs in the candidate interval [3]. In many cases, this resolution is sufficient to identify the affected gene through RNA interference (RNAi) analysis of the genes in the corresponding interval [34]. (See Additional data file 6 for a detailed flowchart of the mapping process).

In summary, FLP mapping in *C. elegans *allows us to rapidly map a mutation down to a small region containing, on average, 200 candidate genes by crossing a mutant strain to CB4856 and analyzing 48 F_2 _animals with 300 to 500 PCR reactions.

### Genotyping *Drosophila *strains with FLP assays

In contrast to the well defined genetic backgrounds used for *C. elegans*, zebrafish (*Danio rerio*) or *Arabidopsis *genetics, *Drosophila *strains are very heterogeneous and of ill-defined origin [2,9,11]. In this respect, gene mapping in *Drosophila *resembles human genetics in that standard inbred lines do not exist and the genotypes of the parental lines have to be determined first. As genome-wide polymorphism databases for reference strains are available [2,11], a line of interest can be crossed with two reference strains, such as EP and FRT (see below). Owing to the codominant character of sequence polymorphisms, at least one of the two respective crosses will distinguish between the mutant and the mapping chromosomes. To further facilitate mapping with our set of FLP assays, we genotyped several common laboratory lines such as two 'wild-type' *yw *strains for the whole set, four FRT-Minute or FRT-cell-lethal strains at the relevant autosomal arms [35], as well as the FRT and EP reference strains at both relevant autosomal arms (Figure 2b). Surprisingly, the FRT and EP lines are largely not of FRT or EP genotype on the chromosome arm for which they have not been calibrated. Overall, we found novel alleles for 18 of the 48 assays, and in an extreme case, we even observed five different alleles in five examined strains (*2R017*, Figure 2b). This result further highlights the heterogeneity of *Drosophila *strains (see Additional data file 1 for further details on FLP calibration and fly genetics).

### FLP mapping in *Drosophila*

**Figure 4:**
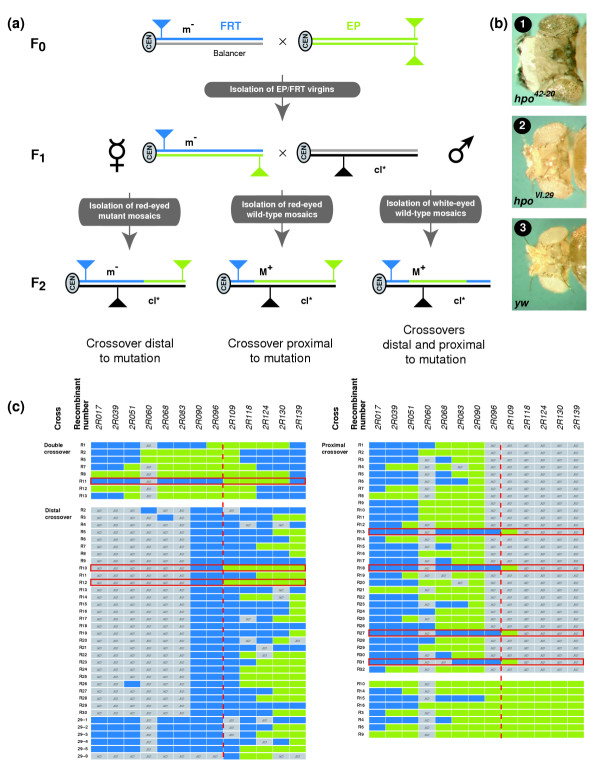
FLP mapping in *Drosophila*. **(a) **Crossing scheme used to map mutations generated in the FRT background and recombined with an EP line. The different classes of recombinants recovered in the F_2 _generation are shown. **(b) **Big head phenotypes of the *hippo *null allele *hpo*^42-20 ^(1) and the *VI.29 *mutation (2). A wild-type control is shown in (3). **(c) **FLP mapping of the *VI.29 *mutation on chromosome 2R. Analysis of the different classes of recombinants places the mutation between markers *2R096 *and *2R109 *(dashed red line). Informative recombinants are boxed in red. ND, not determined or no data as a result of PCR reaction failure.

In a genetic screen devised to isolate genes that regulate growth and are situated on chromosome 2R, we found a complementation group characterized by a mild overgrowth phenotype (Figure 4b (2), and C. Rottig and E.H., unpublished work). From a cross between allele *VI.29 *and *EP0755 *we recovered three types of recombinant chromosomes: recombinants with a crossover proximal or distal to the mutation, respectively, and double-crossovers (Figure 4a, see also Additional data file 1 for further details on the crossing scheme). The mutation could be placed 16.9 cM proximal to EP0755 and 38.7 cM distal to FRT42D. The FLPs in the recombinant flies were directly analyzed without backcrossing the recombinant chromosome into a parental strain background. DNA was prepared from recombinants by a novel high-throughput protocol (see Materials and methods). We genotyped 34 distal crossover events, 40 proximal crossovers, and eight double-crossovers. This analysis placed the mutation between markers *2R096 *and *2R109 *(Figure 4c). This interval includes the tumor suppressor *hippo *[36], and subsequent complementation analysis confirmed *VI.29 *as a weak *hippo *allele (data not shown). Furthermore, data from this and other FLP mappings in this region allowed us to further refine the genetic map (Additional data file 11). This kind of experimental data is helpful to space new FLP assays more evenly on the genetic map should the available map turn out to be inexact.

If the resolution of the validated FLP map is too low to identify a candidate gene, we further refine the map position by several approaches. First, we design novel FLP-assays in the region of interest and genotype the most informative recombinants from the first round of FLP mapping (Additional data file 18). Second, we genotype recombinants with SNPs available in the region of interest and resolve them by RFLP, sequencing or DHPLC [2,9]. Third, we perform complementation analysis with recently established *Drosophila *lines with molecularly defined deletions [37,38]. (See Additional data file 7 for a detailed flowchart illustrating the mapping process.)

## Conclusions

We have developed an automated method to detect most naturally occurring InDel polymorphisms at single base-pair resolution. Since a significant fraction of polymorphisms are caused by InDels of only a few base pairs (for example, 8% to 20% in humans [22]) the resolution of the medium-density FLP maps can be greatly increased where necessary, for example during the positional cloning of genes. We are therefore continually designing new FLP assays according to our specific needs using the predicted FLPs (Additional data files 12-18). The full automation of the genotyping has three main advantages when compared to manual methods. First, the error rate (the number of wrongly assigned genotypes) is extremely low, as it was not measurable in 432 assays. Second, genotyping can be done very rapidly and at a high-throughput with little manpower. The automatic allele-calling, in particular, saves much time. As the identification of informative recombinants is usually the rate-limiting step, FLP mapping is very helpful in extracting the few relevant recombinants from a large number of samples. Third, thanks to the standardized conditions, the low error rate and the absence of a secondary assay, FLP mapping is considerably cheaper than the previously published 'manual' mapping methods [2,3]. Unlike other high-throughput methods like TaqMan, Pyrosequencing, DHPLC, fluorescence polarization or primer-extension assays, FLP mapping does not require any investment in specialized equipment. It can be done in any molecular biology lab with access to a sequencing facility equipped with a capillary- or gel-based system, which usually includes the genotyping software. PCR costs are marginally higher because of the use of fluorescently labeled primers, but there are no added expenses for secondary enzymatic assays.

It seems likely that in most organisms the frequency of polymorphisms caused by InDels is in the same range as found in humans, *C. elegans *or *Drosophila*. For example, 7.3% of the *Arabidopsis *sequence polymorphisms are InDels [39]. Thus, FLP mapping can easily be adapted to any organism for which polymorphism maps have been established, as there is no conceptual difference between human, *Arabidopsis*, *C. elegans *or *Drosophila *FLPs.

## Materials and methods

### *C. elegans *and *Drosophila *culture techniques and alleles

Culturing and crossing of *C. elegans *was done according to standard procedures described in [26]. *C. elegans *alleles used were: LG I: *lin-11(zh41), lin-11(n389)*; LG II: *rol-1(e91)*, *let-23(sy1)*, *unc-52(e444)*. *Drosophila *strains and the genetic screen have been described previously [9,35,40-42].

### Single worm DNA extraction

Adult worms were collected in 10 μl lysis buffer (50 mM KCl, 10 mM Tris pH 8.2, 2.5 mM MgCl_2_, 0.45% NP-40, 0.45% Tween-20, 100 μg/ml freshly added proteinase K) and incubated for 60 min at 65°C followed by heat-inactivation of proteinase K at 95°C for 10 min. Before PCR, 90 μl double-distilled H_2_O (ddH_2_O) was added to obtain a total volume of 100 μl per lysate.

### Fly DNA extraction

DNA from recombinant flies was extracted in bulk by squishing flies through mechanical force in a vibration mill (Retsch MM30) programmed to shake for 20 sec at 20 strokes per second [43]. Single flies were placed into wells of a 96-well format deep-well plate with each well filled with 200 μl squishing buffer (10 mM Tris-Cl pH 8.2, 1 mM EDTA, 0.2% Triton X-100, 25 mM NaCl, 200 μg/ml freshly added proteinase K) and a tungsten carbide bead (Qiagen). The deep-well plate was then sealed with a rubber mat (Eppendorf) and clamped into the vibration mill. (Tungsten carbide beads can be recycled: after an overnight incubation in 0.1 M HCl and thorough washing in ddH_2_O the beads are virtually free of contaminating DNA.) Debris was allowed to settle for about 5 min, and 50 μl of each supernatant were transferred into a 96-well PCR plate. The reactions were incubated in a thermo-cycler for 30 min at 37°C and finally for 10 min at 95°C to heat-inactivate proteinase K. Before PCR amplification, the crude DNA extracts were diluted 20-fold to reduce the concentration of proteins that might be harmful for the capillary sequencer.

### PCR and FLP fragment analysis

Diluted single-worm lysates (2 μl samples) or single fly extracts were added to 23 μl PCR reaction mix. Final concentrations in the PCR reaction were: 0.4 μM forward/reverse primer, 0.2 mM dNTPs, 2 mM MgCl_2_, 1x PCR reaction buffer, 0.25 U EuroTaq polymerase (Euroclone). PCR reaction setup was done in 96-well plates using a Tecan Genesis pipetting robot with disposable tips. PCR was carried out in two MJR thermo-cyclers that are integrated into the robot. The current setup allows for the sequential processing of six 96-well plates at a time. Cycling parameters were 2 min 95°C, 20 sec 95°C, 20 sec 61°C (-0.5°C for each cycle), 45 sec 72°C (for 10 cycles) followed by 24 cycles of 20 sec 95°C, 20 sec 56°C, 45 sec 72°C and a 10 min 72°C final extension. Following PCR, reactions were diluted 1:100 in water, and 2 μl diluted PCR products were mixed with 10 μl HiDi formamide containing 0.025 μl LIZ500 size standard (Applied Biosystems). This dilution before analysis on the capillary sequencer is necessary to reduce signal intensity because too strong signals compromise data analysis. In addition, sample dilution reduces the risk of damaging the capillaries with proteins or lipids present in the crude lysates. The dilution was done with standard tips using the Tecan Genesis pipetting station. Carryover of fragments was prohibited by a simple wash step with H_2_O. Fragments were analyzed on an ABI3730 capillary sequencer using POP7 polymer according to standard procedures. Data were analyzed using AppliedBiosystems GeneMapper software and raw data were treated further with Microsoft Excel.

## Additional data files

The following additional data are available with the online version of this article. Additional data file 1 contains general information on fly genetics.

Further *C. elegans *mapping results are given in Additional data files 2,3,4 and 5. Detailed flowcharts illustrating the FLP mapping process are shown in Additional data files 6 and 7. Additional data file 8 contains electropherograms demonstrating the accuracy of allele-calling. Additional data files 9 and 10 contain tables of primer and sequence data of experimentally verified FLP assays in *C. elegans *and *Drosophila*, respectively. Additional data file 11 contains a table of the refined genetic distances for FLP assays on the right arm of *Drosophila *chromosome 2. Additional non-validated FLPs can be found in Additional data files 12,13,14,15,16 and 17 (*C. elegans*) and Additional data file 18 (*Drosophila*).

## Supplementary Material

Additional data file 1 General information on fly genetics Click here for additional data file

Additional data file 2 Proof-of-principle for chromosomal linkage with 3 known mutations on chromosome 2. Assays used to assess linkage were ZH1-01, ZH2-01, ZH3-05a, ZH4-03, ZH5-01 and ZHX-02 Click here for additional data file

Additional data file 3 Mapping of *let-23* to its subchromosomal region (*C. elegans*) Click here for additional data file

Additional data file 4 Mapping of *rol-1* to its subchromosomal region (*C. elegans*) Click here for additional data file

Additional data file 5 Mapping of *unc-52* to its subchromosomal region (*C. elegans*) Click here for additional data file

Additional data file 6 *C. elegans* FLP mapping flow chart Click here for additional data file

Additional data file 7 *Drosophila* FLP mapping flow chart Click here for additional data file

Additional data file 8 Electropherograms demonstrating the accuracy of allele-calling Click here for additional data file

Additional data file 9 Tables of primer and sequence data of experimentally verified FLP assays in *C. elegans* Click here for additional data file

Additional data file 10 Tables of primer and sequence data of experimentally verified FLP assays in *Drosophila* Click here for additional data file

Additional data file 11 A table of the refined genetic distances for FLP assays on the right arm of *Drosophila *chromosome 2 Click here for additional data file

Additional data file 12 Additional non-validated FLPs (predicted *C. elegans* InDels LGI) Click here for additional data file

Additional data file 13 Additional non-validated FLPs (predicted *C. elegans* InDels LGII) Click here for additional data file

Additional data file 14 Additional non-validated FLPs (predicted *C. elegans* InDels LGIII) Click here for additional data file

Additional data file 15 Additional non-validated FLPs (predicted *C. elegans* InDels LGIV) Click here for additional data file

Additional data file 16 Additional non-validated FLPs (predicted *C. elegans* InDels LGV) Click here for additional data file

Additional data file 17 Additional non-validated FLPs (predicted *C. elegans* InDels LGX) Click here for additional data file

Additional data file 18 Additional non-validated FLPs (*Drosophila*) Click here for additional data file
