# Supplementary material for: A universal method for automated gene mapping
Source: Genome Biol. 2005 Jan 17;6(2):R19. doi: 10.1186/gb-2005-6-2-r19 (PMC551539; doi:10.1186/gb-2005-6-2-r19)
Supplement: Additional data file 1 — General information on fly genetics [file gb-2005-6-2-r19-s1.doc]

# Supplementary information

### Drosophila genetics

Berger et al. [2] have chosen four sets of paired strains to genotype the major autosomal arms. These pairs consisted of an *FRT* strain characterized by the presence of an *FRT* insertion in the vicinity of the centromere [36] (figure 1B) and a strain harboring phenotypically neutral integrations of the *white^+^* marked *EP* transposon close to the telomere [37]. *FRTs* are widely used in large-scale genetic mosaic screens designed to identify recessive lethal mutations in heterozygous animals [38]. The *EP* insertions at the tip of the autosomal arms have been selected in order to facilitate recovery of recombinants over almost the entire arm. SNP and FLP mapping of mutations from clonal *FRT* screens is therefore most easily accomplished by resorting to the four respective *EP* lines for which the polymorphisms have been calibrated. The set of polymorphisms is also useful to map mutations in other strain backgrounds, like in the two “wild-type” *yw* strains we have genotyped. However, as these strains are of mixed *EP, FRT,* and novel genotypes along the chromosomal arms, it may turn out to be insufficient to only set up one recombination. For example, on chromosome 2L the *yw (WG)* strain both carries *EP, FRT,* and novel information (figure 1B). *FRT* - *yw (WG)* recombinants will be uninformative at four out of 12 FLPs, while *EP* - *yw* recombinants will be informative at those four assays, but will be uninformative at seven FLPs. One FLP exhibiting a novel genotype will be informative to both *EP* and *FRT*. On the other hand, 11 out of 12 FLP assays on chromosome 2L will be informative in crosses of the strains *EP2L* and *yw (GT)* (figure 1B).

In order to aid mapping with the four pairs of *FRT* and *EP* strains on the entire autosomes, we genotyped them on the respective other arms. Interestingly, the *FRT* strains are not of *FRT* genotype on the arms for which they have not been calibrated. Similarly, the third chromosomal *EP* insertions are not genotyped as *EP* on the other arms (figure 1B). The second chromosomal *EP* lines are largely isogenic except for FLPs 2L038 and 2R109. The escaper 2R109 constitutes a novel allele and sequence analysis shows that it contains both *FRT* and *EP* information and thus may derive from a gene-conversion event (supplementary figure 5). We conclude that the *FRT* and *EP* lines were not isogenized prior to P-element mobilization, or, alternatively, have had the opportunity to recombine with the different genetic background brought in from the transposase containing line, or may also have received genetic information from the balancer chromosomes.

The *FRT* strains are most commonly used in mosaic screens in which mutant tissue is given a proliferative advantage. To permit clonal expansion the *FRT* chromosome is put in trans over a homologous chromosome (carrying an *FRT* site, a dominant *white^+^* marker, and a *Minute* or cell-lethal mutation, respectively [33]. For meiotic mapping, recombinant *EP-FRT* chromosomes are selected in the genetic context of the homologous chromosome. These recombinant flies then contain three different strain backgrounds: *FRT, EP*, and homologous chromosome. The recombinant chromosome can either be backcrossed into the parental backgrounds or the selected recombinants can be directly assessed if the genotype of the homologous chromosome is known. This approach saves one generation (figure 4A, [9]). Therefore, we genotyped the chromosomal arms of the homologous chromosomes carrying the *Minute* or cell-lethal markers (figure 1B). Knowledge of this genotype allows for an unequivocal assignment of the recombinant chromosome by inference. For example, if the homologous chromosome has been genotyped *“EP”* at a given FLP and the recombinant would only show *EP* information, then the recombinant chromosome must be *“EP”*, too.
