# Supplementary material for: A universal method for automated gene mapping
Source: Genome Biol. 2005 Jan 17;6(2):R19. doi: 10.1186/gb-2005-6-2-r19 (PMC551539; doi:10.1186/gb-2005-6-2-r19)

Supplementary Figure 1: Proof-of-principle for chromosomal linkage with 3 known mutations on chromosome 2. Assays used to assess linkage were ZH1-01, ZH2-01, ZH3-05a, ZH4-03, ZH5-01 and ZHX-02.

let-23

Genetic Position: 1.06

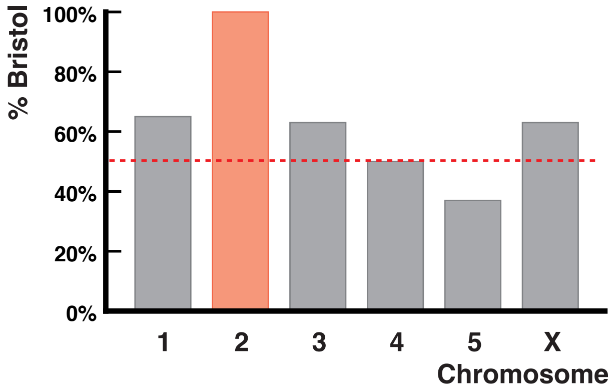

rol-1

Genetic Position: 6.99

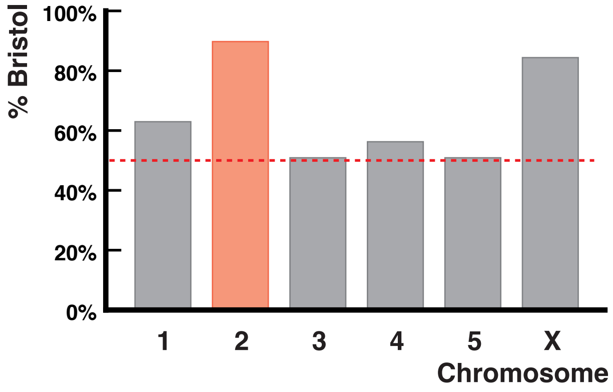

unc-52

Genetic Position: 23.31

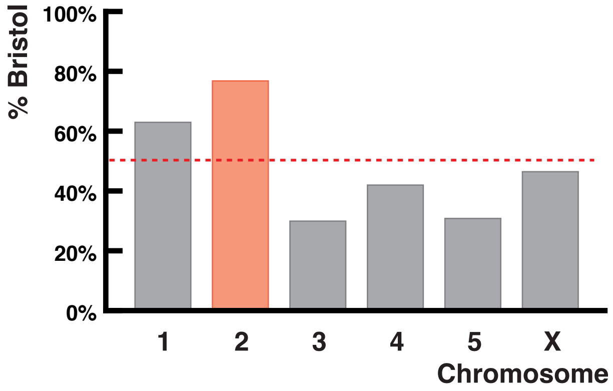

Supplement: Additional data file 2 — Proof-of-principle for chromosomal linkage with 3 known mutations on chromosome 2. Assays used to assess linkage were ZH1-01, ZH2-01, ZH3-05a, ZH4-03, ZH5-01 and ZHX-02 [file gb-2005-6-2-r19-s2.pdf]
