# Supplementary figures and images for: A universal method for automated gene mapping
Source: Genome Biol. 2005 Jan 17;6(2):R19. doi: 10.1186/gb-2005-6-2-r19 (PMC551539; doi:10.1186/gb-2005-6-2-r19)

### Supplementary Figure 2: Mapping of *let-23* to its subchromosomal region

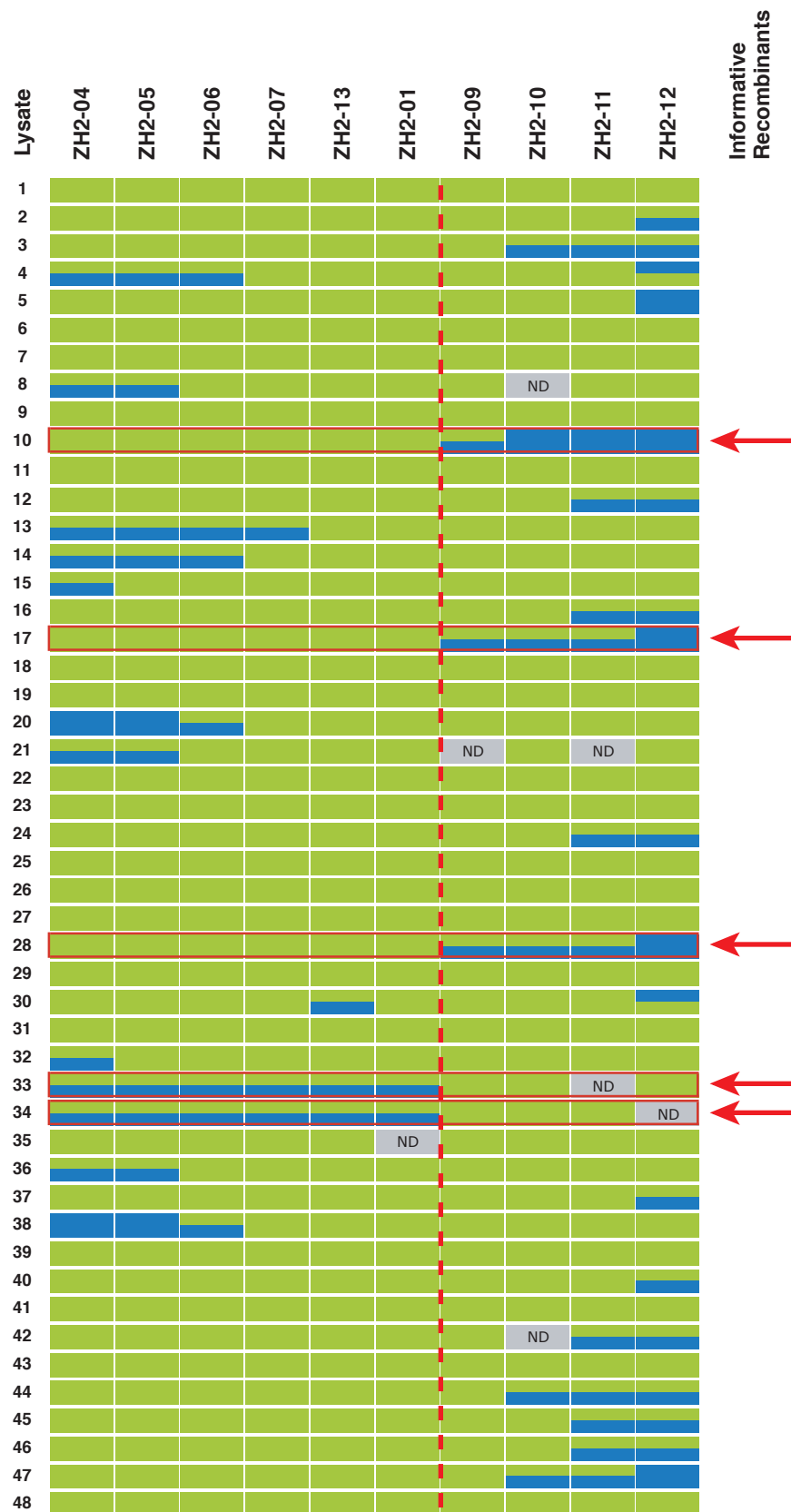

Supplement: Additional data file 3 — Mapping of let-23 to its subchromosomal region (C. elegans) [file gb-2005-6-2-r19-s3.pdf]

### Supplementary Figure 3: Mapping of *rol-1* to its subchromosomal region

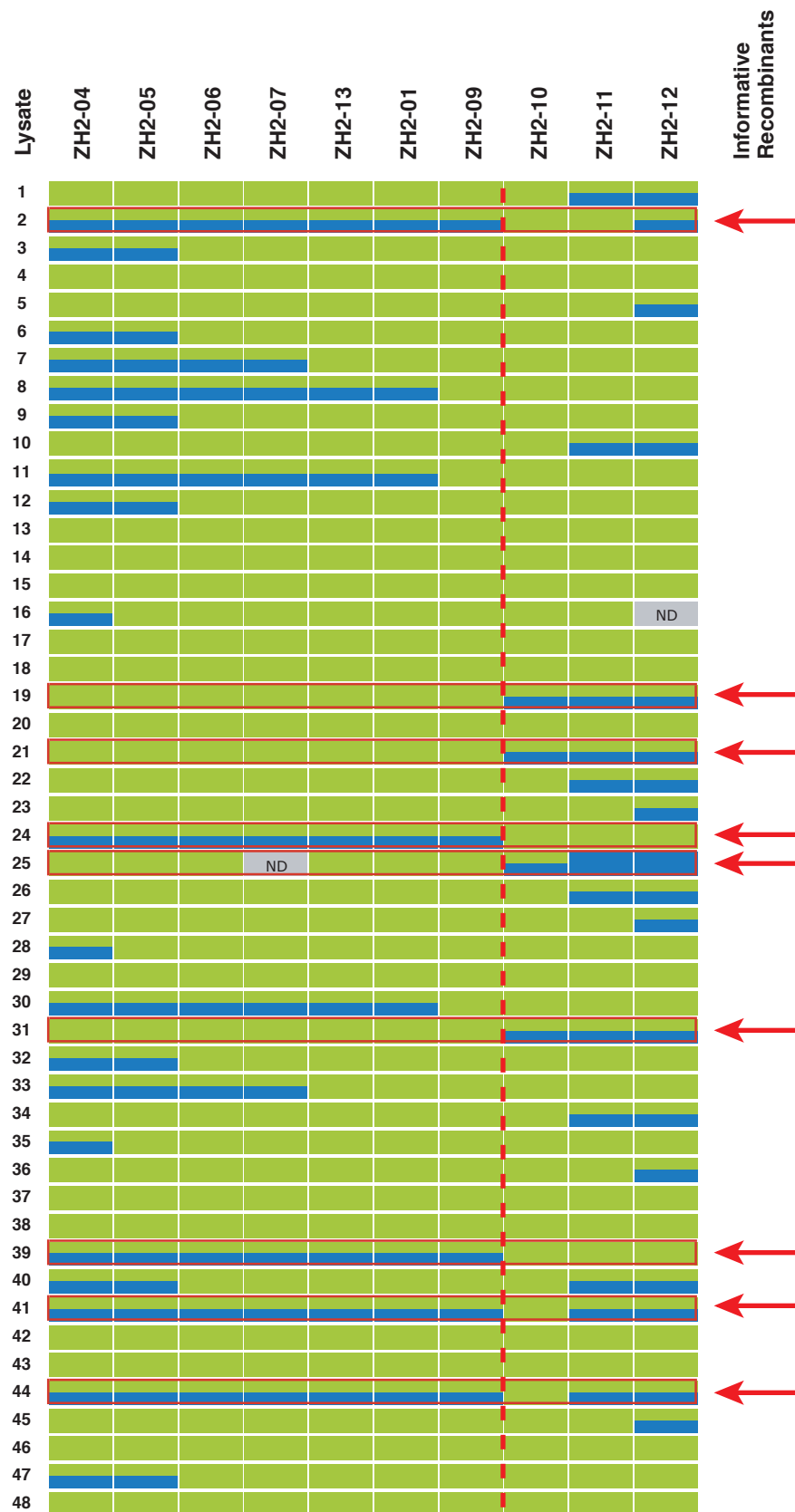

Supplement: Additional data file 4 — Mapping of rol-1 to its subchromosomal region (C. elegans) [file gb-2005-6-2-r19-s4.pdf]

**Supplementary Figure 4: Mapping of *unc-52* to its subchromosomal region**

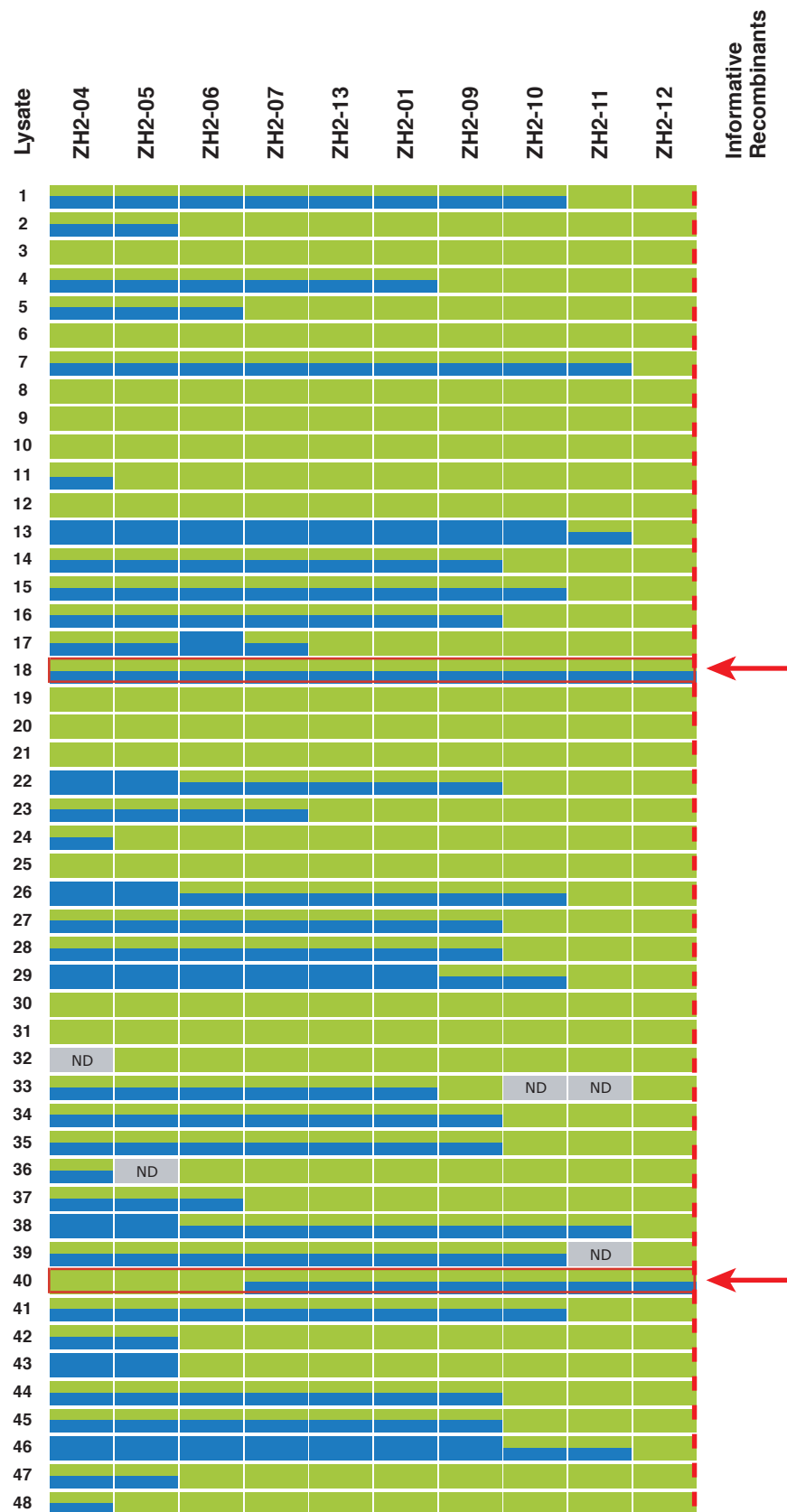

Supplement: Additional data file 5 — Mapping of unc-52 to its subchromosomal region (C. elegans) [file gb-2005-6-2-r19-s5.pdf]

**Supplementary Figure 5: *C. elegans* FLP Mapping Flow Chart**

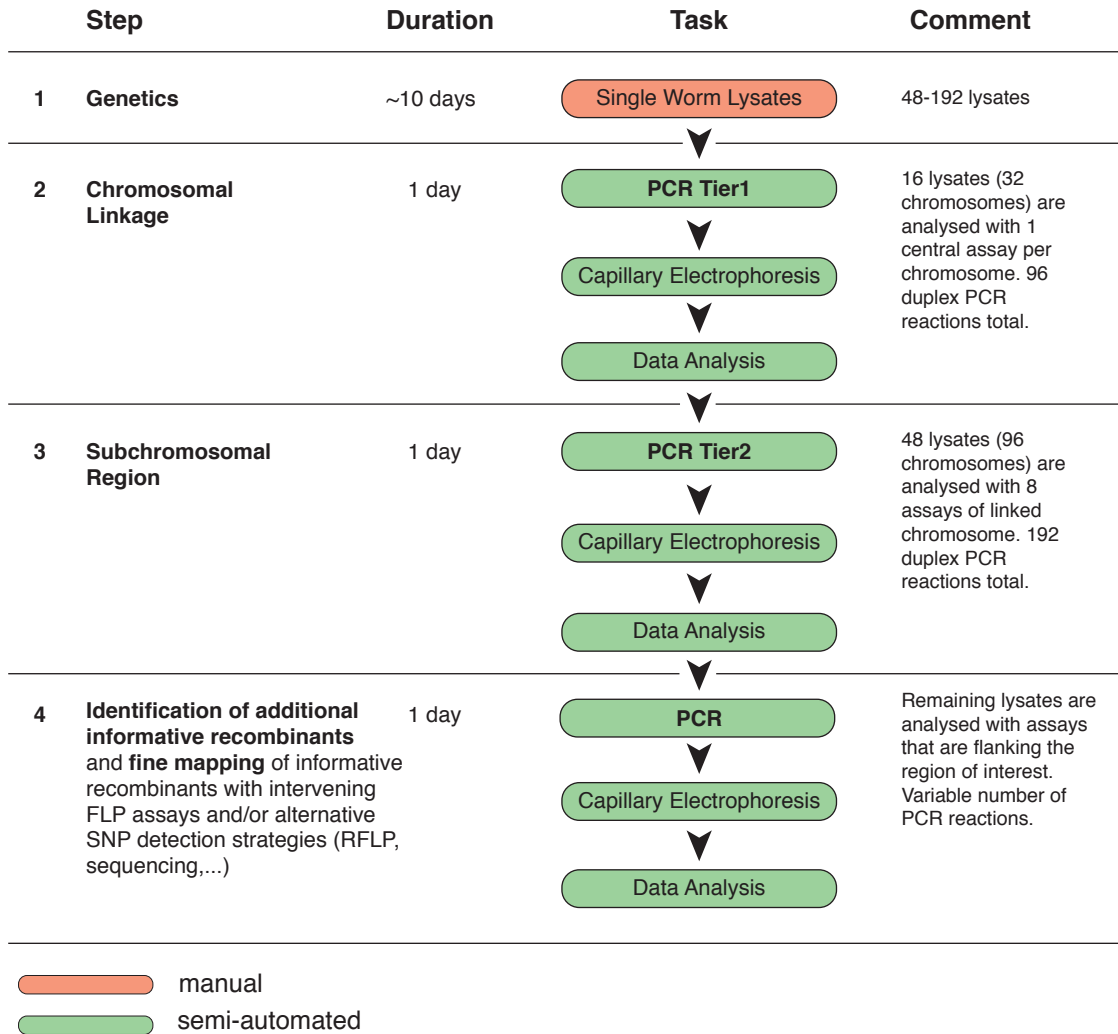

Supplement: Additional data file 6 — C. elegans FLP mapping flow chart [file gb-2005-6-2-r19-s6.pdf]
