# Supplementary material for: A universal method for automated gene mapping
Source: Genome Biol. 2005 Jan 17;6(2):R19. doi: 10.1186/gb-2005-6-2-r19 (PMC551539; doi:10.1186/gb-2005-6-2-r19)

Supplementary Figure 6: *Drosophila* FLP Mapping Flow Chart

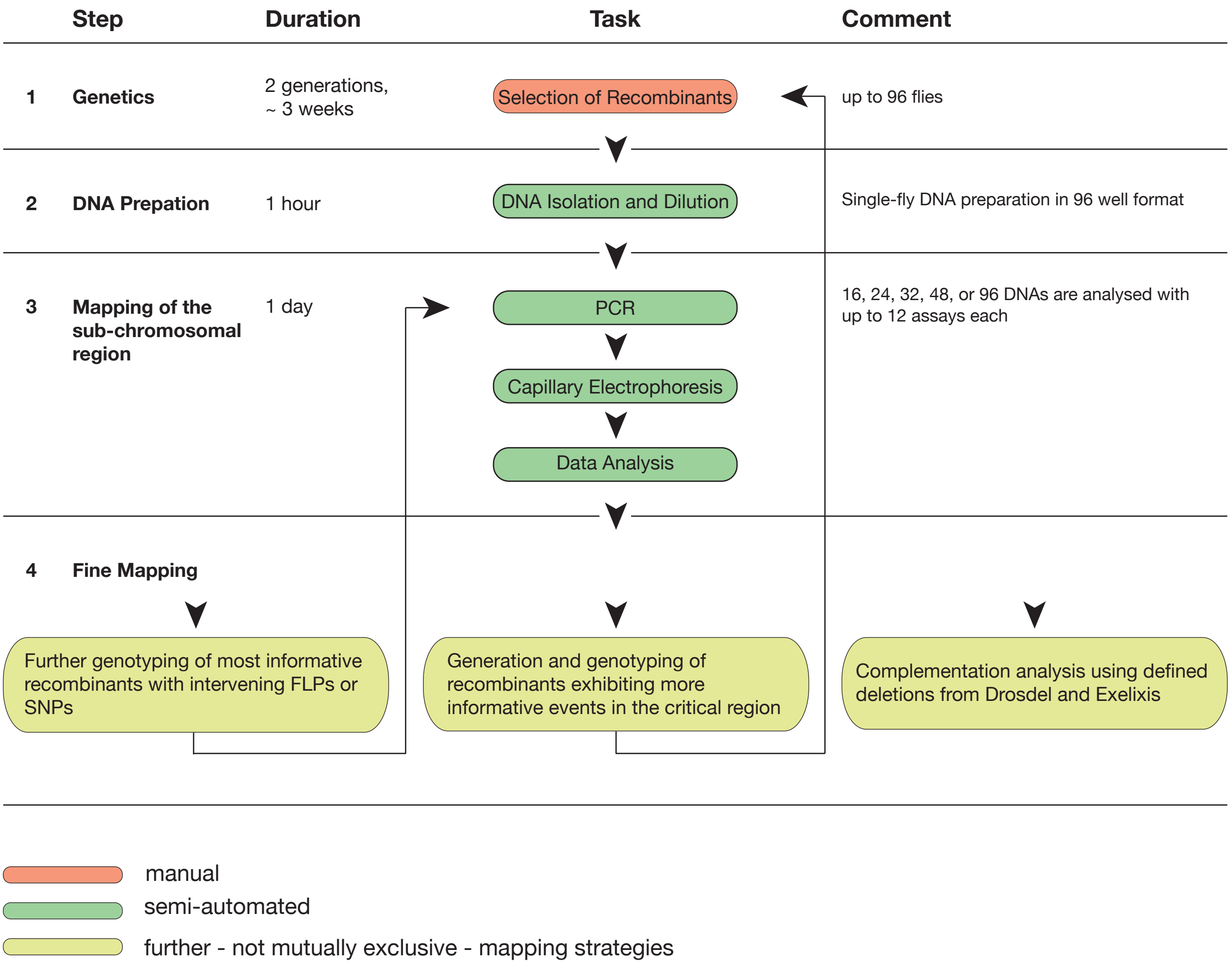

Supplement: Additional data file 7 — Drosophila FLP mapping flow chart [file gb-2005-6-2-r19-s7.pdf]
