# Supplementary material for: A universal method for automated gene mapping
Source: Genome Biol. 2005 Jan 17;6(2):R19. doi: 10.1186/gb-2005-6-2-r19 (PMC551539; doi:10.1186/gb-2005-6-2-r19)

Supplementary Figure 7: Accuracy of Allele Calling

C. elegans ZH5-16

genotype: Bristol
FLP: ZH5-16
allele length: 112 bp

genotype: Hawaii
FLP: ZH5-16
allele length: 113 bp

genotype: Bristol/Hawaii
FLP: ZH5-16
allele length: 112/113 bp

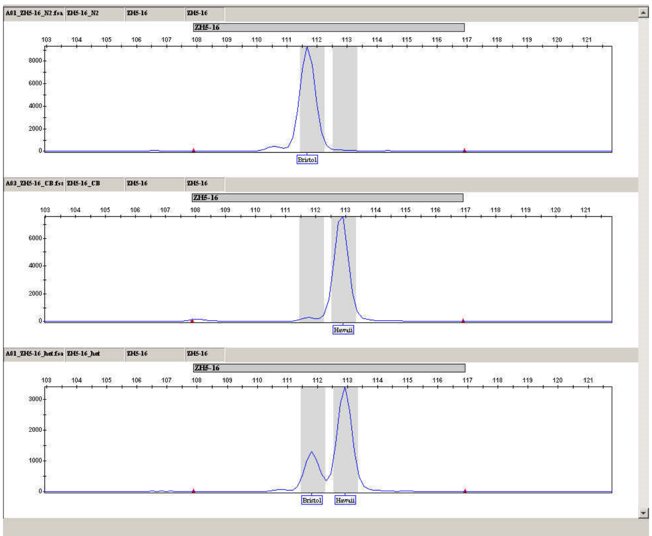

Drosophila 2R090

genotype: EP2R
FLP: 2R090
allele length: 127 bp

genotype: FRT2R
FLP: 2R090
allele length: 128 bp

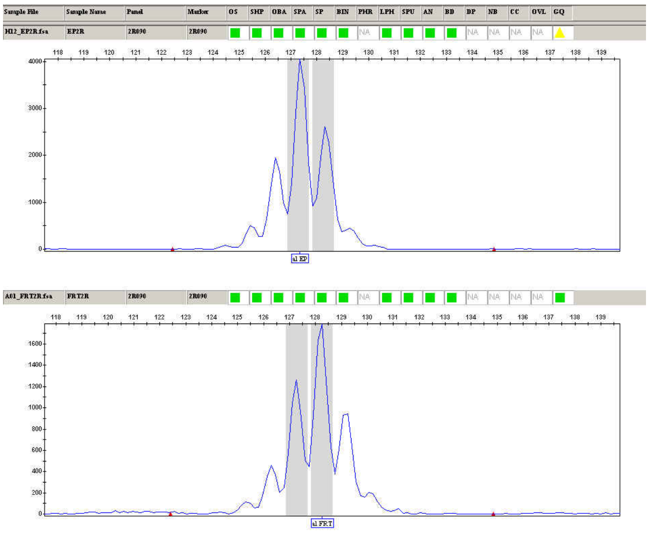

Supplement: Additional data file 8 — Electropherograms demonstrating the accuracy of allele-calling [file gb-2005-6-2-r19-s8.pdf]
