# Supplementary material for: A universal method for automated gene mapping
Source: Genome Biol. 2005 Jan 17;6(2):R19. doi: 10.1186/gb-2005-6-2-r19 (PMC551539; doi:10.1186/gb-2005-6-2-r19)
Supplement: Additional data file 9 — Tables of primer and sequence data of experimentally verified FLP assays in C. elegans [file gb-2005-6-2-r19-s9.pdf]

Supplementary Table 1: Verified *C. elegans* FLP Assays

Chromosome	Assay Name	WormBase SNP	Position on Chromosome (nt)	Distance to next FLP Assay LEFT (kb)	Distance to next FLP Assay RIGHT (kb)	Oligo fwd	Oligo rev	Length Bristol (nt)	Length Hawaii (nt)	Change	GeneModels LEFT	GeneModels RIGHT
	ZH1-16	pkP1099	470031	470.0	568.2	TTCTCGTGCAGCTGGAAATGT	TCGGAGCGTGTTTGCATGTATG	444	453	I9	81	90
	ZH1-17	snp_Y34D9[1]	1038236	568.2	686.7	TTTTTCCAGATTTTTTGTGAAAGC	CGCCTTTAAGGTTACTGTAGC	114	115	I1	90	80
	ZH1-10a	snp_Y71G12[15]	1724899	686.7	546.2	TTCAAAAAAGTGGGAAAACTGAGTT	CACTGGGTCTCTGCTATCAACGT	142	148	I6	80	79
	ZH1-25	snp_Y39G10AL[1]	2271065	546.2	392.1	TTGGTACCGAGTCACCACGA	ATTTCGTCCCGAGTCACTACT	158	157	D1	79	84
	ZH1-07	snp_F40E3[4]	2663170	392.1	460.7	AGTAGAGTCTAACAACACTATT	CTACAATGTAGTTCGCAATCT	132	130	D2	84	93
	ZH1-18a	snp_K09H9[2]	3123875	460.7	1433.3	AATATTTGATTGGATCGTCGCA	TTAGAAGTAAATTTGCAGTACCA	240	235	D5	93	315
	ZH1-03	snp_Y119C1B[8]	4557221	1433.3	279.9	GACCCGACTGCCGTTCTAT	CTGCGTCTCCCTATGTTCG	117	111	D6	315	74
	ZH1-27	snp_F57C9[1]	4837079	279.9	2607.6	CACAGAAGTTTCGAAGTTACAG	AAATTGAGGTGGTATTTTCTCAT	222	223	I1	74	731
	ZH1-34	snp_ZK524[2]	7444723	2607.6	271.0	GAAAAACAATCGCTACATAAAC	GCCTACACAGAAGAATTATGG	191	192	I1	731	62
**I**	ZH1-21	snp_F27D4[1]	7715727	271.0	701.2	CAAAATTTCGCGTCTGGGTGAT	TTGGAAGATGGGGATGCACAAT	176	169	D7	62	157
	ZH1-01	snp_F02E9[1]	8416904	701.2	871.1	TTTTGCGCGTCAAATATGTTGT	TGCTTTAACCTATATTTTGAATGCG	119	122	I3	157	229
	ZH1-22	snp_F14B4[1]	9287981	871.1	511.7	CTCGTTGTGAGACTCTATGGAA	TCCATATTTTTTGGCTTCTGAGA	109	113	I4	229	149
	ZH1-23	snp_F26E4[4]	9799636	511.7	915.1	ATCAAATTTCTCTTGCCTTAATGAT	TAGGTGATTCATTTTTGCGGGAA	201	203	I2	149	220
	ZH1-15	snp_B0205[2]	10714726	915.1	616.5	GGCATTTTTTTGGTTCAACATTT	CGAAGACTTCCTAAAAGAGTGGCA	198	194	D4	220	130
	ZH1-05	snp_H25P06[2]	11331205	616.5	966.3	GAAATCGATTCTTCATTCAGTT	GCCCCTGAAACCCGTGGT	328	348	I20	130	233
	ZH1-08	snp_F56H6[4]	12297522	966.3	533.5	GGGGAAAGCAAGATAGACTC	CGCTTCCGAACAACCACCT	285	292	I7	233	156
	ZH1-09	Y18D10[5]	12831064	533.5	689.4	CATATCGAAAATCAATCCCGTA	AGGAAAAATAAGCTGACCGAAT	248	244	D4	156	153
	ZH1-06	snp_Y87G2[5]	13520509	689.4	382.9	TCAAGAAAAACGGTCATTATGCA	CATTGTATCGCACATCGAACG	278	286	I8	153	67
	ZH1-24	snp_Y71A12B[2]	13903448	382.9	1175.6	ATGCTGAGCCTCTTACTCACT	ATTTATATCTGGAAGTATCGACTT	217	227	I10	67	237
	ZH2-15	snp_W07E6[7]	492112	492.1	459.6	AAAAATTCAAATGCTGCCTATGAA	ATTTTTAGTTTCAGCACACCCTA	201	205	I4	131	126
	ZH2-04a	snp_C32B5[2]	951699	459.6	1153.8	TAAAAGTGAGCTCTTGCCTCATCA	ATGACGATTTTCACTCGGAATCTAG	274	282	I8	126	350
	ZH2-05	snp_R52[2]	2105518	1153.8	664.2	TTACTAGAAGTTTTTGAACACCA	AAACTAGATTTGAACTCGCATT	155	150	D5	350	184
	ZH2-16	np133	2769668	664.2	541.9	AGTGTATTCGATGCAAGTAGAA	TTCTACAACTCTCCAACCCTG	204	209	I5	184	166
	ZH2-06a	snp_F39E9[2]	3311548	541.9	721.3	TAGCAGACCCCCTCTCGGA	CCGACGAGCAATTCAATTTCA	298	289	D9	166	231
	ZH2-07	snp_K07D4[1]	4032873	721.3	656.9	CCTCCGAGACTATGGTGAA	AATTAAGCAGGCTCTGTCAAT	206	212	I6	231	138
	ZH2-17	snp_F10G7[4]	4689731	656.9	587.0	AATTACGCCTCGCCAGTGAA	CAAAACGAAGACCTGCCGAT	208	204	D4	138	175
	ZH2-13	snp_C54A12[4]	5276767	587.0	979.3	CGTTTATTTGGAACTCCCTCAGA	AGAGAAACGAAAACTAACCCACGT	220	217	D3	175	318
	ZH2-19	snp_F13H8[2]	6256072	979.3	1032.4	AAGAAGGTATAGACTAGCAAGA	CAGTCTCTGTGTCGCCTAGA	229	235	I6	318	319
**II**	ZH2-01	snp_C30G12[2]	7288476	1032.4	524.1	ATTGCTCCAAATGTGCATCTTTT	TTCTAACTTACGGATTTGAGCAATT	136	148	I12	319	149
	ZH2-02	snp_T05A6[1]	7812597	524.1	799.4	CTGAGAGTACATTATCAACATC	ATCTCTCTATCCACGTCACAT	255	263	I8	149	230
	ZH2-20	snp_F28C6[3]	8612029	799.4	571.0	ACATTACAGTAGGAAAGTAGGT	TAGAAATCTGTGCAACAAGGTT	240	231	D9	230	152
	ZH2-25	snp_T23G7[2]	9183058	571.0	1344.6	AGGTCCTTCGGTGGTTCTGA	GCCCAAGGCTTTCTGACAG	218	219	I1	152	362
	ZH2-27	snp_R166[4]	10527682	1344.6	831.7	CCCAGGCAGTGCCAGCCAT	CATTCAGTGATATCAGCAGTG	151	157	I6	362	191
	ZH2-09	snp_B0491[2]	11359409	831.7	446.2	GCGAGAAAATCTCAAGTTACA	GGAGAAGGTGATTAGTCGAT	109	101	D8	191	119
	ZH2-28	snp_F43G6[3]	11805581	446.2	825.0	AAAATTCCAAGAACACCGAGA	CAATTAATCCGTCATCGCTCA	177	173	D4	119	207
	ZH2-10	snp_Y38E10[3]	12630557	825.0	876.7	GTGATATAAAAGATGTCTGGAT	ATCCCGACAGCATGGTGGC	125	143	I18	207	164
	ZH2-11	snp_F54F11[3]	13507241	876.7	537.9	TCATCCAGTTTGTCCATTGAA	CGGAGTTACAGGTGTTTTGTA	162	154	D8	164	125
	ZH2-12	snp_W01G7[2]	14045121	537.9	489.0	CCAACTTATGAACGTGCCAA	GGGAGGTTACAAAGATTAGAA	201	197	D4	125	95
	ZH2-23	snp_C04H5[1]	14534168	489.0	733.9	ACACTTTGTACCCATTTATTGAA	CAACGGTGACGTTTTTCAGAA	201	210	I9	95	124
	ZH3-17a	snp_Y55B1BM[1]	416788	416.8	595.5	AGTGATATAGCTAGATACTTGG	GTCTGCTAATTATTTAGCATACC	157	160	I3	87	143
	ZH3-25	snp_Y34F4[2]	1012306	595.5	752.9	CGTCTGATGTTGCCACATTC	AGTTTGTCATCTCGGAGACC	283	287	I4	143	176
	ZH3-06	snp_Y46E12[6]	1765177	752.9	563.0	TACTGTAGTTAGTCAAGACTAT	GGTGCAGTTACCATTGAGAA	191	192	I1	176	94
	ZH3-07	snp_Y54F10BM[9]	2328170	563.0	633.7	ACATTGGAGACGTTGAATCTT	TTCGGTGATATTTTGGTGGAA	112	109	D3	94	122
	ZH3-08	snp_H05C05[5]	2961870	633.7	480.0	CGGCGGGGTTAGAGGAAT	TTCCGAAAATGTTATAGCGAAT	140	137	D3	122	98
	ZH3-26	snp_K01A11[3]	3441912	480.0	863.7	AGATTTCGAGTGCTTGTACAG	ATCGGTTTCTACTTTGCATGG	226	224	D2	98	178
	ZH3-28	snp_H38K22[1]	4305611	863.7	516.5	TAATACCAAGATCACACAGAAC	TTTCTCGGTACAACCAGCGA	184	185	I1	178	149
	ZH3-15	snp_F26A1[2]	4822073	516.5	904.6	ACATTGCGACGAATTTCCTGT	CGCTCACTTGTGGTGGAAAAG	203	202	D1	149	279
	ZH3-04	snp_B0336[6]	5726718	904.6	1533.5	ATCAGCAGAGAATAGTTTCACA	CGAAATGAAGTGGGTATTTGAT	229	232	I3	279	451
**III**	ZH3-02	snp_T21D11[2]	7260267	1533.5	512.2	ACATCACATCATAAGTCAAGC	AAAGTGAGAAATCTTGTCCAC	199	202	I3	451	109
	ZH3-05a	snp_ZK686[3]	7772487	512.2	918.9	CACTGAGGAGACGAGCATTT	AATGAAGATCAGAAATGAGCAA	176	178	I2	109	260
	ZH3-32	snp_F22B7[1]	8691426	918.9	329.7	TATTGTCCACGGTGTTAACAC	TATCTACAAGGACCAGAGCAA	184	186	I2	260	101
	ZH3-35	snp_F59B2[1]	9021169	329.7	1032.9	GAAAACAAGTAATCAATGCTGC	AAGAAGTTTCATGATTAAGGAC	252	253	I1	101	270
	ZH3-10a	snp_T16G12[2]	10054064	1032.9	658.4	GAATTACCGCCATTTGCGTA	GTGGTGAAACGTGTGAAGACT	147	143	D4	270	155
	ZH3-23	snp_W09D10[3]	10712445	658.4	686.8	GCGGTCAGTGCCAGAGCAT	TATGCAATGCAATTAAAAAATGTC	124	125	I1	155	127
	ZH3-11	snp_Y47D3B[3]	11399237	686.8	561.8	TCAACGTAGATCAAGCCGAA	CAACGTAGGTCGCCTTACA	124	126	I2	127	104
	ZH3-12	snp_Y56A3[16]	11961081	561.8	567.1	AAATTGCCTGAAAACACCGAA	CAAGTATTTGGTAAATTGCCAA	196	202	I6	104	90
	ZH3-13	snp_Y111B2[7]	12528166	567.1	1253.4	GTGACGGTGTTTGCAGACG	CATCGGAGTCTGACAGTGTT	229	233	I4	90	239

Supplementary Table 1 (cont.): Verified *C. elegans* FLP Assays

Chromosome	Assay Name	WormBase SNP	Position on Chromosome	Distance to next FLP Assay LEFT (kb)	Distance to next FLP Assay RIGHT (kb)	Oligo fwd	Oligo rev	Length Bristol (nt)	Length Hawaii (nt)	Change	GeneModels LEFT	GeneModels RIGHT
IV	ZH4-04a	snp_Y55F3BR[6]	851112	851.1	889.1	CTTTTTTCCTGGAGTTTCCTAGG	TTCGCAATATTTTTCATCCAAA	265	282	I17	175	164
	ZH4-05	snp_Y41D4[3]	1740251	889.1	1018.4	ACGAAATGAAAAATCAGCAGTT	ATCGCGTGTCGCATACCTT	114	119	I5	164	157
	ZH4-06	snp_Y54G2[2]	2758615	1018.4	1453.6	GCGTAGGCATAGATATAGGT	TTTTCACCCACGTTCAAACAT	147	144	D3	157	320
	ZH4-07	snp_K06B9[6]	4212198	1453.6	369.5	CATTTGGGGCGGTGCTGAA	TTCACGAATGTTTAAGGCGTA	165	172	I7	320	91
	ZH4-16	snp_F28E10[1]	4581704	369.5	1185.0	GGCGTGGGTGTAGGCGTGA	TAAAGATTCTCCGACAATTCCA	246	251	I5	91	309
	ZH4-08	snp_F44E8[2]	5766666	1185.0	1160.1	GCCCGATGTGATCTGTCAT	GGATCGGTTGGAAAATACCA	200	192	D8	309	253
	ZH4-02	snp_T22D1[1]	6926779	1160.1	573.8	AGGAAAGATCATCAGTATGAGA	TGCCCAAACCAAACTGAAAGT	179	186	I7	253	154
	ZH4-03	snp_F55G1[7]	7500574	573.8	557.5	CTGATGCTTTTTTCGGTGTTACAAT	CTTTTGCTGTTGTTTGGATGAA	196	194	D2	154	155
	ZH4-17	snp_C18F3[1]	8058041	557.5	1252.6	TTAAAACTCCAGGCTGCAGTT	GACGTAAACGAATACCGAGTA	168	169	I1	155	329
	ZH4-18	snp_F49C12[1]	9310637	1252.6	523.5	AAATGTTTGCTGCCACATCTG	TTCAACTGATGAGACACGTCT	142	143	I1	329	153
	ZH4-09	pkP4081	9834093	523.5	1338.7	TGATTTTGGCTTATCAGTGAA	CATTGAATAATCCTTTGGAGAA	103	114	I11	153	384
	ZH4-19	snp_C08F8[2]	11172762	1338.7	932.0	CGGAAGTGCTGAGTATTCGT	TCTGTCACGGTTTTGGCACG	170	173	I3	384	227
	ZH4-20	snp_M18[1]	12104785	932.0	807.7	CATCGTGGCTCCATTTCACA	CACCGAAAACATGCAATCAGA	154	155	I1	227	190
	ZH4-10a	snp_K08D8[2]	12912468	807.7	717.0	TCAATTGGCCTGAAATGTAGCA	CCATGTTTCTCTCTCAACTTTTCG	217	211	D6	190	189
	ZH4-21	snp_C08F11[4]	13629499	717.0	615.6	GTTTAACCCCATAATGCTAAACA	CTTTTCTTCGATCACAACGCA	171	166	D5	189	118
	ZH4-11	pkP4094	14245143	615.6	925.8	GTTTTTTAATGTGAAGCAGCAT	TACATACGATTTGATTTACGAT	205	212	I7	118	135
	ZH4-12	snp_Y40H7[3]	15170907	925.8	797.5	ATGTGACAAGTGGTGCAGC	AAGGGGACGGAGGAAAGAT	168	173	I5	135	98
	ZH4-22	snp_Y105C5B[23]	15968421	797.5	1522.0	TATATCGCAACTATCAACCAGT	TATTTGCCCAGAATAATCTCAA	182	185	I3	98	254
V	ZH5-02a	snp_C05E4[5]	749319	749.3	801.7	GGTGCAACGTCACTTCATCAA	TGCAGCCCGGATAGTTCTG	243	261	I18	209	146
	ZH5-13	pkP5101	1551061	801.7	688.4	TTCGATAATCTTTTGATCGCCA	GCGTTTTTGTCATCAACGCAT	194	190	D4	146	189
	ZH5-03a	snp_F41B5[5]	2239502	688.4	1608.6	GGACTACCCATCGTTCCCAGT	CGACTCTCGTGTGCTCGTTG	170	176	I6	189	498
	ZH5-14	snp_F47C10[2]	3848148	1608.6	1333.1	GCTTTCCCTTGTTAGAGTATC	GCGATAACAGGCGACAGAGT	183	190	I7	498	359
	ZH5-04	snp_C03A7[4]	5181248	1333.1	366.8	GCTGAAGGAGATTGACCGTA	TTCCAGCAAAATCGTCGCAA	173	169	D4	359	86
	ZH5-15	snp_C18C4[3]	5548085	366.8	1017.3	TGTAATTCAGCACTTTAGACCA	ACCATTCTCAATTACAACGACT	144	145	I1	86	240
	ZH5-05	snp_K04A8[2]	6565411	1017.3	1236.5	TATCAAATCGTAAATCCGACAT	AAACAGACTTACTTCGTTGTT	199	203	I4	240	388
	ZH5-16	snp_ZK742[1]	7801937	1236.5	1585.2	CCCAAATCAGAAATCTTGCCT	GTGGGTTGTTCAGTGGAGGA	112	113	I1	388	472
	ZH5-01	snp_E02C12[8]	9387102	1585.2	621.6	TCTTTAATAGCTTGCCAATCTTCTT	GACATCGAGCTTTTCGCG	122	128	I6	472	180
	ZH5-17	snp_K08H10[1]	10008679	621.6	1275.5	TAGTTTTCCGTTGACGTCTGA	AATACTTGGTTTTAGATACCGTA	195	190	D5	180	345
	ZH5-18	snp_C03E10[1]	11284131	1275.5	546.0	TGTTTCTATATCTGCGTTTCTC	ATCCATATCGTCCTCTAATGC	222	225	I3	345	147
	ZH5-06	snp_D2023[10]	11830176	546.0	1512.8	GGACTGCTGAGATTTGGGA	ATTACAATTTTCCCTACCTGAA	100	95	D5	147	367
	ZH5-11	snp_C50B6[6]	13342985	1512.8	877.0	GGTCGCTGTTTTGGGTGAA	TGCAAAGATCAAGCGTATCCC	186	190	I4	367	233
	ZH5-23	snp_T16A9[2]	14220019	877.0	1210.6	AAGTTTCACAAAATATCTCTAAG	AATATTTCGTATGTTGAGACTG	119	117	D2	233	373
	ZH5-12	snp_Y36E3[4]	15430591	1210.6	671.0	GCGCAAAACTCATATCATTACCAA	AAATCCAACCTGCCATTGTAGTAAA	315	349	I34	373	257
	ZH5-20	snp_F22B8[2]	16101559	671.0	746.0	TGAAGTAGATGAGACTGGCAT	CGTTAATGTTCCGTTCAATTCT	199	197	D2	257	245
	ZH5-08	snp_T20B3[1]	16847598	746.0	1300.1	GTTACGTAGGTAACAATGCTT	GACAATGGCAAAGTGGTTCAT	165	169	I4	245	363
	ZH5-21	snp_C14A6[1]	18147681	1300.1	999.9	ACTACTTGAATATATCAAGGCTA	TGACTACACCTCAACTGATGA	211	193	D18	363	234
	ZH5-09	snp_Y39B6[17]	19147608	999.9	902.5	TTGAGCAACCCAGCTCGAA	AGAAAAATTCGGTGCGATAGT	200	205	I5	234	204
	ZH5-22	snp_Y113G7[6]	20050070	902.5	863.5	GAAAATGTCTGCTATCGTCCA	ACAAAAATTCGGAATCTACACAA	137	147	I10	204	195
X	ZHX-16	snp_C02H7[1]	734252	734.3	476.4	TTTCAACGAGTCATGCGGTGA	AGCATTGCCGCAGGGTTGA	184	186	I2	138	98
	ZHX-17	snp_C46H3[1]	1210652	476.4	880.0	AGACCTACGTTTCCAACTCAA	AACAAGGACGTAGAAAGATACT	159	163	I4	98	183
	ZHX-03	snp_F49H12[2]	2090657	880.0	1066.8	GTAATAAAAATGTGGCGGGTT	ACAGTGTCGCCTATGGCAT	170	165	D5	183	197
	ZHX-08	snp_C15C7[3]	3157420	1066.8	1219.8	GAGATTTTCATAAGCCTGAGA	GGACCGACCTCCCGAGTTT	129	125	D4	197	250
	ZHX-13	snp_R160[2]	4377207	1219.8	515.0	GCAGCGTCAATCTATGGTTGG	TACTGCTTCAGTGGCTCGGAA	131	133	I2	250	104
	ZHX-15	snp_pkP6106	4892210	515.0	1563.2	TGTCCCCATTCTGTCTCAGCT	CAACGGGAATGGGAAAAACC	206	204	D2	104	315
	ZHX-10	snp_K04E7[1]	6455366	1563.2	1376.7	GGGGAATCTTTGAGAGTGAA	TGTCTGATTCATCCCCAGAT	200	207	I7	315	292
	ZHX-02	snp_C54D2[3]	7832035	1376.7	559.5	AATCGGAATTCGATACAACTCAGTT	TTTATGGACCACAAACGGGT	250	258	I8	292	134
	ZHX-24	snp_F41D9[4]	8391581	559.5	1618.5	TAATTCTTACTTCAAGCTCTG	TCACTGCCATGGACATCTGC	212	213	I1	134	345
	ZHX-07	pkP6138	10010095	1618.5	1454.5	TTGTGAACGAACCCTCTCAA	TTTTCGCAGTGGCTCATTCA	193	186	D7	345	300
	ZHX-12	snp_T25C12[4]	11464550	1454.5	937.2	TCGTCCCCATCTCCGTTT	GGAGAAAAGTCCAGGAAAAAAGA	116	110	D6	300	194
	ZHX-11	snp_F17E5[1]	12401713	937.2	887.2	GATGTGATGAATTGACTACCTT	CTCCAAGGATGACCATAACT	175	170	D5	194	162
	ZHX-05	snp_R04D3[5]	13288921	887.2	1140.5	TATGTGTATTGTTAATTTGAGCA	TTTACACTGCTAACACACCTA	117	121	I4	162	245
	ZHX-21a	snp_F23D12[2]	14429469	1140.5	1131.4	GTGTATTTCTTGCTTTGTTTAAC	GTTTCTGTCGTTTTTGCACATC	157	160	I3	245	200
	ZHX-06	snp_C02C6[1]	15560852	1131.4	875.5	TGGGAAAGATGGTGTTGGAA	ACCTTTAAGCAGACCATGAT	156	150	D6	200	142
	ZHX-22	snp_F38E9[2]	16436347	875.5	555.5	GATCTAAAATGGATTCGATGCT	TCTCACTACACCCAACAACTT	209	205	D4	142	99
	ZHX-23	snp_K02H8[1]	16991892	555.5	723.0	AAACGACATCCTTGAGTTTCA	GAAGTTGCGGCAAATAAAGGT	138	134	D4	99	140
