# Supplementary material for: A universal method for automated gene mapping
Source: Genome Biol. 2005 Jan 17;6(2):R19. doi: 10.1186/gb-2005-6-2-r19 (PMC551539; doi:10.1186/gb-2005-6-2-r19)
Supplement: Additional data file 10 — Tables of primer and sequence data of experimentally verified FLP assays in Drosophila [file gb-2005-6-2-r19-s10.pdf]

Supplementary Table 2: Verified *Drosophila* FLP Assays

Chromosome Arm	Assay	Oligo fwd	Oligo rev	Genetic Position	Physical Position	Cytological Position	Length EP	Length FRT	Length of Other Alleles	Note	Designed by
	2L017	GCAAAAGCGTCGATATGACAGC	CATGCGTGAGTTTCCCCCTAC	2-3/4	2L:1675916..1676066	22B4	148	150	149 (ywWG)		this study
	2L030	CCCCCTTGAAAACTCCCTTCATTTT	CACTGGGTTTCCCTGGTTTCTGTTT	2-8/9	2L:3383921..3384100	23E3	158	179			Berger et al.
	2L038	TCGATTTCCATCTAATCAACCTGTG	AAATGAGTTTTGAAGCCATGCG	2-13	2L:4368759..4368910	24E4	150	152			this study
	2L051	TTTACGGCTCTGGCTGGTAGCTC	GAACAGATTACAGGGCATGGTGC	2-17	2L:5565351..5565531	25F1	181	174	178 (ywGT)		this study
	2L057	GCAAAACGAGTAATGGGCTGTAAGCAA	GGATTAGTTTTAGACCAAAATTGGGAGCAAC	2-20	2L:5950022..5950152	26A3	128	149			Berger et al.
**2L**	2L069	GTGGTGCGAAAGATAAACAAAAATAAACTGG	TCATTGATTGAAACGCTGAAGTTTTAAAGG	2-24	2L:7729483..7729625; 2L:7723343..7723485	28C1	151	139		fragment located in perfect repeat, both fragments seem to exhibit the same polymorphism	Berger et al.
	2L075	GTTATGTCTTTCCTCGCCTGTCG	CATAAAAGGCAGAAGGCAGCACA	2-30	2L:8566862..8566967	29E2	99	104			this study
	2L088	TCCTACGACATTAAGTATATGGTTTGCTTTTGG	GCTCCACGTTGACCGACTCACA	2-34	2L:9835196..9835203	30D1	281	250	277 (FRT2R)		Berger et al.
	2L090	TCATGCGCTCGAGTTTTCAGC	CTGAACGATAATCACGAGGAGGG	2-38	2L:10076927..10077038	31A2	109	110			this study
	2L093	CCCTGTGCTTGCTTGTTGAGTGATTTT	CGGAAAACCTTACTTTTTCGTTACGCTCA	2-41.3	2L:10435288..10435462	31E1-2	164	148			Berger et al.
	2L119	CGAAAACTGAGCGCCAACAACTACAGA	AAGTGGCAGGGCGAAAGACCAAA	2-46/47	2L:12921612..12921731	34A2	115	126			Berger et al.
	2L143	GTAGTCCCAGCCCCAGTGTTCCTTC	TTACAAATGCAAATAGTCCCGTCACACATC	2-51	2L:16310964..16311171	35F1	209	191			Berger et al.
	2R017	CTCGACGGTGGGCTGAGATATGAA	TCAACAAACGAAGGAGAGGAAGGAGTG	2-59/60	2R:4570039..4570211	45F3-4	171	141	140 (F42w+cl), 169 (ywWG), 173 (ywGT)		Berger et al.
	2R039	CATCGCCGTTCTGCGTTTCAGTTT	GCCCGCCATTGTTGTTGCTCTTT	2-64	2R:7269220..7269415	48E10	179	195			Berger et al.
	2R051	CAGTCGGCAAATCGATCCGT	GATAAAACCGTTTACATCATTACTCC	2-68	2R:8076748..8076866	49E7	113	115			this study
	2R057	TCAGGCGCATGACAATGTCTATCG	CATTCCGTCAATGGCTTGCCGC	2-69	2R:8963534..8963751	50C9		215			this study
	2R058	AACGCCTTGAATGAAACTCCCACAG	TCGGGGACTATGCGAGGTCAAG	2-69	2R:8994529..8994702	50C9	171				this study
	2R060	ACTGAAAACAGCGGGCCACCAG	TTCTCCGGAGACCCTCAACCTG	2-70	2R:9182178..9182320	50D7	143	149			this study
	2R061	ATGTGCTTATCACACATGACTTACG	GATCATACAGGCCTTGAAAATCACG	2-70	2R:9287368..9287674	50E4		312			this study
	2R068	TCACCCTTAAATAGTTTGTGTTGGTCTGAAAA	TGGGCAGCAATGACAACAATAGACC	2-73	2R:10139945..10140151	51D12	206	200	213 (ywGT)		Berger et al.
**2R**	2R083	TCACAAGCCCCTCAACCAATACCATC	CGATGGCTTTCAAGGGCACACA	2-77/78	2R:11749308..11749499	53C11	191	179	190 (F42w+cl)		Berger et al.
	2R090	ATCTTATGGCCAACGATGATTCAAC	TTTTGTGTTCCGCCTCAGCTT	2-80	2R:12466048..12466182	54B5	128	129			this study
	2R096	CAGGATTGATGGAGTTTATTGCTTTGCT	TGTAAAAATGTAAGTGTAGGGCTTCCGTGT	2-84	2R:13256746..13257052	55B12	352	306			Berger et al.
	2R109	CCTGCCTCCATGCACTGAAAGAAAT	TGTCTGCACAGGGAAATCAGATCAAG	2-90	2R:15199874..15200115	56F6	225	240	238 (ywWG), 227 (EP2L)		Berger et al.
	2R114	TGACCCAAATAAGAAGAAGAGCACC	TCTCGCAGCCATTTTCTGTGTT	2-93	2R:15540995..15541302	57A2-3	310	298	312 (ywWG)	PLP assay from Berger et al. yields equally sized fragments	this study
	2R118	CTCGGGTTGTTTGGGTCAGTTC	GGCTCGTCAATGGGAAAAGTGC	2-97	2R:16117936..16118168	57B16-19	227	226	233 (F42w+cl)		this study
	2R124	TCTCCTGCCTTACATCAGCTC	ATAAGCAGCTGCAAAAAGGTGC	2-99	2R:16840885..16841075	58A1	191	205			this study
	2R130	GGACTCCGCCAATGAAATTGATT	CCGAACTTGGACGAGGAGTCG	2-102	2R:17944246..17944461	59B4-6	215	232	224 (F42w+cl)		this study
	2R139	AAGTAGAGAACTTTCGTATCAGCCCCCTTGA	AGTGACGCAGGCAAAATCGGAGAA	2-105	2R:18613182..18613396	59F1-4	215	232			this study
	3L021	AACAATAAATACTTGGCGGTTGGCATGG	CAGTGCGAGTGTGTGTGCGTCTGT	3-4.4	3L:2807890..2808083	63A1-2	204	193	198 (ywWG)		Berger et al.
	3L031	CGGCGGCTAACATGAACTTATAAAGGTTTTC	GGGGAATCGGAAGATAGCTCACTAGGTTTT	3-6?	3L:3756972..3757142	63E8-F1	188	167	170 (ywWG)		Berger et al.
	3L041	TTGCACTGCGCCTCATTTTCATTTTT	CCAACCCACTCGCCCTCTCTTATTTTC	3-16?	3L:5182688..5182855	64C9-11	193	164			Berger et al.
	3L058	GTCGTTCAAGGTGGTGTATGAGTATGAG	AAACATGATGTGGACCAGAACGACAA	3-21/26?	3L:6563657..6563786	65B4-5	127	141	142 (ywGT)		Berger et al.
	3L064	GGCAAGTATCAAAGTTCACTTATTTCCCCAAAC	GACTACCACATACCCGTCACCCGAAA	3-23/25.8?	3L:7022923..7023082	65E2-3	158	135	159 (ywWG)		Berger et al.
**3L**	3L076	CTGCATATTAGTTGGCTCCTGG	ATGAATTACTCATGACCGCTACAC	3-24.5/26.5?	3L:8220820..8220996	66C1	170	174	176 (ywGT)	PLP assay from Berger et al. yields equally sized fragments, does not amplify from FRT3R	this study
	3L083	TTACACGGCCAGCAAACGATT	AATGCCAAACCAATAAGCTGGAA	3-27?	3L:9256894..9257245	67A4	365	359	354 (ywWG)		this study
	3L086	CGAGAAATCACAAGTAAATAGCTCCAAAGCTGAA	TGGTATTATATGGCAATGGAACGCGACA	3-28.9	3L:9701662..9701862	67C5	200	216			Berger et al.
	3L094	CAAGGGGCCGCAAATAATAAGAGTAACAGA	ACTTTGGCCCACAAAACCGACAAAC	3-34	3L:10614298..10614501	67E5	193	203			Berger et al.
	3L105	GTTTGCGAGGCGACATCCGACA	GGCTAACTGGCCCATACATTATTTGGCTTT	3-37/38	3L:12442262..12442489	69C2	227	208			Berger et al.
	3L127	GCAACAAAACCAACAGACGCTCACA	GAAAATAAATGAGGAAAATCGCCGCAAC	3-42/43	3L:15416126..15416338	71C2	197	209			Berger et al.
	3L148	GGTGCACTCACAGAAAACAGGTATG	CCCCAGATCCATTCTACTAGTCGC	3-45	3L:17527301..17527637	74D2-3	338	333			this study
	3R061	GCACATGAAACATGAGACATACATTTTAAACAAA	TCGAAATTGCCGACCAAAATAGTGA	3-51	3R:8555097..8555250	87C4	142	152			Berger et al.
	3R074	TTTTAGGAAGGCAACCGCAGC	GTTAGAAGCCTTTCCTCCGCC	3-55	3R:10630472..10630613	88D2	140	141			this study
	3R092	GCGACAGCGCAAAAACTCCTGT	AAGATCATTCTCACGTTCCTCACGATG	3-59	3R:13035430..13035681	89F1	250	215			Berger et al.
	3R122	TGAAACCTGTTTAGGCCTTTTCTCAACCCT	GGGAATGAGGTTAGCAAGTGCGTTTTGT	3-64/65	3R:14763052..14763240	91D3	173	188			Berger et al.
	3R151	CCATGTCGCACTTTCTTTGATATTTGCTTTC	CAAGGCTCACGCACAGGCACTC	3-68	3R:16242420..16242614	92E4	194	210		does not amplify from some strains	Berger et al.
	3R160	CAATGGTGCAGATTAAACGCT	GCTGTGAGCTTCTAGTTGAGT	3-72	3R:17362917..17363093	93E2	175	176			this study
**3R**	3R162	CCTTTACAGACCTCAAACTTAAG	GTTTTCCTGCGTGGGTGAAACG	3-73)	3R:17618105..17618307	93F					this study
	3R169	TTCAAATTAACAAAGAAAATTAGCTCGATTGGTGT	GTGTGAGCGAGCGAGCGAGAAG	3-75/76	3R:18484054..18484270	94B5	198	214			Berger et al.
	3R186	TCCGCTGCGTTGTTAAGATTAGC	GCTATTGCCCACTGTGCCGTA	3-81	3R:19738563..19738693	95C9-11	130	134			this study
	3R192	AGCCAGAGTTTTGATCCCCACAT	CGAGCTGTGGTCGCCTTG	3-86	3R:20556900..20557073	96A18-19	178	173			this study
	3R204	TGCCCTTATTATGTGACCCCAAAAACT	TCCTTTGATCGTTTATATCAAGCTTTGGGAAAT	3-90	3R:22075356..22075533	97A4	187	177			Berger et al.
	3R221	CGATTAAAAGGAATGCCCCGCTGT	AGTGTGTGCCTGTGCTCGTGTCTGT	3-95	3R:24907858..24908171	98F5	365	309	311 (ywWG)		Berger et al.
	3R224	GGAAACATCGTGTAGGAGAGTGTCAGAGGA	AGGGCGGGACCAAGAACGGTAA	3-99	3R:25285399..25285586	99A5-8	190	180	187 (ywWG)		Berger et al.
