# Supplementary material for: A universal method for automated gene mapping
Source: Genome Biol. 2005 Jan 17;6(2):R19. doi: 10.1186/gb-2005-6-2-r19 (PMC551539; doi:10.1186/gb-2005-6-2-r19)
Supplement: Additional data file 11 — A table of the refined genetic distances for FLP assays on the right arm of Drosophila chromosome 2 [file gb-2005-6-2-r19-s11.pdf]

Assay	estimated genetic position	estimated genetic distance (cM)	measured genetic distance (cM)
FRT42D	2-56	-	
**2R017**	2-59/60	3-4	3.9
**2R039**	2-64	4-5	5.8
**2R051**	2-68	4	3.9
**2R060**	2-70	2	8.7
**2R068**	2-73	3	3.9
**2R083**	2-77/78	4-5	5.8
**2R096**	2-84	6-7	2.6
**2R109**	2-90	6	3.1
**2R118**	2-97	7	2.1
**2R124**	2-99	2	3.4
**2R130**	2-102	3	3.4
**2R139**	2-105	3	2.4
EP0755	2-108	3	3.0

Supplementary table 3: Estimated (Lindsley and Zimm, 1992) versus measured genetic distances on chromosome 2R

Lindsley, D. L., and Zimm, G. G. (1992). The Genome of Drosophila melanogaster. . Academic Press, NY.
