# Supplementary material for: A universal method for automated gene mapping
Source: Genome Biol. 2005 Jan 17;6(2):R19. doi: 10.1186/gb-2005-6-2-r19 (PMC551539; doi:10.1186/gb-2005-6-2-r19)
Supplement: Additional data file 12 — Additional non-validated FLPs (predicted C. elegans InDels LGI) [file gb-2005-6-2-r19-s12.pdf]

Supplementary Table 4:
Predicted *C. elegans* InDels LGI

(Validated FLP assays are shown in blue)

WormBase SNP	Position on Chromosome (nt)	Distance between InDels (nt)	Assay Name
uCE1-505	72383	-	
snp_Y48G1C[3]	79850	7467	
pkP5173	90809	10959	
uCE1-507	94846	4037	
snp_Y48G1C[4]	106371	11525	
snp_F53G12[3]	116093	9722	
snp_F53G12[4]	116096	3	
snp_F53G12[7]	116408	312	
snp_F53G12[10]	131906	15498	
snp_F56C11[2]	168054	36148	
uCE1-517	183719	15665	
snp_K10E9[2]	196566	12847	
pkP608	265326	68760	
snp_C53D5[3]	282418	17092	
snp_Y48G1[2]	345872	63454	
uCE1-534	382084	36212	
snp_W04C9[1]	468483	86399	
snp_W04C9[2]	469833	1350	
**pkP1099**	470031	198	**ZH1-16**
uCE1-544	540404	70373	
uCE1-546	567752	27348	
uCE1-547	567918	166	
uCE1-549	570815	2897	
snp_Y65B4BR[10]	578443	7628	
uCE1-561	659336	80893	
snp_Y18H1[4]	675021	15685	
snp_Y18H1[6]	689406	14385	
pkP1100	689674	268	
snp_Y18H1[11]	689676	2	
snp_Y18H1[12]	689817	141	
snp_Y18H1[14]	692699	2882	
snp_T06A4[1]	775761	83062	
uCE1-572	778492	2731	
uCE1-573	794467	15975	
snp_Y95B8[2]	849364	54897	
snp_Y95B8[3]	849412	48	
uCE1-580	884659	35247	
snp_Y95B8[4]	906553	21894	
snp_Y95B8[5]	906566	13	
snp_Y95B8[6]	906745	179	
snp_F54A5[2]	984033	77288	
snp_F54A5[6]	989669	5636	
snp_F54A5[8]	990183	514	
pkP5087	993262	3079	
uCE1-584	1011368	18106	
**snp_Y34D9[1]**	1038236	26868	**ZH1-17**
snp_Y34D9[3]	1038705	469	
uCE1-586	1089792	51087	
snp_Y48G8AL[4]	1202918	113126	
snp_Y48G8AR[7]	1272922	70004	
uCE1-591	1451093	178171	
snp_F47G6[1]	1483430	32337	
snp_Y73E7[3]	1627653	144223	
uCE1-606	1644162	16509	
uCE1-612	1657411	13249	
pkP772	1671768	14357	
pkP546	1671777	9	
snp_Y71G12B[2]	1684797	13020	
snp_Y71G12B[5]	1713739	28942	
pkP5136	1715541	1802	
**snp_Y71G12[15]**	1724899	9358	**ZH1-10a**
snp_Y71G12B[16]	1726189	1290	
snp_Y71G12B[19]	1788070	61881	
snp_Y71G12B[24]	1788073	3	
uCE1-621	1825302	37229	
uCE1-627	2108968	283666	
snp_Y37E3[2]	2153024	44056	
snp_F12B6[3]	2254614	101590	
**snp_Y39G10AL[1]**	2271065	16451	**ZH1-25**
snp_Y39G10AR[7]	2383371	112306	
uCE1-642	2419460	36089	
uCE1-644	2420758	1298	
uCE1-658	2533154	112396	
uCE1-684	2632487	99333	
snp_F40E3[1]	2635214	2727	
pkP971	2660318	25104	
**snp_F40E3[4]**	2663170	2852	**ZH1-07**
snp_F32B5[2]	2664312	1142	
snp_F32B5[4]	2690599	26287	
snp_F32B5[6]	2690887	288	
pkP5107	2746242	55355	
pkP647	2746800	558	

uCE1-711	2805736	58936	
uCE1-724	2806985	1249	
snp_W03D8[1]	2809886	2901	
pkP5281	2816135	6249	
uCE1-742	2822891	6756	
uCE1-743	2834516	11625	
uCE1-752	2852680	18164	
uCE1-753	2880331	27651	
snp_Y71F9AL[2]	2908353	28022	
snp_Y71F9AL[3]	2908539	186	
snp_Y71F9AR[1]	2948948	40409	
pkP559	2972838	23890	
pkP810	2972849	11	
pkP881	2972853	4	
snp_Y54E10BR[1]	3018918	46065	
snp_M01B12[2]	3061728	42810	
**snp_K09H9[2]**	3123875	62147	**ZH1-18a**
snp_Y54E10[3]	3165696	41821	
snp_Y54E10[4]	3177032	11336	
snp_Y54E10[8]	3177302	270	
snp_Y54E10[9]	3177481	179	
snp_Y54E10[12]	3181386	3905	
snp_Y54E10[14]	3181459	73	
snp_Y54E10[15]	3259792	78333	
snp_Y47G6A[4]	3478038	218246	
snp_Y47G6A[5]	3489536	11498	
uCE1-778	3561396	71860	
uCE1-780	3561649	253	
snp_Y47G6A[11]	3573241	11592	
uCE1-781	3587946	14705	
pkP1094	3618019	30073	
pkP1108	3618019	0	
uCE1-792	3803610	185591	
uCE1-793	3806352	2742	
uCE1-795	3840469	34117	
uCE1-798	3911814	71345	
uCE1-800	4043823	132009	
snp_C09D1[1]	4046686	2863	
uCE1-805	4078287	31601	
uCE1-807	4080037	1750	
snp_C24G7[1]	4098818	18781	
pkP990	4128482	29664	
uCE1-815	4231070	102588	
snp_C41D11[2]	4449456	218386	
snp_C41D11[4]	4449652	196	
snp_Y119C1B[2]	4526895	77243	
pkP5100	4551961	25066	
**snp_Y119C1B[8]**	4557221	5260	**ZH1-03**
pkP895	4578572	21351	
uCE1-847	4584756	6184	
snp_D1007[4]	4588185	3429	
snp_T04D1[1]	4672603	84418	
snp_T04D1[2]	4673042	439	
uCE1-850	4677783	4741	
uCE1-857	4761814	84031	
pkP547	4796425	34611	
pkP823	4796426	1	
pkP775	4796435	9	
pkP811	4796437	2	
snp_F07H8[1]	4821897	25460	
**snp_F57C9[1]**	4837079	15182	**ZH1-27**
pkP937	4859345	22266	
pkP5178	4912890	53545	
pkP5019	4912943	53	
uCE1-861	4939092	26149	
pkP5002	5037030	97938	
uCE1-867	5052010	14980	
uCE1-868	5052032	22	
pkP5089	5075778	23746	
pkP967	5075781	3	
pkP536	5156607	80826	
uCE1-870	5172304	15697	
snp_T05E8[1]	5471780	299476	
snp_C09D4[2]	5485627	13847	
pkP5319	5493009	7382	
snp_F59A3[1]	5518144	25135	
uCE1-880	5531369	13225	
snp_F55F8[1]	5666874	135505	
snp_F56H1[4]	5765336	98462	
uCE1-891	5815437	50101	
uCE1-896	5836926	21489	
pkP5312	5896720	59794	
snp_B0207[2]	5948722	52002	
snp_B0025[1]	6030811	82089	
uCE1-900	6035832	5021	
pkP5510	6100854	65022	
pkP571	6100856	2	
pkP5026	6160218	59362	
snp_C48E7[3]	6271415	111197	
snp_F26B1[2]	6340071	68656	
snp_F26B1[3]	6340207	136	

uCE1-910	6476163	135956	
uCE1-911	6532900	56737	
snp_D2092[1]	6620138	87238	
pkP5113	6664947	44809	
pkP5242	6696792	31845	
snp_T23B3[1]	6709909	13117	
pkP592	6875317	165408	
uCE1-920	6910016	34699	
pkP5039	6937709	27693	
snp_T10B11[1]	6945315	7606	
pkP5164	6985989	40674	
pkP5474	7036908	50919	
uCE1-926	7442830	405922	
**snp_ZK524[2]**	7444723	1893	**ZH1-34**
snp_T28F4[1]	7487429	42706	
snp_T23G11[1]	7693326	205897	
snp_T23G11[6]	7696742	3416	
**snp_F27D4[1]**	7715727	18985	**ZH1-21**
pkP550	7808902	93175	
uCE1-946	7902970	94068	
snp_F20G4[1]	7926642	23672	
snp_H05L14[1]	7987989	61347	
pkP5212	8117273	129284	
uCE1-962	8145716	28443	
pkP596	8152963	7247	
pkP5268	8152967	4	
pkP652	8166790	13823	
pkP935	8201816	35026	
snp_F08A10[1]	8206743	4927	
pkP979	8284991	78248	
uCE1-968	8366776	81785	
pkP5121	8373617	6841	
pkP5282	8381920	8303	
**snp_F02E9[1]**	8416904	34984	**ZH1-01**
snp_F10D11[1]	8446109	29205	
snp_K07A12[3]	8675599	229490	
pkP5295	8680885	5286	
pkP611	8680889	4	
pkP1081	8750798	69909	
snp_DY3[2]	8795314	44516	
uCE1-992	8798056	2742	
uCE1-994	8805751	7695	
pkP5061	8887036	81285	
pkP5207	8903719	16683	
pkP5083	8903729	10	
pkP5463	8903733	4	
pkP548	8903733	0	
pkP765	8903745	12	
snp_T08G11[3]	8908060	4315	
snp_F53B6[2]	8965822	57762	
uCE1-1008	9082259	116437	
uCE1-1014	9166001	83742	
uCE1-1017	9252504	86503	
uCE1-1018	9255610	3106	
**snp_F14B4[1]**	9287981	32371	**ZH1-22**
uCE1-1026	9387470	99489	
uCE1-1027	9387541	71	
uCE1-1029	9505176	117635	
pkP694	9559288	54112	
pkP5142	9559299	11	
pkP661	9559301	2	
uCE1-1032	9596055	36754	
snp_K07A1[3]	9607384	11329	
snp_K07A1[4]	9607407	23	
snp_K07A1[6]	9611860	4453	
uCE1-1040	9667316	55456	
pkP5125	9730066	62750	
pkP658	9730068	2	
uCE1-1045	9731699	1631	
snp_K02A11[1]	9747092	15393	
**snp_F26E4[4]**	9799636	52544	**ZH1-23**
snp_F26E4[5]	9800776	1140	
snp_F35C12[1]	9806591	5815	
snp_F35C12[3]	9815991	9400	
pkP662	9837794	21803	
pkP5505	9919872	82078	
pkP780	9919921	49	
pkP600	9925385	5464	
snp_B0379[1]	10101936	176551	
pkP948	10251948	150012	
pkP500	10536362	284414	
pkP911	10540391	4029	
snp_F25H2[1]	10547677	7286	
snp_B0511[1]	10640223	92546	
pkP973	10706656	66433	
snp_B0205[1]	10714404	7748	
**snp_B0205[2]**	10714726	322	**ZH1-15**
pkP5079	10757138	42412	
snp_W02B9[4]	11089437	332299	
pkP677	11149862	60425	
uCE1-1143	11220037	70175	

uCE1-1144	11220086	49	
**snp_H25P06[2]**	11331205	111119	**ZH1-05**
snp_M04D5[2]	11337898	6693	
snp_Y53H1C[4]	11423547	85649	
pkP5081	11454530	30983	
snp_ZK1025[3]	11485375	30845	
snp_K08C9[2]	11502483	17108	
uCE1-1165	11535376	32893	
uCE1-1168	11609124	73748	
uCE1-1178	11666098	56974	
uCE1-1193	11780610	114512	
pkP806	11789522	8912	
uCE1-1202	11813841	24319	
snp_R06C1[1]	11930744	116903	
snp_R06C1[6]	11943599	12855	
snp_R06C1[7]	11943687	88	
snp_Y53C10[6]	11964877	21190	
pkP964	12030642	65765	
pkP5067	12030645	3	
snp_Y53C10[8]	12037177	6532	
snp_F58D5[2]	12055424	18247	
snp_F58D5[3]	12055516	92	
snp_F58D5[4]	12055727	211	
snp_F58D5[5]	12055827	100	
snp_F58D5[6]	12055918	91	
snp_F58D5[10]	12060729	4811	
snp_Y47H10[3]	12101344	40615	
pkP786	12112444	11100	
uCE1-1230	12119748	7304	
uCE1-1232	12119841	93	
uCE1-1240	12120052	211	
pkP840	12131141	11089	
pkP5062	12131147	6	
uCE1-1251	12133061	1914	
snp_F15H9[3]	12159556	26495	
snp_R05D7[2]	12173284	13728	
snp_R05D7[3]	12181594	8310	
snp_R05D7[5]	12181695	101	
snp_R05D7[6]	12181739	44	
uCE1-1258	12211319	29580	
pkP5133	12266061	54742	
**snp_F56H6[4]**	12297522	31461	**ZH1-08**
uCE1-1281	12341002	43480	
snp_T15D6[2]	12391433	50431	
uCE1-1307	12464724	73291	
snp_T27F6[1]	12489219	24495	
snp_K11D2[2]	12513943	24724	
uCE1-1313	12517856	3913	
pkP984	12546877	29021	
snp_T07D10[7]	12618470	71593	
snp_T07D10[8]	12618535	65	
snp_T07D10[9]	12618666	131	
snp_T07D10[17]	12634657	15991	
uCE1-1351	12662760	28103	
uCE1-1353	12683202	20442	
snp_Y18D10[1]	12815266	132064	
snp_Y18D10[4]	12831001	15735	
**snp_Y18D10[5]**	12831064	63	**ZH1-09**
snp_F08A8[2]	12955715	124651	
uCE1-1394	13004498	48783	
uCE1-1397	13036302	31804	
snp_W05B5[2]	13045114	8812	
snp_W05B5[3]	13053203	8089	
snp_W05B5[4]	13053570	367	
pkP5294	13054508	938	
uCE1-1398	13072577	18069	
snp_Y26D4[2]	13092697	20120	
pkP5063	13099701	7004	
pkP589	13099817	116	
uCE1-1401	13148849	49032	
snp_ZK1225[2]	13215608	66759	
snp_ZK1053[1]	13239798	24190	
uCE1-1419	13302215	62417	
uCE1-1430	13426848	124633	
snp_Y87G2[2]	13520505	93657	
**snp_Y87G2[5]**	13520509	4	**ZH1-06**
snp_Y87G2[6]	13555708	35199	
snp_Y87G2[7]	13563507	7799	
snp_Y87G2[8]	13563782	275	
snp_W09C5[2]	13654551	90769	
snp_W09C5[6]	13658235	3684	
snp_W04A4[3]	13700209	41974	
snp_Y6B3B[5]	13759352	59143	
snp_Y6B3B[8]	13759892	540	
snp_W09G3[2]	13811626	51734	
uCE1-1469	13824445	12819	
uCE1-1473	13835277	10832	
uCE1-1475	13835412	135	
uCE1-1480	13839843	4431	
**snp_Y71A12B[2]**	13903448	63605	**ZH1-24**
snp_Y71A12B[6]	13936523	33075	

snp_Y71A12B[10]	13962586	26063
snp_Y71A12B[12]	13962590	4
snp_Y71A12B[14]	13997916	35326
snp_Y63D3[1]	14105158	107242
snp_Y63D3[5]	14108608	3450
snp_ZK849[1]	14197066	88458
snp_ZK849[2]	14197101	35
snp_C49A1[4]	14233584	36483
uCE1-1517	14238345	4761
snp_C49A1[5]	14246019	7674
pkP638	14269486	23467
uCE1-1531	14315424	45938
uCE1-1536	14319843	4419
snp_Y105E8[1]	14345119	25276
uCE1-1545	14354956	9837
snp_Y105E8[4]	14441981	87025
snp_Y105E8[7]	14471366	29385
snp_Y105E8[8]	14475574	4208
snp_Y105E8[9]	14478119	2545
snp_Y105E8[12]	14522468	44349
snp_Y105E8[14]	14548559	26091
snp_Y105E8B[1]	14629567	81008
snp_Y105E8B[2]	14629739	172
snp_K05C4[7]	14755624	125885
uCE1-1571	14775468	19844
snp_F39B2[1]	14785716	10248
snp_Y54E5B[2]	14821362	35646
pkP628	14861007	39645
pkP5036	14877706	16699
uCE1-1589	14907845	30139
uCE1-1595	14913217	5372
uCE1-1598	14914484	1267
pkP5104	14925535	11051
pkP963	14937685	12150
snp_ZK909[1]	14958713	21028
pkP809	14960488	1775
pkP5320	14969576	9088
snp_ZK909[3]	14976505	6929
pkP5213	14978344	1839
pkP633	14978360	16
pkP829	14992612	14252
pkP5123	15001277	8665
pkP700	15001285	8
pkP646	15003663	2378
snp_ZK337[1]	15012909	9246
snp_ZK337[4]	15013311	402
snp_ZK337[6]	15013383	72
pkP5233	15073823	60440
snp_F31C3[3]	15076101	2278
