# Supplementary material for: A universal method for automated gene mapping
Source: Genome Biol. 2005 Jan 17;6(2):R19. doi: 10.1186/gb-2005-6-2-r19 (PMC551539; doi:10.1186/gb-2005-6-2-r19)
Supplement: Additional data file 13 — Additional non-validated FLPs (predicted C. elegans InDels LGII) [file gb-2005-6-2-r19-s13.pdf]

Supplementary Table 5:
Predicted *C. elegans* InDels LGII

(Validated FLP assays are shown in blue)

WormBase SNP	Position on Chromosome (nt)	Distance between InDels (nt)	Assay Name
uCE2-506	97837	-	
snp_T25D3[3]	149696	51859	
snp_C41D7[1]	356968	207272	
snp_C41D7[3]	359065	2097	
snp_C41D7[4]	359254	189	
snp_C41D7[5]	359289	35	
snp_C03H5[2]	401057	41768	
uCE2-519	417497	16440	
snp_W10D9[1]	454697	37200	
snp_W10D9[2]	454980	283	
snp_W07E6[3]	485685	30705	
**snp_W07E6[7]**	492112	6427	**ZH2-15**
uCE2-544	596594	104482	
snp_T04B8[1]	614781	18187	
snp_T02H6[3]	721949	107168	
snp_T02H6[4]	722036	87	
pkP898	785974	63938	
pkP867	785999	25	
pkP517	786000	1	
snp_F28A10[1]	837224	51224	
uCE2-548	863591	26367	
uCE2-553	880593	17002	
pkP653	894216	13623	
pkP5115	895827	1611	
uCE2-555	897376	1549	
uCE2-571	922436	25060	
uCE2-573	922744	308	
snp_R07C3[4]	939538	16794	
**snp_C32B5[2]**	951699	12161	**ZH2-04a**
uCE2-583	951701	2	
pkP676	953821	2120	
pkP5160	953824	3	
uCE2-584	954916	1092	
uCE2-588	955125	209	
uCE2-589	960262	5137	
uCE2-593	974155	13893	
uCE2-600	1001847	27692	
uCE2-614	1002092	245	
snp_M01D1[4]	1028847	26755	
uCE2-634	1040704	11857	
uCE2-650	1041026	322	
uCE2-651	1041046	20	
uCE2-654	1043306	2260	
uCE2-661	1046181	2875	
uCE2-665	1046268	87	
uCE2-713	1160878	114610	
snp_T23F4[1]	1173535	12657	
uCE2-732	1245170	71635	
snp_Y57G7[4]	1301701	56531	
uCE2-752	1332986	31285	
snp_F56D12[4]	1343235	10249	
uCE2-755	1348089	4854	
snp_Y51H7D[2]	1361716	13627	
snp_F54D12[4]	1365966	4250	
snp_F54D12[5]	1365975	9	
uCE2-773	1580725	214750	
pkP5005	1601444	20719	
pkP531	1601447	3	
uCE2-837	1654810	53363	
snp_F58E1[3]	1684180	29370	
uCE2-845	1710314	26134	
uCE2-846	1710513	199	
pkP5217	1724743	14230	
pkP5250	1724751	8	
pkP5290	1724753	2	
pkP985	1724753	0	
snp_F36H5[1]	1748089	23336	
pkP5084	1865381	117292	
pkP561	1865384	3	
pkP673	1937093	71709	
pkP707	1937105	12	
pkP761	1937184	79	
uCE2-957	1941671	4487	
snp_ZK250[3]	1942168	497	
snp_ZK250[4]	1942197	29	
**snp_R52[2]**	2105518	163321	**ZH2-05**
snp_Y59C2[3]	2218627	113109	
uCE2-993	2255074	36447	
snp_F53G2[2]	2452655	197581	
snp_F53G2[3]	2452677	22	
uCE2-1114	2460017	7340	
pkP987	2493562	33545	

snp_F09D1[3]	2575309	81747	
uCE2-1128	2637426	62117	
pkP5307	2693365	55939	
snp_T05A8[1]	2716394	23029	
snp_Y47G7[2]	2745229	28835	
uCE2-1142	2752257	7028	
**np133**	2769668	17411	**ZH2-16**
snp_F08D12[13]	2796991	27323	
uCE2-1159	2809020	12029	
uCE2-1168	2836441	27421	
snp_Y110A2AL[5]	2859617	23176	
pkP2140	2873438	13821	
snp_Y110A2AL[11]	2873822	384	
uCE2-1174	2882602	8780	
uCE2-1186	2887693	5091	
uCE2-1204	2947686	59993	
snp_B0454[4]	3037357	89671	
uCE2-1225	3081104	43747	
snp_Y25C1[11]	3114847	33743	
uCE2-1256	3158034	43187	
snp_Y27F2[2]	3185048	27014	
**snp_F39E9[2]**	3311548	126500	**ZH2-06a**
uCE2-1272	3318033	6485	
uCE2-1274	3318186	153	
snp_T06D4[1]	3389787	71601	
snp_C16C8[2]	3445484	55697	
snp_C16C8[3]	3445754	270	
uCE2-1335	3446821	1067	
snp_C16C8[5]	3447039	218	
uCE2-1338	3448111	1072	
snp_Y49F6B[1]	3499701	51590	
snp_Y49F6B[2]	3499719	18	
uCE2-1376	3576891	77172	
uCE2-1390	3609891	33000	
uCE2-1393	3629156	19265	
snp_M151[1]	3634502	5346	
uCE2-1404	3681064	46562	
uCE2-1415	3681273	209	
snp_F40H7[1]	3694626	13353	
pkP5163	3760855	66229	
snp_F54D10[3]	3811481	50626	
snp_F53C3[2]	3898925	87444	
snp_F53C3[4]	3915114	16189	
snp_F53C3[5]	3915252	138	
snp_K10G6[2]	3976400	61148	
snp_Y14H12[1]	3988499	12099	
snp_Y14H12[2]	3996029	7530	
pkP5273	4016787	20758	
**snp_K07D4[1]**	4032873	16086	**ZH2-07**
uCE2-1526	4104572	71699	
uCE2-1527	4104616	44	
uCE2-1533	4108962	4346	
uCE2-1542	4117875	8913	
uCE2-1551	4145184	27309	
snp_R03H10[2]	4153146	7962	
pkP5030	4211369	58223	
pkP5235	4211440	71	
uCE2-1560	4340881	129441	
snp_B0286[1]	4383987	43106	
snp_B0286[2]	4384137	150	
pkP682	4389403	5266	
pkP632	4442464	53061	
uCE2-1576	4465446	22982	
snp_T05C1[1]	4476856	11410	
pkP5322	4482970	6114	
uCE2-1580	4626107	143137	
uCE2-1581	4661975	35868	
**snp_F10G7[4]**	4689731	27756	**ZH2-17**
uCE2-1594	4846067	156336	
snp_F11G11[1]	4855708	9641	
pkP617	4897028	41320	
snp_F59A6[4]	5013275	116247	
snp_C27A2[1]	5078124	64849	
snp_F58A6[1]	5140103	61979	
snp_C34F11[5]	5218939	78836	
snp_C34F11[6]	5225632	6693	
**snp_C54A12[4]**	5276767	51135	**ZH2-13**
snp_F07F6[1]	5437847	161080	
pkP5017	5445285	7438	
snp_C17G10[1]	5590878	145593	
uCE2-1643	5860742	269864	
snp_F55C12[1]	5865654	4912	
snp_F55C12[2]	5865689	35	
snp_F55C12[4]	5887353	21664	
pkP597	5954430	67077	
uCE2-1655	6084901	130471	
pkP5001	6171346	86445	
snp_C30B5[1]	6199228	27882	
snp_F13H8[1]	6255758	56530	
**snp_F13H8[2]**	6256072	314	**ZH2-19**
pkP5007	6264980	8908	

pkP738	6264986	6	
uCE2-1659	6268998	4012	
uCE2-1662	6381981	112983	
snp_T28D9[2]	6493319	111338	
snp_R05G9R[2]	6507969	14650	
snp_R05G9R[5]	6508586	617	
uCE2-1669	6536429	27843	
snp_C56E6[1]	6539459	3030	
uCE2-1670	6560238	20779	
uCE2-1672	6561612	1374	
snp_F54H5[1]	6631960	70348	
snp_C18H9[1]	6678941	46981	
uCE2-1680	6777131	98190	
uCE2-1693	6860367	83236	
snp_C44B7[3]	6891538	31171	
uCE2-1698	6943032	51494	
snp_C15F1[2]	6958536	15504	
uCE2-1705	7040865	82329	
pkP5162	7171672	130807	
pkP5179	7270260	98588	
**snp_C30G12[2]**	7288476	18216	**ZH2-01**
uCE2-1726	7291804	3328	
uCE2-1734	7338713	46909	
pkP5070	7518208	179495	
snp_C28F5[1]	7521396	3188	
pkP504	7569786	48390	
pkP5391	7657264	87478	
uCE2-1751	7669909	12645	
uCE2-1754	7670179	270	
uCE2-1762	7691814	21635	
snp_B0495[5]	7718834	27020	
snp_C06A8[2]	7787176	68342	
snp_C06A8[3]	7798113	10937	
**snp_T05A6[1]**	7812597	14484	**ZH2-02**
uCE2-1766	7896130	83533	
snp_ZK675[1]	7907599	11469	
snp_ZK669[1]	7926911	19312	
snp_DH11[2]	7994951	68040	
pkP5241	8061477	66526	
snp_T05H10[1]	8061881	404	
snp_K02C4[1]	8078163	16282	
snp_F14F11[3]	8318797	240634	
pkP5009	8382126	63329	
pkP5184	8434851	52725	
snp_F28C6[1]	8596249	161398	
uCE2-1799	8606673	10424	
**snp_F28C6[3]**	8612029	5356	**ZH2-20**
snp_F28C6[7]	8612043	14	
snp_T01B7[2]	8712887	100844	
snp_K08F8[1]	8748241	35354	
snp_F07H5[2]	8784191	35950	
pkP605	8819568	35377	
snp_T07D4[1]	8867971	48403	
uCE2-1805	8896583	28612	
uCE2-1808	8980608	84025	
snp_F37B12[1]	9030066	49458	
uCE2-1812	9062842	32776	
snp_T24B8[3]	9063641	799	
snp_Y9C2U[2]	9147410	83769	
snp_Y9C2U[3]	9147697	287	
**snp_T23G7[2]**	9183058	35361	**ZH2-25**
uCE2-1834	9225830	42772	
snp_C01G6[1]	9266874	41044	
snp_C01G6[4]	9274482	7608	
pkP955	9300427	25945	
pkP5073	9300430	3	
snp_F42A8[1]	9341514	41084	
pkP5187	9380473	38959	
pkP746	9476394	95921	
pkP5170	9522885	46491	
pkP515	9569270	46385	
pkP5299	9589599	20329	
snp_Y53C12B[1]	9739559	149960	
uCE2-1867	9782353	42794	
pkP735	9863370	81017	
pkP758	9863547	177	
uCE2-1872	9964661	101114	
pkP2128	9974009	9348	
pkP2131	10122983	148974	
uCE2-1895	10430269	307286	
snp_E04D5[2]	10433171	2902	
uCE2-1898	10437443	4272	
pkP2071	10497785	60342	
**snp_R166[4]**	10527682	29897	**ZH2-27**
snp_R166[7]	10541680	13998	
snp_R166[5]	10541692	12	
snp_R166[6]	10541705	13	
uCE2-1914	10547923	6218	
snp_C14A4[1]	10612827	64904	
snp_C14A4[2]	10613014	187	
snp_M28[1]	10620583	7569	

snp_M28[3]	10636647	16064	
uCE2-1921	10682103	45456	
uCE2-1924	10686077	3974	
uCE2-1925	10686081	4	
pkP613	10717821	31740	
snp_F44F4[2]	10904044	186223	
snp_F44F4[7]	10914491	10447	
uCE2-1953	11013911	99420	
uCE2-1959	11102433	88522	
snp_W10C6[1]	11112472	10039	
snp_F37H8[2]	11181022	68550	
uCE2-1963	11194986	13964	
snp_F37H8[5]	11196919	1933	
snp_F37H8[6]	11200479	3560	
snp_F37H8[10]	11202381	1902	
uCE2-1964	11207611	5230	
snp_F37H8[13]	11211956	4345	
uCE2-1979	11248882	36926	
uCE2-1981	11258656	9774	
pkP5291	11285493	26837	
uCE2-2001	11328227	42734	
pkP5144	11336041	7814	
uCE2-2002	11337805	1764	
uCE2-2003	11337900	95	
**snp_B0491[2]**	11359409	21509	**ZH2-09**
snp_W02B12[1]	11464057	104648	
pkP5172	11513675	49618	
pkP645	11513677	2	
uCE2-2018	11589515	75838	
pkP5190	11618546	29031	
uCE2-2021	11635289	16743	
snp_ZK20[2]	11635939	650	
uCE2-2029	11711655	75716	
uCE2-2035	11717727	6072	
**snp_F43G6[3]**	11805581	87854	**ZH2-28**
snp_T27D12[1]	11847551	41970	
snp_ZK930[1]	11881394	33843	
snp_ZK930[5]	11881778	384	
uCE2-2049	11889656	7878	
uCE2-2053	11910397	20741	
uCE2-2054	11910420	23	
snp_Y17G7[1]	11917811	7391	
snp_W03C9[2]	11933690	15879	
snp_W03C9[3]	11934427	737	
snp_W03C9[6]	11962048	27621	
snp_Y17G7B[3]	11992569	30521	
snp_Y17G7B[5]	12015395	22826	
snp_Y17G7B[6]	12015668	273	
snp_Y17G7B[8]	12019557	3889	
snp_Y17G7B[10]	12019691	134	
snp_Y17G7B[15]	12029311	9620	
snp_Y17G7B[17]	12043495	14184	
snp_Y17G7B[20]	12043742	247	
uCE2-2083	12113119	69377	
uCE2-2085	12120946	7827	
uCE2-2087	12121104	158	
snp_Y57A10[4]	12157271	36167	
snp_Y57A10[7]	12271356	114085	
snp_Y57A10[16]	12291749	20393	
snp_Y57A10[17]	12291849	100	
snp_Y57A10B[4]	12373114	81265	
snp_Y57A10B[7]	12378329	5215	
snp_Y57A10C[1]	12419061	40732	
snp_F15A4[2]	12471354	52293	
pkP634	12522263	50909	
uCE2-2120	12524599	2336	
pkP656	12540721	16122	
pkP5137	12547484	6763	
uCE2-2129	12579132	31648	
uCE2-2131	12605350	26218	
**snp_Y38E10[3]**	12630557	25207	**ZH2-10**
uCE2-2135	12638486	7929	
snp_Y46G5[1]	12683341	44855	
snp_Y46G5[3]	12683408	67	
uCE2-2143	12704278	20870	
uCE2-2145	12704328	50	
uCE2-2146	12731333	27005	
snp_Y46G5[7]	12760621	29288	
snp_Y46G5[8]	12760667	46	
uCE2-2152	12949655	188988	
snp_Y38F1[4]	12954721	5066	
snp_Y38F1[5]	12989672	34951	
snp_Y38F1[9]	12992659	2987	
snp_Y38F1[15]	13009120	16461	
uCE2-2163	13023490	14370	
snp_F18A11[1]	13034910	11420	
snp_F42G4[3]	13063662	28752	
snp_F42G4[4]	13063702	40	
snp_F42G4[5]	13067250	3548	
snp_F42G4[6]	13073584	6334	
snp_F29C12[5]	13103563	29979	

uCE2-2173	13123522	19959	
snp_W09H1[2]	13179006	55484	
snp_W09E7[2]	13204912	25906	
snp_F15D4[5]	13224179	19267	
uCE2-2204	13261186	37007	
uCE2-2217	13261408	222	
snp_Y48C3[7]	13277106	15698	
uCE2-2219	13279224	2118	
snp_Y48C3[8]	13286388	7164	
snp_Y48C3[16]	13324803	38415	
snp_Y48C3[20]	13334605	9802	
snp_Y48C3[19]	13334676	71	
snp_Y48C3[29]	13367259	32583	
snp_Y48C3[35]	13389058	21799	
uCE2-2228	13410180	21122	
snp_E01G4[3]	13469334	59154	
pkP5171	13502686	33352	
**snp_F54F11[3]**	13507241	4555	**ZH2-11**
snp_Y48E1B[8]	13534983	27742	
pkP2152	13551141	16158	
pkP5272	13590439	39298	
pkP5078	13590551	112	
uCE2-2249	13601053	10502	
snp_F37B1[1]	13617239	16186	
snp_Y48E1[1]	13681635	64396	
snp_Y54G9[1]	13697879	16244	
snp_Y54G9[3]	13722152	24273	
snp_Y54G9[4]	13728728	6576	
uCE2-2260	13729094	366	
snp_R06B9[2]	13750257	21163	
uCE2-2263	13786635	36378	
snp_Y51H1[2]	13886964	100329	
snp_F01D5[5]	14014324	127360	
uCE2-2287	14035762	21438	
**snp_W01G7[2]**	14045121	9359	**ZH2-12**
uCE2-2292	14112068	66947	
uCE2-2293	14132752	20684	
snp_Y48B6[4]	14249035	116283	
snp_R06A4[1]	14360165	111130	
snp_K04B12[1]	14426865	66700	
uCE2-2319	14452054	25189	
uCE2-2322	14453753	1699	
snp_Y54G11B[2]	14461900	8147	
pkP2160	14477693	15793	
pkP941	14480980	3287	
uCE2-2329	14491110	10130	
snp_F57C2[3]	14523899	32789	
**snp_C04H5[1]**	14534168	10269	**ZH2-23**
pkP624	14551462	17294	
snp_F19H8[2]	14614139	62677	
snp_C38C6[3]	14633989	19850	
uCE2-2340	14664515	30526	
uCE2-2349	14682280	17765	
snp_C09F9[1]	14702226	19946	
snp_C09F9[2]	14703838	1612	
snp_C09F9[4]	14703931	93	
uCE2-2364	14757525	53594	
uCE2-2368	14796051	38526	
uCE2-2370	14800038	3987	
snp_R05H10[2]	14830518	30480	
snp_R05H10[3]	14866657	36139	
snp_Y53F4[1]	14879713	13056	
pkP5258	14884584	4871	
pkP982	14885838	1254	
uCE2-2390	14970182	84344	
snp_Y53F4B[9]	15056878	86696	
uCE2-2399	15101231	44353	
uCE2-2402	15101343	112	
snp_Y46E12BM[1]	15212418	111075	
pkP5275	15214135	1717	
pkP607	15272637	58502	
