# Supplementary material for: A universal method for automated gene mapping
Source: Genome Biol. 2005 Jan 17;6(2):R19. doi: 10.1186/gb-2005-6-2-r19 (PMC551539; doi:10.1186/gb-2005-6-2-r19)
Supplement: Additional data file 14 — Additional non-validated FLPs (predicted C. elegans InDels LGIII) [file gb-2005-6-2-r19-s14.pdf]

## Supplementary Table 6:
## Predicted *C. elegans* InDels LGIII

(Validated FLP assays are shown in blue)

| WormBase SNP | Position on Chromosome (nt) | Distance between InDels (nt) | Assay Name |
|---|---|---|---|
| uCE3-513 | 33731 | - | |
| uCE3-514 | 33733 | 2 | |
| uCE3-566 | 138432 | 104699 | |
| snp_K10F12[1] | 360475 | 222043 | |
| **snp_Y55B1BM[1]** | **416788** | 56313 | **ZH3-17a** |
| snp_Y55B1BR[2] | 439866 | 23078 | |
| pkP737 | 447883 | 8017 | |
| pkP5194 | 447886 | 3 | |
| snp_F10C5[1] | 480806 | 32920 | |
| snp_F10C5[2] | 480821 | 15 | |
| uCE3-596 | 513501 | 32680 | |
| uCE3-597 | 513527 | 26 | |
| snp_T22F7[4] | 527346 | 13819 | |
| snp_T22F7[6] | 542885 | 15539 | |
| snp_B0353[1] | 548853 | 5968 | |
| pkP3082 | 593140 | 44287 | |
| uCE3-604 | 596607 | 3467 | |
| uCE3-612 | 617817 | 21210 | |
| pkP5296 | 757704 | 139887 | |
| snp_F42G9[1] | 762554 | 4850 | |
| uCE3-620 | 811729 | 49175 | |
| snp_K02F3[1] | 824859 | 13130 | |
| uCE3-629 | 919876 | 95017 | |
| uCE3-669 | 966881 | 47005 | |
| snp_R06B10[1] | 1002515 | 35634 | |
| uCE3-684 | 1010023 | 7508 | |
| **snp_Y34F4[2]** | **1012306** | 2283 | **ZH3-25** |
| pkP631 | 1021122 | 8816 | |
| pkP5103 | 1021268 | 146 | |
| snp_BE0003N10[7] | 1059457 | 38189 | |
| uCE3-693 | 1061377 | 1920 | |
| uCE3-697 | 1149536 | 88159 | |
| snp_Y92C3[2] | 1169701 | 20165 | |
| snp_Y119D3B[3] | 1199822 | 30121 | |
| snp_Y82E9BL[4] | 1334582 | 134760 | |
| snp_Y82E9BR[2] | 1444255 | 109673 | |
| snp_Y22D7AL[5] | 1619936 | 175681 | |
| uCE3-720 | 1623833 | 3897 | |
| snp_Y22D7AL[7] | 1664214 | 40381 | |
| snp_Y22D7AL[8] | 1664321 | 107 | |
| snp_Y46E12[2] | 1753859 | 89538 | |
| **snp_Y46E12[6]** | **1765177** | 11318 | **ZH3-06** |
| uCE3-723 | 1765994 | 817 | |
| uCE3-726 | 1800038 | 34044 | |
| uCE3-727 | 1800361 | 323 | |
| uCE3-728 | 1803786 | 3425 | |
| snp_B0524[2] | 1892903 | 89117 | |
| uCE3-740 | 1924014 | 31111 | |
| snp_F53A3[4] | 1953982 | 29968 | |
| uCE3-744 | 1954789 | 807 | |
| snp_Y39A3B[1] | 1991227 | 36438 | |
| snp_M01E10[4] | 2044589 | 53362 | |
| uCE3-749 | 2071599 | 27010 | |
| snp_Y48G9[1] | 2091327 | 19728 | |
| pkP5189 | 2119181 | 27854 | |
| pkP5323 | 2119181 | 0 | |
| pkP728 | 2121004 | 1823 | |
| **snp_Y54F10BM[9]** | **2328170** | 207166 | **ZH3-07** |
| pkP3091 | 2356926 | 28756 | |
| pkP3092 | 2357075 | 149 | |
| uCE3-756 | 2419416 | 62341 | |
| pkP5316 | 2432743 | 13327 | |
| snp_E02H9[7] | 2450932 | 18189 | |
| uCE3-760 | 2453398 | 2466 | |
| uCE3-763 | 2487617 | 34219 | |
| snp_Y71H2B[3] | 2599711 | 112094 | |
| snp_Y71H2B[4] | 2622862 | 23151 | |
| snp_Y71H2B[6] | 2622941 | 79 | |
| uCE3-775 | 2655179 | 32238 | |
| snp_Y71H2AL[2] | 2683109 | 27930 | |
| snp_Y71H2AL[3] | 2686253 | 3144 | |
| snp_Y71H2AM[1] | 2705802 | 19549 | |
| uCE3-790 | 2813200 | 107398 | |
| snp_Y71H2AM[5] | 2820248 | 7048 | |
| snp_Y71H2AM[9] | 2823416 | 3168 | |
| snp_Y71H2AM[12] | 2823857 | 441 | |
| snp_Y71H2AM[14] | 2823984 | 127 | |
| uCE3-793 | 2888949 | 64965 | |
| snp_F56F11[2] | 2892486 | 3537 | |
| snp_H05C05[3] | 2945810 | 53324 | |

| | | | |
|---|---|---|---|
| snp_H05C05[4] | 2961628 | 15818 | |
| **snp_H05C05[5]** | **2961870** | 242 | **ZH3-08** |
| pkP724 | 3057240 | 95370 | |
| uCE3-809 | 3067442 | 10202 | |
| snp_C44B11[1] | 3144827 | 77385 | |
| snp_C44B11[2] | 3144993 | 166 | |
| snp_C44B11[3] | 3155107 | 10114 | |
| snp_C44B11[5] | 3164312 | 9205 | |
| snp_H09G03[3] | 3202404 | 38092 | |
| uCE3-815 | 3226966 | 24562 | |
| snp_Y53G8AL[1] | 3242309 | 15343 | |
| uCE3-823 | 3331238 | 88929 | |
| uCE3-831 | 3418406 | 87168 | |
| snp_K01A11[1] | 3439224 | 20818 | |
| snp_K01A11[2] | 3439251 | 27 | |
| **snp_K01A11[3]** | **3441912** | 2661 | **ZH3-26** |
| snp_M01F1[1] | 3520032 | 78120 | |
| snp_C54C6[1] | 3548049 | 28017 | |
| snp_C48D5[2] | 3564730 | 16681 | |
| snp_C48D5[7] | 3567836 | 3106 | |
| uCE3-836 | 3574926 | 7090 | |
| uCE3-839 | 3640946 | 66020 | |
| pkP698 | 3643575 | 2629 | |
| pkP958 | 3752376 | 108801 | |
| snp_ZK1058[1] | 3912036 | 159660 | |
| snp_ZK1058[2] | 3923254 | 11218 | |
| uCE3-848 | 3967322 | 44068 | |
| uCE3-854 | 3992711 | 25389 | |
| pkP753 | 4073230 | 80519 | |
| snp_R10E4[2] | 4279671 | 206441 | |
| **snp_H38K22[1]** | **4305611** | 25940 | **ZH3-28** |
| uCE3-864 | 4347365 | 41754 | |
| pkP524 | 4658420 | 311055 | |
| uCE3-871 | 4685358 | 26938 | |
| pkP689 | 4689683 | 4325 | |
| snp_T04A8[1] | 4704742 | 15059 | |
| uCE3-874 | 4708950 | 4208 | |
| uCE3-879 | 4791474 | 82524 | |
| **snp_F26A1[2]** | **4822073** | 30599 | **ZH3-15** |
| snp_C26E6[2] | 4940855 | 118782 | |
| uCE3-886 | 5013120 | 72265 | |
| snp_K10D2[2] | 5188550 | 175430 | |
| snp_T15B12[1] | 5292947 | 104397 | |
| pkP783 | 5376075 | 83128 | |
| pkP936 | 5376085 | 10 | |
| uCE3-897 | 5382008 | 5923 | |
| pkP5161 | 5473519 | 91511 | |
| uCE3-906 | 5479497 | 5978 | |
| snp_C05D2[1] | 5618848 | 139351 | |
| snp_C05D2[2] | 5619288 | 440 | |
| snp_F54E7[4] | 5666140 | 46852 | |
| uCE3-924 | 5685784 | 19644 | |
| uCE3-925 | 5695323 | 9539 | |
| uCE3-927 | 5706403 | 11080 | |
| **snp_B0336[6]** | **5726718** | 20315 | **ZH3-04** |
| pkP5176 | 5846472 | 119754 | |
| snp_F25B5[1] | 5949546 | 103074 | |
| uCE3-943 | 5978772 | 29226 | |
| snp_T04C9[2] | 5988608 | 9836 | |
| uCE3-948 | 6144499 | 155891 | |
| uCE3-949 | 6195419 | 50920 | |
| snp_T12A2[1] | 6233130 | 37711 | |
| uCE3-950 | 6305028 | 71898 | |
| snp_C56G2[2] | 6354331 | 49303 | |
| pkP601 | 6361299 | 6968 | |
| snp_C16A3[2] | 6399865 | 38566 | |
| snp_C09E7[1] | 6539546 | 139681 | |
| pkP625 | 6585061 | 45515 | |
| pkP721 | 6647336 | 62275 | |
| snp_M01G4[1] | 6670564 | 23228 | |
| uCE3-958 | 6689451 | 18887 | |
| snp_F37A4[1] | 6690436 | 985 | |
| snp_F37A4[2] | 6690447 | 11 | |
| snp_F37A4[4] | 6709451 | 19004 | |
| uCE3-977 | 6951778 | 242327 | |
| snp_F11H8[2] | 7033995 | 82217 | |
| pkP3103 | 7130638 | 96643 | |
| pkP598 | 7210641 | 80003 | |
| **snp_T21D11[2]** | **7260267** | 49626 | **ZH3-02** |
| uCE3-1001 | 7272182 | 11915 | |
| snp_B0361[2] | 7289916 | 17734 | |
| snp_B0361[6] | 7299400 | 9484 | |
| uCE3-1013 | 7387427 | 88027 | |
| uCE3-1027 | 7712930 | 325503 | |
| **snp_ZK686[3]** | **7772487** | 59557 | **ZH3-05a** |
| snp_ZK686[6] | 7772951 | 464 | |
| snp_C27D11[3] | 7821076 | 48125 | |
| uCE3-1041 | 7822731 | 1655 | |
| uCE3-1043 | 7828937 | 6206 | |
| snp_ZK688[3] | 7909137 | 80200 | |
| uCE3-1052 | 7949925 | 40788 | |

| | | | |
|---|---|---|---|
| uCE3-1054 | 7952087 | 2162 | |
| snp_C30A5[1] | 8214538 | 262451 | |
| uCE3-1063 | 8272321 | 57783 | |
| uCE3-1066 | 8324717 | 52396 | |
| snp_C04D8[3] | 8505942 | 181225 | |
| snp_C04D8[5] | 8512789 | 6847 | |
| snp_F22B7[1] | 8641925 | 129136 | |
| uCE3-1081 | 8643040 | 1115 | |
| **snp_F22B7[1]** | **8691426** | 48386 | **ZH3-32** |
| uCE3-1085 | 8692042 | 616 | |
| snp_F44E2[1] | 8846515 | 154473 | |
| snp_K01F9[1] | 8880095 | 33580 | |
| snp_ZK637[1] | 8904225 | 24130 | |
| snp_ZK637[2] | 8904472 | 247 | |
| snp_ZK643[1] | 8935374 | 30902 | |
| snp_ZK643[3] | 8959874 | 24500 | |
| snp_ZK643[3] | 8959874 | 0 | |
| snp_ZK643[4] | 8960287 | 413 | |
| **snp_F59B2[1]** | **9021169** | 60882 | **ZH3-35** |
| uCE3-1095 | 9242561 | 221392 | |
| uCE3-1100 | 9281086 | 38525 | |
| snp_C40H1[3] | 9328240 | 47154 | |
| uCE3-1102 | 9343296 | 15056 | |
| snp_F55H2[1] | 9519920 | 176624 | |
| snp_ZK1098[2] | 9547167 | 27247 | |
| snp_F58A4[1] | 9622150 | 74983 | |
| snp_C15H7[2] | 9639394 | 17244 | |
| uCE3-1115 | 9699809 | 60415 | |
| snp_K11H3[2] | 9853498 | 153689 | |
| snp_K03H1[1] | 9947496 | 93998 | |
| uCE3-1131 | 10031240 | 83744 | |
| **snp_T16G12[2]** | **10054064** | 22824 | **ZH3-10a** |
| uCE3-1135 | 10091061 | 36997 | |
| uCE3-1137 | 10100786 | 9725 | |
| uCE3-1139 | 10100852 | 66 | |
| uCE3-1141 | 10101059 | 207 | |
| uCE3-1144 | 10106220 | 5161 | |
| snp_T20G5[1] | 10188944 | 82724 | |
| uCE3-1145 | 10213724 | 24780 | |
| pkP921 | 10232742 | 19018 | |
| pkP555 | 10232743 | 1 | |
| snp_T07A5[2] | 10316663 | 83920 | |
| uCE3-1167 | 10514375 | 197712 | |
| uCE3-1170 | 10522339 | 7964 | |
| snp_T21C12[6] | 10541589 | 19250 | |
| snp_Y39A1[8] | 10648550 | 106961 | |
| uCE3-1184 | 10649315 | 765 | |
| uCE3-1188 | 10658999 | 9684 | |
| **snp_W09D10[3]** | **10712445** | 53446 | **ZH3-23** |
| uCE3-1199 | 10818078 | 105633 | |
| uCE3-1203 | 10866843 | 48765 | |
| snp_C44B9[1] | 10885472 | 18629 | |
| uCE3-1211 | 11046648 | 161176 | |
| uCE3-1213 | 11079968 | 33320 | |
| snp_W09D6[1] | 11080292 | 324 | |
| snp_Y47D3[9] | 11200270 | 119978 | |
| snp_Y47D3[12] | 11211671 | 11401 | |
| snp_Y47D3[16] | 11249254 | 37583 | |
| snp_Y47D3[19] | 11262469 | 13215 | |
| uCE3-1227 | 11291573 | 29104 | |
| snp_Y47D3[20] | 11335629 | 44056 | |
| snp_Y47D3B[1] | 11398988 | 63359 | |
| **snp_Y47D3B[3]** | **11399237** | 249 | **ZH3-11** |
| snp_Y47D3B[9] | 11413561 | 14324 | |
| snp_Y47D3B[11] | 11427189 | 13628 | |
| snp_Y47D3B[14] | 11439774 | 12585 | |
| snp_Y47D3B[17] | 11443790 | 4016 | |
| snp_Y47D3B[23] | 11465650 | 21860 | |
| uCE3-1238 | 11473559 | 7909 | |
| snp_Y66A7AL[1] | 11473572 | 13 | |
| snp_Y66A7AL[3] | 11473778 | 206 | |
| uCE3-1243 | 11527317 | 53539 | |
| snp_Y66D12[5] | 11591915 | 64598 | |
| snp_Y66D12[6] | 11591927 | 12 | |
| uCE3-1249 | 11614812 | 22885 | |
| snp_Y66A7AR[1] | 11616400 | 1588 | |
| snp_Y66A7AR[4] | 11622027 | 5627 | |
| snp_Y41C4[1] | 11647111 | 25084 | |
| snp_Y41C4[3] | 11678036 | 30925 | |
| snp_Y41C4[6] | 11684500 | 6464 | |
| snp_Y41C4[7] | 11684614 | 114 | |
| snp_Y41C4[12] | 11745100 | 60486 | |
| snp_Y41C4[14] | 11766672 | 21572 | |
| pkP5180 | 11782040 | 15368 | |
| uCE3-1267 | 11801402 | 19362 | |
| uCE3-1268 | 11809018 | 7616 | |
| uCE3-1271 | 11810432 | 1414 | |
| uCE3-1276 | 11819615 | 9183 | |
| uCE3-1278 | 11825534 | 5919 | |
| snp_Y56A3[1] | 11834656 | 9122 | |
| snp_Y56A3[2] | 11834816 | 160 | |

| | | | |
|---|---|---|---|
| snp_Y56A3[4] | 11834893 | 77 | |
| snp_Y56A3[5] | 11834977 | 84 | |
| uCE3-1295 | 11938100 | 103123 | |
| pkP678 | 11960300 | 22200 | |
| **snp_Y56A3[16]** | **11961081** | 781 | **ZH3-12** |
| pkP577 | 12239191 | 278110 | |
| snp_Y75B8[10] | 12302315 | 63124 | |
| pkP674 | 12328072 | 25757 | |
| pkP691 | 12328073 | 1 | |
| snp_Y49E10[1] | 12373466 | 45393 | |
| uCE3-1326 | 12375191 | 1725 | |
| uCE3-1327 | 12375239 | 48 | |
| uCE3-1330 | 12375449 | 210 | |
| snp_Y49E10[6] | 12383864 | 8415 | |
| snp_Y49E10[16] | 12472865 | 89001 | |
| snp_Y111B2[3] | 12496414 | 23549 | |
| snp_Y111B2[5] | 12514734 | 18320 | |
| snp_Y111B2[6] | 12514745 | 11 | |
| snp_Y111B2[4] | 12514802 | 57 | |
| **snp_Y111B2[7]** | **12528166** | 13364 | **ZH3-13** |
| snp_Y111B2[8] | 12528219 | 53 | |
| snp_Y111B2[11] | 12540909 | 12690 | |
| snp_Y111B2[12] | 12583536 | 42627 | |
| snp_Y111B2[15] | 12583675 | 139 | |
| snp_Y111B2[24] | 12607719 | 24044 | |
| uCE3-1346 | 12608492 | 773 | |
| snp_Y111B2[25] | 12617233 | 8741 | |
| uCE3-1357 | 12690132 | 72899 | |
| uCE3-1359 | 12690342 | 210 | |
| uCE3-1361 | 12714346 | 24004 | |
| snp_BE10[6] | 12815920 | 101574 | |
| pkP516 | 12865040 | 49120 | |
| pkP5003 | 12866649 | 1609 | |
| snp_Y37D8[2] | 12889026 | 22377 | |
| snp_Y37D8[3] | 12889036 | 10 | |
| snp_Y39E4[3] | 12952845 | 63809 | |
| snp_Y39E4[7] | 12964685 | 11840 | |
| snp_Y39E4[8] | 12967380 | 2695 | |
| snp_Y39E4[9] | 12967490 | 110 | |
| pkP5191 | 12983325 | 15835 | |
| snp_ZK1010[1] | 13008996 | 25671 | |
| snp_ZK1010[2] | 13008998 | 2 | |
| snp_F14F7[3] | 13020591 | 11593 | |
| snp_F14F7[5] | 13020750 | 159 | |
| snp_F14F7[9] | 13030895 | 10145 | |
| snp_F14F7[10] | 13031201 | 306 | |
| pkP5185 | 13052990 | 21789 | |
| pkP679 | 13052991 | 1 | |
| pkP5011 | 13054631 | 1640 | |
| pkP510 | 13054634 | 3 | |
| snp_F54F12[8] | 13058806 | 4172 | |
| uCE3-1391 | 13106958 | 48152 | |
| pkP774 | 13230714 | 123756 | |
| snp_F56A8[2] | 13253152 | 22438 | |
| snp_F56A8[3] | 13277823 | 24671 | |
| snp_T03F6[2] | 13363592 | 85769 | |
| uCE3-1401 | 13515833 | 152241 | |
| uCE3-1412 | 13591099 | 75266 | |
| snp_ZK525[1] | 13661021 | 69922 | |
| snp_ZK525[2] | 13662035 | 1014 | |
| uCE3-1419 | 13668790 | 6755 | |
| pkP755 | 13699628 | 30838 | |
| snp_W06F12[1] | 13730985 | 31357 | |
| snp_W06F12[2] | 13730987 | 2 | |
| snp_W06F12[3] | 13731298 | 311 | |
