# Supplementary material for: A universal method for automated gene mapping
Source: Genome Biol. 2005 Jan 17;6(2):R19. doi: 10.1186/gb-2005-6-2-r19 (PMC551539; doi:10.1186/gb-2005-6-2-r19)
Supplement: Additional data file 15 — Additional non-validated FLPs (predicted C. elegans InDels LGIV) [file gb-2005-6-2-r19-s15.pdf]

Supplementary Table 7:
Predicted *C. elegans* InDels LGIV

(Validated FLP assays are shown in blue)

WormBase SNP	Position on Chromosome (nt)	Distance between InDels (nt)	Assay Name
pkP659	343527	-	
pkP5041	648670	305143	
uCE4-515	795461	146791	
snp_Y55F3BR[3]	850766	55305	
**snp_Y55F3BR[6]**	851112	346	**ZH4-04a**
snp_Y55F3BR[9]	876560	25448	
snp_Y55F3BR[11]	876859	299	
snp_F58H7[1]	926402	49543	
pkP5072	951635	25233	
uCE4-523	970165	18530	
snp_Y55F3AM[2]	998230	28065	
snp_F53H1[3]	1303617	305387	
pkP506	1336791	33174	
snp_C07B3[1]	1381390	44599	
uCE4-542	1421693	40303	
uCE4-555	1445526	23833	
snp_Y77E11[8]	1468313	22787	
uCE4-562	1471878	3565	
uCE4-563	1479581	7703	
uCE4-568	1490217	10636	
pkP762	1516442	26225	
pkP763	1516442	0	
pkP732	1516531	89	
snp_K03H6[5]	1545757	29226	
snp_Y41D4B[7]	1657546	111789	
uCE4-595	1720581	63035	
snp_K08D12[1]	1721822	1241	
snp_K08D12[2]	1726772	4950	
**snp_Y41D4[3]**	1740251	13479	**ZH4-05**
snp_Y41D4[5]	1751451	11200	
snp_Y41D4[7]	1776140	24689	
snp_Y38C1B[1]	1798725	22585	
pkP5135	1893337	94612	
snp_F55A8[1]	1895197	1860	
uCE4-608	1917378	22181	
snp_F52C12[2]	1925697	8319	
snp_F52C12[4]	1925899	202	
uCE4-612	1925901	2	
snp_F52C12[6]	1942856	16955	
snp_F52C12[7]	1943053	197	
snp_Y76B12C[2]	2006809	63756	
snp_Y76B12C[3]	2006895	86	
snp_Y76B12C[7]	2007176	281	
uCE4-622	2075050	67874	
snp_R05C11[4]	2075396	346	
snp_R05C11[5]	2075563	167	
uCE4-624	2077894	2331	
uCE4-625	2077904	10	
snp_R05C11[9]	2084172	6268	
snp_C54E4[2]	2155922	71750	
uCE4-630	2191571	35649	
uCE4-638	2192046	475	
snp_Y71G10AR[10]	2211321	19275	
snp_T04C4[2]	2221999	10678	
snp_T04C4[4]	2222112	113	
uCE4-653	2249865	27753	
snp_C37F5[4]	2276919	27054	
snp_C37F5[5]	2276938	19	
snp_C37F5[10]	2287057	10119	
snp_C37F5[14]	2288836	1779	
snp_Y38F2AL[7]	2327429	38593	
snp_Y38F2AR[1]	2395493	68064	
uCE4-671	2457353	61860	
pkP5013	2486726	29373	
snp_Y69A2AR[9]	2605436	118710	
snp_Y69A2AR[10]	2605442	6	
snp_Y69A2AR[15]	2641144	35702	
snp_Y69A2AR[17]	2641304	160	
snp_Y94H6[1]	2685005	43701	
uCE4-685	2707865	22860	
uCE4-689	2728544	20679	
snp_Y54G2[1]	2751474	22930	
**snp_Y54G2[2]**	2758615	7141	**ZH4-06**
snp_Y54G2[7]	2761694	3079	
snp_Y54G2[11]	2827196	65502	
snp_Y54G2[20]	2863176	35980	
uCE4-699	2914263	51087	
pkP5257	2924441	10178	
snp_Y54G2[32]	2959569	35128	
snp_Y54G2[35]	2985599	26030	
uCE4-711	3075303	89704	

pkP975	3083506	8203	
snp_Y67D8C[13]	3103368	19862	
snp_Y67D8C[14]	3103687	319	
snp_Y67D8B[3]	3181339	77652	
uCE4-717	3191606	10267	
snp_Y67D8B[6]	3192517	911	
snp_M4[1]	3210951	18434	
uCE4-742	3298860	87909	
pkP583	3328946	30086	
snp_F42A6[4]	3354162	25216	
snp_C04C3[4]	3423528	69366	
snp_C04C3[7]	3423934	406	
snp_C04C3[8]	3423978	44	
uCE4-754	3454928	30950	
uCE4-761	3464645	9717	
uCE4-764	3502591	37946	
snp_Y37E11C[2]	3517982	15391	
snp_ZC416[1]	3636105	118123	
snp_ZC416[2]	3636486	381	
snp_Y37E11AL[3]	3687373	50887	
uCE4-793	3689883	2510	
uCE4-800	3765229	75346	
uCE4-808	3790205	24976	
uCE4-811	3790470	265	
uCE4-820	3903860	113390	
pkP522	3986839	82979	
snp_F47C12[4]	3993642	6803	
pkP602	4006080	12438	
snp_W03D2[1]	4051055	44975	
snp_W03D2[2]	4051205	150	
uCE4-844	4056225	5020	
snp_W03D2[5]	4073991	17766	
uCE4-863	4173254	99263	
**snp_K06B9[6]**	4212198	38944	**ZH4-07**
uCE4-876	4258280	46082	
snp_F15E6[2]	4285492	27212	
**snp_F28E10[1]**	4581704	296212	**ZH4-16**
pkP4035	5061512	479808	
pkP759	5157290	95778	
uCE4-912	5190677	33387	
snp_F36H12[3]	5277130	86453	
pkP5243	5304500	27370	
snp_ZK354[1]	5308929	4429	
snp_Y4C6B[1]	5337761	28832	
snp_F41H10[1]	5373774	36013	
uCE4-915	5463472	89698	
snp_T12E12[1]	5541287	77815	
uCE4-917	5544804	3517	
snp_T12E12[2]	5561674	16870	
snp_T12E12[3]	5561988	314	
uCE4-919	5722646	160658	
uCE4-923	5760219	37573	
**snp_F44E8[2]**	5766666	6447	**ZH4-08**
uCE4-927	5779451	12785	
snp_C31H1[2]	5782062	2611	
pkP513	5795619	13557	
snp_H04M03[1]	5887494	91875	
snp_K08B4[1]	6077556	190062	
snp_C49A9[1]	6208978	131422	
snp_C49A9[2]	6212702	3724	
uCE4-946	6215053	2351	
uCE4-947	6217919	2866	
snp_Y73B6BL[5]	6399193	181274	
snp_Y73B6BL[7]	6414285	15092	
uCE4-951	6445877	31592	
uCE4-952	6447450	1573	
snp_Y73B6BL[8]	6460112	12662	
snp_Y73B6BL[9]	6460259	147	
snp_Y73B6BR[1]	6502095	41836	
pkP701	6532549	30454	
uCE4-958	6591127	58578	
uCE4-962	6641456	50329	
snp_C17H12[1]	6811992	170536	
snp_H20E11[1]	6827182	15190	
**snp_T22D1[1]**	6926779	99597	**ZH4-02**
snp_C34D4[1]	7137860	211081	
uCE4-977	7144697	6837	
pkP5276	7281552	136855	
pkP5024	7434288	152736	
snp_B0496[2]	7444469	10181	
**snp_F55G1[7]**	7500574	56105	**ZH4-03**
snp_R05G6[4]	7507574	7000	
snp_R05G6[6]	7517162	9588	
uCE4-991	7535964	18802	
uCE4-993	7553246	17282	
snp_F45E4[2]	7627927	74681	
snp_F45E4[3]	7628008	81	
snp_F45E4[6]	7642093	14085	
snp_C08G9[1]	7676588	34495	
pkP664	8029872	353284	
**snp_C18F3[1]**	8058041	28169	**ZH4-17**

pkP672	8150283	92242	
uCE4-1026	8270504	120221	
snp_K07H8[1]	8271492	988	
uCE4-1048	8419362	147870	
pkP512	8534818	115456	
uCE4-1057	8560880	26062	
snp_F42A9[1]	8609542	48662	
**snp_F49C12[1]**	9310637	701095	**ZH4-18**
pkP5000	9685240	374603	
pkP739	9686848	1608	
pkP680	9686851	3	
pkP703	9686941	90	
pkP713	9686951	10	
pkP711	9686956	5	
uCE4-1082	9715775	28819	
snp_T13F2[3]	9788641	72866	
**pkP4081**	9834093	45452	**ZH4-09**
pkP5151	9870910	36817	
snp_T13H10[1]	9946155	75245	
pkP614	9993362	47207	
uCE4-1091	10112206	118844	
uCE4-1092	10112498	292	
uCE4-1103	10270079	157581	
pkP528	10308879	38800	
pkP5253	10429429	120550	
pkP576	10429431	2	
uCE4-1112	10514023	84592	
pkP5200	10539948	25925	
pkP5034	10539955	7	
snp_ZC168[1]	10739893	199938	
snp_C06G8[1]	10791657	51764	
uCE4-1119	10815135	23478	
snp_T11G6[1]	10862089	46954	
snp_ZK596[1]	10901386	39297	
**snp_C08F8[2]**	11172762	271376	**ZH4-19**
snp_F54D1[2]	11282581	109819	
snp_B0035[2]	11327189	44608	
snp_T01G1[4]	11359153	31964	
uCE4-1150	11640408	281255	
snp_T22B3[1]	11703149	62741	
pkP756	12056931	353782	
**snp_M18[1]**	12104785	47854	**ZH4-20**
uCE4-1172	12167213	62428	
uCE4-1174	12202476	35263	
snp_C42C1[4]	12283691	81215	
snp_C42C1[6]	12285694	2003	
uCE4-1178	12317174	31480	
snp_F19B6[4]	12347475	30301	
snp_F28D1[1]	12380518	33043	
uCE4-1182	12451709	71191	
pkP570	12515358	63649	
pkP5038	12515359	1	
uCE4-1191	12613146	97787	
snp_Y55D9[1]	12614006	860	
pkP5219	12721944	107938	
pkP5234	12722153	209	
pkP5027	12722282	129	
pkP5267	12734153	11871	
pkP5247	12734237	84	
snp_F07C6[1]	12795236	60999	
**snp_K08D8[2]**	12912468	117232	**ZH4-10a**
snp_F55G11[6]	12972376	59908	
uCE4-1224	12999769	27393	
uCE4-1230	13011281	11512	
uCE4-1233	13023025	11744	
snp_F09E8[1]	13160986	137961	
snp_C48D1[1]	13206181	45195	
snp_C48D1[4]	13214970	8789	
snp_C48D1[8]	13230590	15620	
snp_Y45F10[1]	13490851	260261	
pkP4090	13519932	29081	
snp_Y45F10B[3]	13564981	45049	
uCE4-1254	13612593	47612	
**snp_C08F11[4]**	13629499	16906	**ZH4-21**
snp_T23G4[1]	13698299	68800	
uCE4-1262	13730633	32334	
snp_Y45F10D[7]	13810104	79471	
snp_Y45F10D[8]	13810970	866	
uCE4-1265	13904267	93297	
uCE4-1268	13905498	1231	
snp_C27H2[2]	13952818	47320	
snp_Y37A1B[1]	13987300	34482	
uCE4-1270	13996132	8832	
snp_Y37A1B[4]	14009549	13417	
pkP767	14035502	25953	
pkP868	14035508	6	
pkP5300	14035520	12	
snp_Y37A1B[8]	14035883	363	
snp_Y37A1B[12]	14059204	23321	
snp_Y37A1B[13]	14059204	0	
snp_Y37A1B[14]	14059204	0	

uCE4-1280	14073746	14542	
snp_F52B11[3]	14102677	28931	
uCE4-1292	14164515	61838	
uCE4-1304	14229002	64487	
**pkP4094**	14245143	16141	**ZH4-11**
uCE4-1308	14263631	18488	
uCE4-1310	14263846	215	
snp_Y67A10[2]	14318830	54984	
uCE4-1319	14333138	14308	
snp_Y67A10[8]	14380625	47487	
uCE4-1331	14419971	39346	
snp_LLC1[1]	14435305	15334	
pkP5033	14602777	167472	
snp_F13G11[5]	14620217	17440	
snp_F13G11[6]	14620595	378	
snp_Y57G11C[6]	14727772	107177	
uCE4-1372	14766349	38577	
uCE4-1375	14772173	5824	
snp_Y57G11C[13]	14808789	36616	
snp_Y57G11C[18]	14826069	17280	
uCE4-1386	14859268	33199	
uCE4-1387	14859402	134	
snp_Y57G11C[19]	14869805	10403	
uCE4-1393	14893497	23692	
uCE4-1400	14936427	42930	
snp_Y41E3[1]	14981935	45508	
snp_Y41E3[10]	15004166	22231	
uCE4-1416	15014468	10302	
uCE4-1417	15014477	9	
snp_M199[2]	15104639	90162	
uCE4-1427	15141706	37067	
**snp_Y40H7[3]**	15170907	29201	**ZH4-12**
snp_Y40H7[5]	15171274	367	
snp_Y40H7[11]	15219122	47848	
snp_Y73F8[5]	15244035	24913	
uCE4-1428	15244649	614	
snp_Y73F8[14]	15274926	30277	
snp_Y73F8[15]	15274952	26	
snp_Y73F8[16]	15275133	181	
snp_Y73F8[17]	15275305	172	
snp_Y73F8[25]	15321464	46159	
snp_Y73F8[28]	15338476	17012	
snp_Y73F8[32]	15356918	18442	
snp_Y73F8[33]	15357035	117	
uCE4-1463	15522220	165185	
snp_Y105C5[16]	15851084	328864	
snp_Y105C5B[19]	15937613	86529	
uCE4-1505	15964434	26821	
uCE4-1507	15965319	885	
uCE4-1508	15965561	242	
snp_Y105C5B[21]	15967980	2419	
**snp_Y105C5B[23]**	15968421	441	**ZH4-22**
uCE4-1514	15980747	12326	
pkP581	15982070	1323	
pkP972	16005704	23634	
pkP5252	16005757	53	
uCE4-1521	16006530	773	
snp_Y105C5B[36]	16087457	80927	
pkP5326	16092200	4743	
snp_Y105C5B[45]	16121111	28911	
pkP636	16153204	32093	
snp_Y105C5B[50]	16155149	1945	
snp_Y7A9[3]	16189190	34041	
snp_Y7A9[5]	16189483	293	
snp_H25K10[2]	16212235	22752	
uCE4-1549	16278066	65831	
pkP562	16332240	54174	
pkP545	16336676	4436	
pkP5051	16336679	3	
snp_C35D6[2]	16371727	35048	
uCE4-1588	16443913	72186	
uCE4-1590	16502465	58552	
snp_Y51H4[8]	16596019	93554	
snp_Y51H4[12]	16619897	23878	
uCE4-1599	16623593	3696	
snp_Y51H4[21]	16679631	56038	
snp_Y51H4[22]	16679759	128	
snp_Y116A8[1]	16810742	130983	
snp_Y116A8[4]	16811029	287	
snp_T06A10[7]	16876994	65965	
uCE4-1615	16890397	13403	
snp_Y116A8C[2]	16950141	59744	
snp_Y116A8C[5]	17084189	134048	
snp_Y116A8C[9]	17084439	250	
uCE4-1626	17161097	76658	
pkP5306	17197490	36393	
uCE4-1631	17217269	19779	
uCE4-1633	17235347	18078	
uCE4-1634	17235739	392	
uCE4-1638	17285773	50034	
pkP923	17306109	20336	

pkP5102	17322047	15938
pkP696	17322051	4
pkP530	17345839	23788
pkP5082	17460653	114814
