# Supplementary material for: A universal method for automated gene mapping
Source: Genome Biol. 2005 Jan 17;6(2):R19. doi: 10.1186/gb-2005-6-2-r19 (PMC551539; doi:10.1186/gb-2005-6-2-r19)
Supplement: Additional data file 16 — Additional non-validated FLPs (predicted C. elegans InDels LGV) [file gb-2005-6-2-r19-s16.pdf]

Supplementary Table 8:
Predicted *C. elegans* InDels LGV

(Validated FLP assays are shown in blue)

WormBase SNP	Position on Chromosome (nt)	Distance between InDels (nt)	Assay Name
snp_B0348[3]	39766	-	
snp_B0348[5]	39828	62	
uCE5-503	78769	38941	
snp_W03F9[2]	144688	65919	
snp_W03F9[3]	153521	8833	
uCE5-507	154108	587	
uCE5-508	155951	1843	
snp_DC2[6]	230339	74388	
snp_T22H9[1]	331855	101516	
uCE5-533	535089	203234	
snp_C14C6[1]	555066	19977	
uCE5-588	593015	37949	
pkP750	665421	72406	
pkP5193	665424	3	
pkP747	670119	4695	
pkP5012	670125	6	
snp_H24O09[2]	724097	53972	
**snp_C05E4[5]**	749319	25222	**ZH5-02a**
snp_R09E12[3]	779255	29936	
snp_R09E12[2]	779256	1	
uCE5-653	819507	40251	
snp_T02B11[6]	876277	56770	
uCE5-665	913476	37199	
uCE5-671	937584	24108	
snp_Y50D4C[3]	955887	18303	
snp_R09A1[4]	1013499	57612	
snp_R09A1[8]	1025990	12491	
snp_R09A1[10]	1039193	13203	
snp_R09A1[11]	1039197	4	
uCE5-680	1076959	37762	
uCE5-697	1124481	47522	
uCE5-710	1303680	179199	
uCE5-715	1397321	93641	
snp_R12A1[2]	1402692	5371	
snp_R09B5[2]	1459116	56424	
uCE5-722	1508320	49204	
**pkP5101**	1551061	42741	**ZH5-13**
snp_F52F10[2]	1551302	241	
snp_F16B4[1]	1618295	66993	
uCE5-735	1679028	60733	
pkP5091	1689974	10946	
snp_Y46H3C[1]	1690109	135	
uCE5-739	1697964	7855	
snp_Y46H3C[4]	1706595	8631	
snp_Y46H3C[5]	1706628	33	
snp_Y46H3C[7]	1706963	335	
uCE5-753	1767229	60266	
snp_F36H9[1]	1768262	1033	
snp_F36H9[2]	1768534	272	
uCE5-757	1796652	28118	
snp_Y32G9[2]	1912729	116077	
snp_T06A1[2]	1937151	24422	
uCE5-763	1938040	889	
snp_F59A7[7]	2021412	83372	
uCE5-771	2074120	52708	
uCE5-774	2075553	1433	
**snp_F41B5[5]**	2239502	163949	**ZH5-03a**
snp_F41B5[6]	2239573	71	
pkP5157	2322267	82694	
snp_W02G9[3]	2682725	360458	
pkP657	2708519	25794	
uCE5-824	2717694	9175	
snp_R05D8[2]	2767703	50009	
snp_R05D8[3]	2767937	234	
snp_R05D8[6]	2767977	40	
snp_R05D8[10]	2775914	7937	
snp_F54E2[2]	2795896	19982	
snp_F54E2[5]	2799376	3480	
snp_H05B21[3]	2952069	152693	
snp_C44C3[3]	2975486	23417	
uCE5-881	3022472	46986	
snp_F59D6[3]	3028371	5899	
uCE5-887	3055134	26763	
uCE5-894	3058341	3207	
snp_C50H11[4]	3064879	6538	
pkP730	3087297	22418	
uCE5-908	3123727	36430	
pkP722	3206067	82340	
uCE5-928	3216396	10329	
snp_K08D9[8]	3218704	2308	
snp_K08D9[11]	3220084	1380	
snp_K08D9[12]	3220111	27	

snp_K08D9[13]	3220193	82	
snp_K08D9[14]	3220219	26	
snp_K08D9[15]	3220290	71	
snp_K08D9[20]	3223074	2784	
pkP5018	3252761	29687	
uCE5-937	3308461	55700	
uCE5-962	3443558	135097	
uCE5-980	3443978	420	
uCE5-1008	3471791	27813	
uCE5-1013	3529499	57708	
uCE5-1015	3541353	11854	
snp_T24A6[5]	3545339	3986	
pkP5131	3590061	44722	
pkP539	3591672	1611	
snp_T22F3[2]	3606322	14650	
uCE5-1040	3739280	132958	
uCE5-1046	3751527	12247	
pkP5198	3753102	1575	
pkP642	3768908	15806	
uCE5-1052	3770238	1330	
snp_R08E5[9]	3771515	1277	
uCE5-1063	3793994	22479	
uCE5-1068	3819886	25892	
snp_H10D18[9]	3835113	15227	
**snp_F47C10[2]**	3848148	13035	**ZH5-14**
uCE5-1072	3883369	35221	
uCE5-1098	3908074	24705	
uCE5-1155	3951337	43263	
uCE5-1162	3951513	176	
snp_B0213[3]	3961484	9971	
pkP5094	3989614	28130	
pkP675	3991270	1656	
snp_Y45G5AL[1]	4139631	148361	
uCE5-1197	4296565	156934	
snp_ZC132[2]	4305585	9020	
uCE5-1222	4366519	60934	
snp_F13A2[4]	4381581	15062	
snp_T28F12[3]	4529970	148389	
snp_F41F3[1]	4648904	118934	
pkP686	4936413	287509	
snp_C13D9[1]	4988672	52259	
snp_C13D9[4]	4988787	115	
snp_C13D9[5]	4988794	7	
snp_C13D9[6]	4988813	19	
snp_C13D9[10]	4996433	7620	
uCE5-1253	5032933	36500	
uCE5-1254	5032983	50	
uCE5-1258	5038382	5399	
snp_C04F5[1]	5091141	52759	
uCE5-1312	5173459	82318	
**snp_C03A7[4]**	5181248	7789	**ZH5-04**
snp_C03A7[5]	5181589	341	
snp_C03A7[8]	5185473	3884	
snp_C03A7[9]	5185476	3	
snp_C03A7[10]	5185477	1	
snp_C03A7[11]	5185486	9	
snp_C03A7[13]	5185601	115	
snp_C03A7[12]	5185655	54	
snp_C03A7[7]	5185655	0	
snp_Y49G5B[2]	5221006	35351	
uCE5-1319	5244287	23281	
uCE5-1352	5323926	79639	
pkP582	5328362	4436	
pkP5270	5360639	32277	
pkP612	5360642	3	
pkP922	5425863	65221	
pkP5223	5426077	214	
uCE5-1362	5462246	36169	
pkP584	5481775	19529	
snp_C18C4[1]	5547982	66207	
snp_C18C4[2]	5547990	8	
**snp_C18C4[3]**	5548085	95	**ZH5-15**
snp_C18C4[5]	5548827	742	
uCE5-1367	5551176	2349	
uCE5-1372	5652976	101800	
uCE5-1406	5892541	239565	
uCE5-1410	5898418	5877	
pkP690	5933366	34948	
snp_M03F8[1]	5953434	20068	
snp_F29G9[5]	6031356	77922	
uCE5-1417	6179885	148529	
uCE5-1420	6180358	473	
uCE5-1421	6180397	39	
uCE5-1424	6183713	3316	
uCE5-1425	6183724	11	
snp_W06H8[1]	6223188	39464	
uCE5-1430	6273712	50524	
snp_K12B6[3]	6302651	28939	
snp_T28C12[6]	6342374	39723	
snp_T28C12[7]	6342382	8	
pkP692	6350881	8499	

snp_F13H6[5]	6354308	3427	
snp_F13H6[6]	6370939	16631	
uCE5-1445	6420042	49103	
uCE5-1453	6431615	11573	
uCE5-1456	6436906	5291	
uCE5-1457	6439374	2468	
uCE5-1460	6439818	444	
pkP5008	6473164	33346	
uCE5-1461	6480265	7101	
**snp_K04A8[2]**	6565411	85146	**ZH5-05**
uCE5-1466	6620138	54727	
uCE5-1470	6635332	15194	
uCE5-1473	6657054	21722	
snp_T10H9[3]	6661584	4530	
snp_W02F12[1]	6718735	57151	
snp_T25F10[1]	6761505	42770	
snp_T25F10[3]	6772345	10840	
uCE5-1495	6816823	44478	
snp_T19F4[1]	6879367	62544	
snp_T19F4[2]	6881484	2117	
uCE5-1502	6881485	1	
pkP585	7010894	129409	
pkP5401	7010948	54	
uCE5-1514	7038699	27751	
snp_F20A1[5]	7043272	4573	
pkP5293	7160388	117116	
snp_F09G2[1]	7169656	9268	
snp_C13F10[2]	7225682	56026	
snp_C13F10[5]	7226361	679	
snp_C05C8[2]	7240104	13743	
uCE5-1522	7263976	23872	
uCE5-1529	7286344	22368	
uCE5-1530	7286356	12	
pkP5249	7340386	54030	
pkP740	7365383	24997	
pkP5317	7377099	11716	
uCE5-1537	7411583	34484	
uCE5-1541	7449691	38108	
snp_F25E5[1]	7453169	3478	
snp_C54F6[2]	7521912	68743	
snp_C50E3[2]	7594591	72679	
uCE5-1556	7603763	9172	
uCE5-1592	7624405	20642	
uCE5-1648	7656519	32114	
uCE5-1653	7656680	161	
uCE5-1662	7661970	5290	
uCE5-1688	7684405	22435	
snp_Y97E10C[1]	7731041	46636	
uCE5-1694	7736324	5283	
**snp_ZK742[1]**	7801937	65613	**ZH5-16**
pkP5132	7847176	45239	
uCE5-1713	7868675	21499	
uCE5-1718	7986563	117888	
snp_Y97E10AL[1]	7988656	2093	
snp_C09H5[1]	8069166	80510	
pkP5056	8104819	35653	
uCE5-1720	8236206	131387	
snp_F21F8[1]	8269605	33399	
snp_T23B12[2]	8483713	214108	
pkP593	8670248	186535	
pkP870	8670258	10	
pkP5248	8740650	70392	
pkP5147	8877135	136485	
snp_H14N18[1]	8927900	50765	
pkP734	8997002	69102	
snp_C25E10[2]	9045651	48649	
pkP501	9072456	26805	
pkP745	9127999	55543	
pkP5010	9128103	104	
pkP520	9128106	3	
uCE5-1738	9199370	71264	
uCE5-1741	9249992	50622	
snp_E02C12[3]	9352572	102580	
uCE5-1748	9372618	20046	
**snp_E02C12[8]**	9387102	14484	**ZH5-01**
snp_F36D4[4]	9419038	31936	
uCE5-1779	9455946	36908	
snp_K06A4[1]	9487103	31157	
snp_F25B3[2]	9570870	83767	
uCE5-1781	9601176	30306	
snp_ZK287[1]	9677467	76291	
uCE5-1789	9720975	43508	
pkP5168	9834822	113847	
pkP5197	9892918	58096	
pkP741	9893826	908	
pkP714	9893834	8	
pkP5309	9954706	60872	
uCE5-1798	9966416	11710	
**snp_K08H10[1]**	10008679	42263	**ZH5-17**
snp_R04B5[1]	10096507	87828	
pkP710	10106195	9688	

uCE5-1804	10134515	28320	
snp_C51E3[1]	10170006	35491	
pkP5145	10191846	21840	
pkP660	10267623	75777	
uCE5-1814	10395768	128145	
snp_F15H10[1]	10422285	26517	
pkP5175	10496954	74669	
uCE5-1821	10572602	75648	
snp_F32D8[1]	10877221	304619	
uCE5-1852	10997528	120307	
pkP712	11087202	89674	
pkP5130	11087205	3	
snp_C06H2[3]	11137526	50321	
snp_C06H2[4]	11142531	5005	
uCE5-1864	11276550	134019	
uCE5-1865	11276550	0	
uCE5-1866	11276550	0	
uCE5-1867	11276550	0	
**snp_C03E10[1]**	11284131	7581	**ZH5-18**
pkP523	11292879	8748	
pkP575	11383772	90893	
uCE5-1873	11408889	25117	
uCE5-1877	11438797	29908	
snp_T11F9[2]	11494313	55516	
pkP534	11497012	2699	
pkP554	11541894	44882	
snp_F46F3[2]	11685199	143305	
snp_D2023[5]	11808836	123637	
**snp_D2023[10]**	11830176	21340	**ZH5-06**
uCE5-1903	11919023	88847	
snp_F58H1[3]	11948344	29321	
uCE5-1906	11990890	42546	
uCE5-1909	12018370	27480	
pkP5128	12105561	87191	
pkP640	12105561	0	
pkP5181	12284852	179291	
pkP587	12310321	25469	
snp_R04F11[1]	12316739	6418	
snp_R04F11[4]	12333339	16600	
snp_F23H12[2]	12349532	16193	
pkP5069	12378457	28925	
uCE5-1974	12463739	85282	
uCE5-2428	12494424	30685	
uCE5-2429	12494431	7	
snp_T28H10[1]	12509909	15478	
uCE5-1977	12537417	27508	
snp_F45D3[7]	12556903	19486	
snp_E02A10[1]	12585452	28549	
snp_E02A10[3]	12588744	3292	
uCE5-1986	12642740	53996	
snp_W05B10[1]	12678956	36216	
snp_W05B10[3]	12680840	1884	
uCE5-1988	12714754	33914	
uCE5-1991	12748427	33673	
uCE5-1999	12818311	69884	
uCE5-2001	12957628	139317	
uCE5-2004	13007413	49785	
snp_F42E8[2]	13069662	62249	
uCE5-2005	13073288	3626	
uCE5-2006	13073453	165	
snp_B0365[1]	13133316	59863	
snp_K03H4[1]	13164123	30807	
uCE5-2016	13208583	44460	
**snp_C50B6[6]**	13342985	134402	**ZH5-11**
snp_C50B6[8]	13342990	5	
uCE5-2025	13417124	74134	
snp_C56A3[1]	13535151	118027	
pkP667	13535845	694	
uCE5-2032	13663650	127805	
snp_F35E12[6]	13750745	87095	
snp_F55B12[3]	13834089	83344	
uCE5-2043	13918546	84457	
pkP655	13927722	9176	
pkP5156	13927724	2	
pkP716	13927746	22	
pkP945	13939296	11550	
uCE5-2057	13978777	39481	
snp_T26F2[2]	13981104	2327	
snp_F58E10[2]	13991647	10543	
pkP623	14025725	34078	
pkP5068	14025728	3	
snp_D1086[3]	14090740	65012	
snp_AH10[4]	14152932	62192	
**snp_T16A9[2]**	14220019	67087	**ZH5-23**
uCE5-2078	14352343	132324	
snp_F23B12[1]	14448361	96018	
snp_F43D2[1]	14628078	179717	
snp_F43D2[2]	14628080	2	
snp_F43D2[3]	14628292	212	
snp_C54G10[2]	14654108	25816	
pkP5114	14758017	103909	

snp_C48G7[2]	14806785	48768	
snp_H12D21[3]	14910964	104179	
pkP717	14929322	18358	
pkP695	14929353	31	
pkP725	14929444	91	
pkP5124	14929445	1	
snp_C30G7[1]	14942686	13241	
snp_C30G7[3]	14953111	10425	
snp_F02D8[1]	14986682	33571	
pkP665	15089817	103135	
pkP626	15130019	40202	
uCE5-2134	15331678	201659	
pkP574	15338639	6961	
uCE5-2135	15351523	12884	
snp_R08H2[1]	15376669	25146	
pkP693	15414245	37576	
pkP5186	15415400	1155	
pkP978	15416909	1509	
**snp_Y36E3[4]**	15430591	13682	**ZH5-12**
uCE5-2140	15446976	16385	
uCE5-2148	15576766	129790	
snp_B0391[1]	15615224	38458	
snp_B0391[3]	15615941	717	
snp_B0391[5]	15615944	3	
snp_C55A1[1]	15630697	14753	
uCE5-2160	15656602	25905	
snp_K06B4[1]	15677064	20462	
snp_K06B4[4]	15677234	170	
uCE5-2203	15792108	114874	
uCE5-2247	15966690	174582	
uCE5-2271	15976310	9620	
uCE5-2277	15978060	1750	
uCE5-2281	15978127	67	
pkP842	16074809	96682	
pkP802	16074812	3	
pkP793	16074814	2	
uCE5-2302	16076882	2068	
**snp_F22B8[2]**	16101559	24677	**ZH5-20**
snp_F44G3[3]	16123288	21729	
snp_F44G3[6]	16123559	271	
uCE5-2309	16130658	7099	
uCE5-2328	16254671	124013	
snp_T13F3[1]	16267497	12826	
snp_T13F3[2]	16267500	3	
pkP5043	16276096	8596	
uCE5-2356	16351993	75897	
uCE5-2357	16352058	65	
uCE5-2363	16382411	30353	
uCE5-2364	16388002	5591	
uCE5-2370	16410248	22246	
uCE5-2373	16410424	176	
snp_T08G3[1]	16446288	35864	
uCE5-2388	16452798	6510	
uCE5-2391	16452800	2	
uCE5-2392	16452843	43	
uCE5-2386	16452858	15	
uCE5-2389	16452858	0	
uCE5-2387	16452864	6	
uCE5-2390	16452864	0	
uCE5-2394	16453140	276	
uCE5-2399	16462744	9604	
pkP787	16516882	54138	
pkP578	16516897	15	
snp_T03E6[3]	16580776	63879	
uCE5-2418	16598003	17227	
snp_F14D1[1]	16762502	164499	
snp_F14D1[2]	16762836	334	
pkP5327	16777749	14913	
uCE5-2455	16779768	2019	
uCE5-2456	16780696	928	
snp_Y102A5[1]	16803990	23294	
**snp_T20B3[1]**	16847598	43608	**ZH5-08**
uCE5-2506	17084394	236796	
uCE5-2517	17107582	23188	
uCE5-2518	17107637	55	
uCE5-2532	17131527	23890	
snp_F40D4[4]	17168246	36719	
snp_F40D4[6]	17177795	9549	
snp_Y20C6[3]	17377777	199982	
uCE5-2557	17511542	133765	
snp_C38D9[4]	17589353	77811	
uCE5-2570	17604175	14822	
uCE5-2581	17608470	4295	
uCE5-2611	17610603	2133	
uCE5-2613	17612354	1751	
uCE5-2624	17660103	47749	
snp_F59A1[6]	17679825	19722	
snp_Y26G10[1]	17703409	23584	
uCE5-2716	17786372	82963	
snp_C47A10[2]	17795399	9027	
snp_Y59A8[1]	17844934	49535	

pkP771	17917836	72902	
uCE5-2738	17936240	18404	
snp_Y59A8B[1]	17937893	1653	
uCE5-2740	17965548	27655	
uCE5-2741	17965568	20	
uCE5-2743	17991769	26201	
uCE5-2759	18012203	20434	
pkP622	18018499	6296	
pkP5057	18018501	2	
snp_Y59A8B[8]	18036305	17804	
uCE5-2763	18048139	11834	
snp_Y37H2[2]	18114425	66286	
**snp_C14A6[1]**	18147681	33256	**ZH5-21**
snp_C14A6[3]	18147694	13	
snp_C14A6[4]	18147702	8	
pkP5166	18186531	38829	
pkP5140	18186538	7	
snp_R10E8[2]	18260925	74387	
uCE5-2791	18262897	1972	
uCE5-2805	18358390	95493	
snp_C08E8[15]	18370766	12376	
snp_Y51A2B[2]	18394937	24171	
pkP5048	18446528	51591	
snp_Y51A2D[3]	18495698	49170	
uCE5-2839	18502665	6967	
uCE5-2842	18510340	7675	
snp_Y51A2D[9]	18517399	7059	
snp_Y51A2D[11]	18517605	206	
pkP5227	18517648	43	
snp_Y51A2D[14]	18586589	68941	
snp_Y51A2D[15]	18586617	28	
snp_Y51A2D[18]	18600448	13831	
snp_Y51A2D[20]	18600652	204	
uCE5-2848	18640526	39874	
pkP586	18711374	70848	
pkP5271	18711378	4	
snp_Y17D7C[1]	18728047	16669	
snp_Y17D7C[2]	18728083	36	
uCE5-2860	18770746	42663	
snp_F11D11[9]	18770755	9	
snp_Y17D7B[5]	18788986	18231	
snp_C54E10[3]	18818755	29769	
snp_Y80D3[9]	18939716	120961	
uCE5-2884	18940715	999	
snp_Y39B6[5]	18996702	55987	
snp_Y39B6[6]	18996877	175	
snp_Y39B6[7]	19002448	5571	
snp_Y39B6[10]	19003879	1431	
uCE5-2888	19130057	126178	
**snp_Y39B6[17]**	19147608	17551	**ZH5-09**
snp_Y39B6[19]	19186825	39217	
snp_Y39B6[8]	19202968	16143	
uCE5-2905	19250571	47603	
pkP527	19256763	6192	
uCE5-2914	19290269	33506	
uCE5-2986	19380884	90615	
uCE5-3006	19516859	135975	
uCE5-3024	19536057	19198	
snp_Y43F8B[3]	19537522	1465	
snp_Y43F8B[4]	19541515	3993	
snp_B0399[8]	19586726	45211	
uCE5-3036	19611710	24984	
snp_Y43F8C[8]	19636935	25225	
snp_Y43F8C[9]	19637014	79	
snp_Y43F8C[11]	19645976	8962	
snp_Y116F11[5]	19724404	78428	
uCE5-3052	19748957	24553	
uCE5-3057	19751392	2435	
uCE5-3058	19751396	4	
uCE5-3059	19751397	1	
uCE5-3060	19751416	19	
uCE5-3061	19751421	5	
uCE5-3062	19751428	7	
uCE5-3063	19751438	10	
uCE5-3066	19751465	27	
uCE5-3067	19751472	7	
uCE5-3073	19751474	2	
uCE5-3076	19751475	1	
uCE5-3082	19751475	0	
uCE5-3077	19751479	4	
uCE5-3080	19751482	3	
uCE5-3068	19751488	6	
uCE5-3069	19751489	1	
uCE5-3074	19751490	1	
uCE5-3078	19751490	0	
uCE5-3081	19751490	0	
uCE5-3075	19751491	1	
uCE5-3079	19751491	0	
uCE5-3088	19786674	35183	
uCE5-3092	19820134	33460	
pkP858	19964309	144175	

pkP819	19964310	1	
pkP556	19964314	4	
snp_Y60A3[1]	20020826	56512	
snp_Y60A3[2]	20023186	2360	
pkP648	20040000	16814	
snp_Y113G7[2]	20045469	5469	
snp_Y113G7[4]	20045585	116	
**snp_Y113G7[6]**	20050070	4485	**ZH5-22**
snp_Y113G7[8]	20050074	4	
uCE5-3104	20060012	9938	
snp_Y113G7[21]	20109570	49558	
pkP5046	20151835	42265	
snp_ZC15[1]	20274858	123023	
uCE5-3178	20416286	141428	
uCE5-3181	20437794	21508	
uCE5-3184	20437980	186	
uCE5-3185	20437986	6	
snp_F48F5[6]	20445932	7946	
pkP5105	20461946	16014	
snp_B0250[2]	20465938	3992	
snp_B0250[4]	20466304	366	
uCE5-3212	20504110	37806	
uCE5-3221	20504545	435	
uCE5-3251	20524051	19506	
snp_Y38H6C[6]	20534624	10573	
pkP969	20548576	13952	
pkP5074	20548579	3	
uCE5-3352	20603039	54460	
uCE5-3388	20616740	13701	
uCE5-3394	20652628	35888	
uCE5-3396	20652967	339	
pkP5064	20654877	1910	
pkP591	20654881	4	
pkP942	20663914	9033	
pkP940	20663916	2	
snp_W01F3[5]	20678611	14695	
uCE5-3409	20763192	84581	
pkP5292	20787623	24431	
snp_Y44A6D[1]	20790963	3340	
snp_Y44A6D[5]	20804012	13049	
snp_F31D4[1]	20858860	54848	
uCE5-3418	20867753	8893	
snp_Y44A6E[2]	20917647	49894	
