# Supplementary material for: A universal method for automated gene mapping
Source: Genome Biol. 2005 Jan 17;6(2):R19. doi: 10.1186/gb-2005-6-2-r19 (PMC551539; doi:10.1186/gb-2005-6-2-r19)
Supplement: Additional data file 17 — Additional non-validated FLPs (predicted C. elegans InDels LGX) [file gb-2005-6-2-r19-s17.pdf]

Supplementary Table 9:
Predicted *C. elegans* InDels LGX

(Validated FLP assays are shown in blue)

WormBase SNP	Position on Chromosome (nt)	Distance between InDels (nt)	Assay Name
uCE6-521	52951	-	
uCE6-518	109323	56372	
snp_Y73B3[1]	147486	38163	
snp_Y73B3B[2]	206660	59174	
snp_AC8[1]	225149	18489	
pkP5196	228408	3259	
pkP519	228413	5	
uCE6-530	290009	61596	
uCE6-522	290011	2	
uCE6-525	290041	30	
uCE6-526	290049	8	
uCE6-527	290059	10	
uCE6-528	290062	3	
uCE6-529	290088	26	
uCE6-523	290196	108	
uCE6-538	406296	116100	
uCE6-539	423438	17142	
uCE6-542	518390	94952	
uCE6-546	639067	120677	
**snp_C02H7[1]**	734252	95185	**ZHX-16**
pkP5165	737138	2886	
uCE6-554	772881	35743	
uCE6-558	855896	83015	
uCE6-565	1001242	145346	
uCE6-568	1057956	56714	
**snp_C46H3[1]**	1210652	152696	**ZHX-17**
snp_C46H3[3]	1215467	4815	
uCE6-582	1270501	55034	
snp_C52B11[8]	1299981	29480	
uCE6-589	1395559	95578	
snp_F19G12[1]	1426106	30547	
uCE6-595	1447593	21487	
snp_F09E10[5]	1497222	49629	
uCE6-599	1500879	3657	
uCE6-638	1553838	52959	
uCE6-639	1553889	51	
uCE6-644	1659826	105937	
uCE6-687	1663737	3911	
uCE6-706	1850761	187024	
snp_T26C11[1]	1861200	10439	
snp_Y40A1[1]	1989912	128712	
**snp_F49H12[2]**	2090657	100745	**ZHX-03**
pkP670	2153697	63040	
uCE6-726	2215587	61890	
uCE6-729	2262902	47315	
snp_T24D8[7]	2355718	92816	
snp_T24D8[10]	2355958	240	
snp_F47G3[2]	2386372	30414	
uCE6-751	2391708	5336	
uCE6-760	2489084	97376	
snp_F52H2[1]	2548791	59707	
snp_T03G6[2]	2597063	48272	
pkP5308	2674707	77644	
snp_C09B7[2]	2677804	3097	
snp_T02C5[1]	2702342	24538	
snp_T02C5[2]	2708004	5662	
snp_T02C5[4]	2710066	2062	
snp_Y76F7[1]	2726891	16825	
snp_Y76F7[2]	2727139	248	
snp_F55F1[1]	2768649	41510	
snp_F55F1[5]	2773407	4758	
snp_C14A11[1]	2790266	16859	
snp_Y71H10B[2]	2841154	50888	
uCE6-779	2853880	12726	
uCE6-785	2926754	72874	
snp_Y41G9[1]	2951841	25087	
snp_Y41G9[2]	2957315	5474	
snp_Y41G9[3]	2962203	4888	
snp_Y41G9[4]	2970778	8575	
snp_Y41G9[5]	2971156	378	
pkP5143	3014898	43742	
pkP5020	3038425	23527	
uCE6-791	3041157	2732	
snp_C15C7[1]	3156486	115329	

**snp_C15C7[3]**	3157420	934	**ZHX-08**
snp_F28B4[2]	3219035	61615	
snp_F28B4[5]	3226627	7592	
snp_F11D5[3]	3323169	96542	
snp_ZK563[2]	3367877	44708	
snp_ZK563[4]	3376415	8538	
snp_ZK377[1]	3469424	93009	
snp_R11G1[1]	3635422	165998	
snp_R11G1[2]	3648848	13426	
snp_C01C4[1]	3697221	48373	
pkP744	3704905	7684	
uCE6-842	3936239	231334	
uCE6-843	3947206	10967	
uCE6-847	3994152	46946	
uCE6-849	3994292	140	
uCE6-850	3994301	9	
uCE6-853	4118478	124177	
snp_F09F9[1]	4129297	10819	
snp_C52B9[4]	4258507	129210	
uCE6-855	4308901	50394	
**snp_R160[2]**	4377207	68306	**ZHX-13**
snp_R160[3]	4377318	111	
snp_R160[5]	4401727	24409	
snp_R160[6]	4401747	20	
pkP733	4420207	18460	
pkP5256	4423336	3129	
pkP965	4433624	10288	
pkP5229	4433640	16	
uCE6-866	4507557	73917	
snp_F46H6[7]	4513955	6398	
uCE6-869	4561518	47563	
snp_F55D10[3]	4719079	157561	
pkP538	4779731	60652	
**pkP6106**	4892210	112479	**ZHX-15**
pkP526	4958360	66150	
pkP754	5083101	124741	
snp_C31H2[4]	5127411	44310	
snp_T03G11[3]	5179664	52253	
uCE6-903	5234576	54912	
uCE6-927	5390191	155615	
snp_C25F6[2]	5466279	76088	
pkP743	5489327	23048	
snp_T23F2[3]	5493904	4577	
uCE6-931	5508057	14153	
uCE6-933	5517030	8973	
snp_C38C5[1]	5562465	45435	
snp_C38C5[3]	5562888	423	
snp_F14D12[1]	5614980	52092	
snp_W01C8[1]	5675135	60155	
uCE6-947	5718174	43039	
snp_C41G11[2]	5729842	11668	
snp_C54H2[4]	5770133	40291	
snp_F46G11[2]	5798689	28556	
snp_F46G11[3]	5798706	17	
snp_F13D11[4]	5818213	19507	
uCE6-959	5819246	1033	
uCE6-966	5877817	58571	
pkP748	5896619	18802	
pkP5006	5896637	18	
pkP511	5896640	3	
snp_Y23B4[3]	5925075	28435	
snp_F22F4[1]	5982704	57629	
pkP943	6025313	42609	
uCE6-986	6047651	22338	
snp_C14F11[1]	6244196	196545	
uCE6-989	6296346	52150	
snp_K10C2[2]	6427081	130735	
**snp_K04E7[1]**	6455366	28285	**ZHX-10**
pkP5141	6462915	7549	
snp_T14E8[1]	6543476	80561	
pkP5058	6618074	74598	
snp_C56E10[2]	6654207	36133	
uCE6-999	6693564	39357	
snp_C53B7[2]	6863507	169943	
snp_F14B8[1]	6921666	58159	
pkP981	7017186	95520	
pkP5266	7018796	1610	
snp_C36B7[1]	7106845	88049	
snp_C36B7[4]	7108680	1835	
snp_C36B7[6]	7117968	9288	
snp_C55B6[1]	7183063	65095	
snp_F46H5[1]	7243181	60118	
snp_K03A1[1]	7300263	57082	

pkP618	7302951	2688	
snp_T08A9[1]	7315626	12675	
uCE6-1019	7319689	4063	
snp_C07D8[6]	7343858	24169	
snp_C10A4[1]	7403284	59426	
snp_K08A8[1]	7471843	68559	
uCE6-1036	7477323	5480	
snp_F46C8[2]	7554513	77190	
snp_F08C6[2]	7585682	31169	
snp_F26A10[2]	7615017	29335	
uCE6-1043	7656761	41744	
uCE6-1046	7785251	128490	
snp_R03G5[2]	7815759	30508	
snp_R03G5[3]	7820885	5126	
**snp_C54D2[3]**	7832035	11150	**ZHX-02**
pkP6109	7832756	721	
uCE6-1070	8002239	169483	
snp_C34D10[1]	8010910	8671	
pkP5014	8038033	27123	
pkP532	8038036	3	
uCE6-1077	8085855	47819	
pkP5244	8195727	109872	
pkP5023	8195728	1	
pkP5205	8195868	140	
snp_C47D2[1]	8202329	6461	
snp_C47D2[2]	8202592	263	
pkP5116	8212027	9435	
pkP5285	8318770	106743	
uCE6-1084	8381208	62438	
snp_F41D9[2]	8388918	7710	
**snp_F41D9[4]**	8391581	2663	**ZHX-24**
snp_T01B10[1]	8487006	95425	
snp_C03G5[1]	8533743	46737	
uCE6-1090	8606668	72925	
pkP960	8612805	6137	
pkP5037	8656467	43662	
snp_C28G1[1]	8844302	187835	
snp_T25B6[1]	9016277	171975	
pkP588	9057191	40914	
snp_C25A11[1]	9102704	45513	
uCE6-1110	9143677	40973	
snp_Y81B9[2]	9191677	48000	
snp_Y81B9[3]	9191814	137	
pkP704	9247148	55334	
snp_C23F12[1]	9410842	163694	
pkP709	9620043	209201	
snp_F15G9[2]	9725707	105664	
snp_F47A4[1]	9839716	114009	
pkP643	9861856	22140	
pkP5126	9955059	93203	
**pkP6138**	10010095	55036	**ZHX-07**
pkP976	10063916	53821	
snp_ZC373[3]	10078805	14889	
uCE6-1146	10195759	116954	
snp_C32A9[1]	10252595	56836	
snp_F41E7[7]	10296881	44286	
uCE6-1155	10316960	20079	
snp_R07E3[2]	10321628	4668	
snp_R07E3[4]	10321780	152	
uCE6-1159	10376728	54948	
snp_F46F6[3]	10381676	4948	
snp_F46F6[4]	10381747	71	
snp_F46F6[5]	10381760	13	
snp_F46F6[6]	10381902	142	
pkP503	10423230	41328	
pkP5127	10423230	0	
pkP720	10549450	126220	
uCE6-1179	10550866	1416	
snp_F59F5[2]	10557741	6875	
uCE6-1183	10662416	104675	
snp_T01C1[1]	10714489	52073	
snp_T01C1[7]	10731601	17112	
uCE6-1191	10761820	30219	
snp_T21B6[3]	10944495	182675	
snp_W04G3[2]	11076852	132357	
snp_F42E11[1]	11366985	290133	
snp_T25C12[1]	11461829	94844	
snp_T25C12[2]	11462023	194	
snp_T25C12[3]	11462036	13	
**snp_T25C12[4]**	11464550	2514	**ZHX-12**
snp_T25C12[8]	11491018	26468	
uCE6-1222	11492871	1853	
uCE6-1225	11564034	71163	

snp_T04F8[2]	11679277	115243	
uCE6-1229	11689186	9909	
uCE6-1231	11725660	36474	
uCE6-1232	11772907	47247	
uCE6-1236	11931639	158732	
uCE6-1242	12018335	86696	
uCE6-1251	12174612	156277	
pkP535	12194191	19579	
**snp_F17E5[1]**	12401713	207522	**ZHX-11**
snp_F17E5[4]	12407316	5603	
uCE6-1263	12416833	9517	
snp_C16D6[1]	12514519	97686	
snp_T24D5[1]	12568875	54356	
uCE6-1291	12615593	46718	
pkP5028	12628490	12897	
uCE6-1293	12641281	12791	
uCE6-1295	12642764	1483	
uCE6-1299	12812591	169827	
pkP5016	12815870	3279	
pkP757	12815872	2	
uCE6-1307	12920089	104217	
snp_C05E7[1]	12962243	42154	
pkP514	12996610	34367	
snp_R03G8[1]	13089113	92503	
uCE6-1323	13219586	130473	
snp_K08H2[1]	13228266	8680	
pkP980	13281083	52817	
pkP649	13284155	3072	
snp_R04D3[4]	13288884	4729	
**snp_R04D3[5]**	13288921	37	**ZHX-05**
snp_C49F8[4]	13371165	82244	
snp_F02C12[2]	13389241	18076	
uCE6-1346	13481140	91899	
snp_F54B11[1]	13583583	102443	
uCE6-1352	13624249	40666	
snp_C40C9[1]	13633955	9706	
snp_C40C9[2]	13633981	26	
uCE6-1357	13656725	22744	
pkP751	13665212	8487	
uCE6-1359	13733697	68485	
uCE6-1364	13794491	60794	
snp_T21H8[3]	13899897	105406	
uCE6-1370	14116294	216397	
uCE6-1371	14204533	88239	
uCE6-1387	14252287	47754	
uCE6-1392	14296325	44038	
snp_C11H1[3]	14324147	27822	
uCE6-1408	14375304	51157	
snp_Y60A9[2]	14380620	5316	
uCE6-1416	14415957	35337	
**snp_F23D12[2]**	14429469	13512	**ZHX-21a**
snp_F23D12[3]	14429615	146	
uCE6-1423	14431013	1398	
uCE6-1426	14431054	41	
uCE6-1430	14481319	50265	
uCE6-1437	14492472	11153	
uCE6-1438	14495144	2672	
snp_D1025[1]	14519808	24664	
pkP966	14532256	12448	
uCE6-1458	14545333	13077	
uCE6-1459	14547381	2048	
snp_C44H4[1]	14591890	44509	
snp_C44H4[6]	14598582	6692	
uCE6-1472	14602756	4174	
uCE6-1475	14670858	68102	
uCE6-1483	14693291	22433	
uCE6-1484	14705908	12617	
snp_F33C8[2]	14729282	23374	
pkP620	14761843	32561	
pkP5154	14854750	92907	
uCE6-1502	14878726	23976	
snp_F31F6[4]	14888320	9594	
uCE6-1513	15023336	135016	
uCE6-1515	15069299	45963	
snp_F09B12[5]	15092585	23286	
uCE6-1517	15121470	28885	
uCE6-1521	15139437	17967	
uCE6-1522	15139753	316	
snp_C11G10[7]	15153089	13336	
uCE6-1536	15222043	68954	
snp_F46F2[5]	15276110	54067	
snp_C02D4[2]	15284491	8381	
uCE6-1547	15312088	27597	

snp_Y15E3[2]	15316378	4290	
snp_Y15E3[3]	15316539	161	
snp_Y15E3[5]	15337551	21012	
snp_Y15E3[6]	15337553	2	
pkP6087	15357229	19676	
pkP914	15380699	23470	
snp_F47C8[1]	15385072	4373	
pkP5149	15390807	5735	
snp_F39D8[1]	15417248	26441	
uCE6-1551	15449663	32415	
**snp_C02C6[1]**	15560852	111189	**ZHX-06**
snp_C02C6[4]	15560864	12	
snp_C02C6[8]	15562668	1804	
snp_K09A9[2]	15590921	28253	
snp_K09E9[4]	15625902	34981	
snp_K09E9[5]	15629726	3824	
uCE6-1578	15633341	3615	
uCE6-1596	15897102	263761	
uCE6-1603	15963827	66725	
pkP573	16057991	94164	
snp_T27A8[1]	16060614	2623	
snp_T27A8[2]	16069162	8548	
uCE6-1610	16186374	117212	
uCE6-1618	16369261	182887	
snp_T24D11[2]	16408827	39566	
uCE6-1619	16416821	7994	
**snp_F38E9[2]**	16436347	19526	**ZHX-22**
snp_F59C12[1]	16602330	165983	
snp_C06G1[3]	16643059	40729	
uCE6-1630	16644814	1755	
snp_C06G1[4]	16646530	1716	
pkP521	16720217	73687	
**snp_K02H8[1]**	16991892	271675	**ZHX-23**
pkP5302	17051438	59546	
uCE6-1643	17228976	177538	
pkP651	17301912	72936	
snp_C53C11[3]	17315136	13224	
pkP5192	17386816	71680	
snp_R106[1]	17486068	99252	
uCE6-1653	17523703	37635	
pkP5022	17673951	150248	
pkP934	17673954	3	
pkP5071	17694254	20300	
pkP933	17694256	2	
