# Supplementary material for: A universal method for automated gene mapping
Source: Genome Biol. 2005 Jan 17;6(2):R19. doi: 10.1186/gb-2005-6-2-r19 (PMC551539; doi:10.1186/gb-2005-6-2-r19)
Supplement: Additional data file 18 — Additional non-validated FLPs (Drosophila) [file gb-2005-6-2-r19-s18.pdf]

Supplementary Table 10:

Additional *Drosophila* InDels and their physical position in the reference genome

(Validated FLP assays are shown in red)

Assay	Physical Position	Size Difference EP versus FRT	Remarks
2L008	2L:610032	1 bp	
2L017	2L:1676015	2 bp	
2L018	2L:1874668	1 bp	
2L027	2L:2855742..2855743	2 bp	
2L029.1	2L:3085920	1 bp	
2L029.5	2L:3086274	1 bp	
2L030	2R:3384440..3384459	20 bp	
2L033	2L:3827409..3827410	2 bp	
2L034.6	2L:3974902	1 bp	
2L034.12	2L:3974907	1 bp	
2L035	2L:4136161	7 bp	confirmed "PLP"-Assay available
2L038	2L:4368826..4368827	2 bp	
2L045.3	2L:5073745	1 bp	
2L045.5	2L:5073749	2 bp	
2L045.11	2L:5073821	2 bp	
2L045.18	2L:5074119	2 bp	
2L051	2L:5565388..5565394	7 bp	
2L057	2L:5950081	19 bp	
2L059.1	2L:6230674	12 bp	confirmed "PLP"-Assay available
2L059.2	2L:6230686	2 bp	
2L059.7	2L:6231150..6231165	16 bp	confirmed "PLP"-Assay available
2L059.11	2L:6231329	1 bp	
2L059.12	2L:6231338	2 bp	
2L059.16	2L:6231342	2 bp	
2L060	2L:6370531..6370534	4 bp	
2L063.2	2L:6799638..6799639	2 bp	
2L063.18	2L:68002660..68002670	11 bp	confirmed "PLP"-Assay available
2L069	2L:7729596	10 bp	
2L072.2	2L:8032241	2 bp	
2L072.23	2L:8032695	5 bp	
2L075	2L:8566907..8566915	5 bp	
2L077.8	2L:8942750	1 bp	
2L077.10	2L:8942850	2 bp	
2L079	2L:9205252	1 bp	
2L081.21	2L:9338427	1 bp	
2L081.23	2L:9338437	12 bp	confirmed "PLP"-Assay available
2L082.10	2L:7264579	4 bp	
2L082.11	2L:7264580	1 bp	
2L083	2L:9465292	2 bp	
2L084.2	2L:9595500..9595668	169 bp	confirmed "PLP"-Assay available
2L084.12	2L:9595750	1 bp	
2L084.20	2L:9595772	1 bp	
2L085.3	2L:9626161	4 bp	
2L085.4	2L:9626162	1 bp	
2L085.5	2L:9626163	1 bp	
2L085.7	2L:9626174	5 bp	
2L085.12	2L:9626251	1 bp	
2L086.3	2L:9716185..9716186	2 bp	
2L086.4	2L:9716194	1 bp	
2L086.5	2L:9716261	10 bp	confirmed "PLP"-Assay available
2L086.14	2L:9716868	4 bp	
2L088	2L:9835432	5 bp	
2L089	2L:99424412	1 bp	
2L090	2L:10076997	1 bp	
2L093	2L:10435401..10435415	16 bp	
2L095.9	2L:10772536	4 bp	
2L095.21	2L:10773334	1 bp	
2L097	2L:10949445	1 bp	
2L098	2L:11018242	6 bp	
2L104.6	2L:11491287	1 bp	
2L104.15	2L:11491789	1 bp	
2L112.50	2L:12107858	1 bp	
2L112.59	2L:12108346..12108350	5 bp	
2L113	2L:12232795	1 bp	
2L115	2L:12453638	1 bp	
2L117.4	2L:12600329	1 bp	
2L117.5	2L:12600330	1 bp	
2L117.7	2L:12600430	1 bp	
2L118	2L:12763814..12763815,	2 bp	
2L119	2L:12921658	11 bp	
2L120	2L:13092911	113 bp	confirmed "PLP"-Assay available
2L123.3	2L:13655090..13655093	4 bp	
2L123.4	2L:13655095..13655097	3 bp	
2L123.8	2L:13655130	1 bp	
2L123.19	2L:13655247	6 bp	
2L123.22	2L:13655254	4 bp	
2L123.25	2L:13655257	1 bp	
2L123.26	2L:13655259	4 bp	
2L123.27	2L:13655263	1 bp	
2L123.29	2L:13655824	2 bp	
2L124.3	2L:13707262	11 bp	
2L124.4	2L:13707284	1 bp	
2L124.5	2L:13707292	1 bp	
2L124.7	2L:13707301	1 bp	
2L124.8	2L:13707303	1 bp	
2L124.10	2L:13707331	6 bp	
2L125.6	2L:13820300..13820314	15 bp	confirmed "PLP"-Assay available
2L125.7	2L:13820406..13820407	2 bp	
2L125.14	2L:13820746.13820747	2 bp	
2L130.4	2L:14463995	1 bp	
2L130.7	2L:14464316	1 bp	
2L133	2L:15078246	1 bp	
2L136	2L:15285680	1 bp	
2L137	2L:15390931	1 bp	
2L139.5	2L:15687662..1568766	5 bp	
2L139.9	2L:15687816..15687817	2 bp	
2L139.22	2L:15688080	2 bp	
2L142.4	2L:16176802	1 bp	
2L142.7	2L:16176931	3 bp	

2L142.8	2L:16176933	1 bp	
2L142.9	2L:16176934	1 bp	
2L142.10	2L:16176941	1 bp	
2L142.12	2L:16176948	1 bp	
2L142.20	2L:16177068	3 bp	
2L142.26	2L:16177083	1 bp	
2L142.29	2L:16177090	1 bp	
2L142.33	2L:16177106	2 bp	
2L142.35	2L:16177108	3 bp	
2L142.37	2L:16177186	1 bp	
2L142.38	2L:16177188	1 bp	
2L143	2L:16311124..16311141	18 bp	
2L145.4	2L:16569653..16569657	5 bp	
2L145.16	2L:16569748	1 bp	
2L145.17	2L:16569750	1 bp	
2L145.18	2L:16569752	3 bp	
2L145.21	2L:16569763	1 bp	
2L145.22	2L:16569765	6 bp	
2L145.30	2L:16569848	1 bp	
2L145.31	2L:16569850	1 bp	
2L145.32	2L:16569852	1 bp	
2L145.43	2L:16570205	1 bp	
2L147.1	2L:16697027..16697038	12 bp	confirmed "PLP"-Assay available
2L147.2	2L:16697107..16697108	2 bp	
2L147.3	2L:16697116..16697140	25 bp	confirmed "PLP"-Assay available
2L149	2L:16826201..16826207	7 bp	
2L150	2L:16954176..16954177	2 bp	
2L167	2L:18328150	1 bp	
2L169	2L:18450599	1 bp	
2L175	2L:18962692	1 bp	
2L185.1	2L:19553279..19553285	7 bp	confirmed "PLP"-Assay available
2L185.2	2L:19553374..19553379	6 bp	
2L186.2	2L:19573326	1 bp	
2L186.7	2L:19573386	1 bp	
2L187.5	2L:19666829	1 bp	
2L187.7	2L:19666901..19666904	4 bp	
2L187.14	2L:19667379	1 bp	
2L189.1	2L:20016919	8 bp	confirmed "PLP"-Assay available
2L189.4	2L:20017732	1 bp	
2L198	2L:21077724	1 bp	
2L199	2L:21209718	3 bp	
2R006	2R:3099968	1 bp	
2R008	2R:3393756	12 bp	confirmed "PLP"-Assay available
2R011	2R:3668022	1 bp	
2R013	2R:4018842	1 bp	
2R017	2R:4570108..4570123	10 bp	
2R020	2R:4997880	1 bp	
2R021	2R:5138120	1 bp	
2R023	2R:5402665	1 bp	
2R027.2	2R:5679231	2 bp	
2R027.4	2R:5679376	2 bp	
2R028	2R:5781425	4 bp	
2R029	2R:5929226	2 bp	
2R032.11	2R:6438030	1 bp	
2R032.12	2R:6438045	1 bp	
2R032.15	2R:6438055	2 bp	
2R033.22	2R:6546302..6546305	4 bp	
2R033.24	2R:6546312..6546314	3 bp	
2R034	2R:6659531	1 bp	
2R036.5	2R:6899192..6899194	3 bp	
2R036.6	2R:6899196..6899197	2 bp	
2R036.7	2R:6899199..6899201	3 bp	
2R038.3	2R:7147742	1 bp	
2R038.4	2R:7147745..7147746	2 bp	
2R039	2R:7269311..7269326	16 bp	
2R041.1	2R:7345919..7345921	3 bp	
2R041.5	2R:7346093..7346095	3 bp	
2R042.4	2R:7446339	2 bp	
2R042.11	2R:7446543..7446551	9 bp	
2R042.15	2R:7446936	1 bp	
2R044.3	2R:7544901..7544903	3 bp	
2R044.5	2R:7545110..7545111	2 bp	
2R046	2R:7662361..7662370	10 bp	confirmed "PLP"-Assay available
2R049	2R:7840372	1 bp	
2R051	2R:8076818	2 bp	
2R054.1	2R:8525353	1 bp	
2R054.26	2R:8526187	5 bp	
2R054.27	2R:8526205	1 bp	
2R056	2R:8857453..8857454	2 bp	
2R057	2R:8963680..8963682	3 bp	
2R058	2R:8994637	1 bp	
2R060	2R:9182288..9182293	6 bp	
2R061	2R:9287474	1 bp	
2R064	2R:9687965..9687966	2 bp	
2R067	2R:10022333	1 bp	
2R068	2R:10140014..10140021	8 bp	
2R072	2R:10528095	1 bp	
2R073	2R:10618248	1 bp	
2R075	2R:10856817	1 bp	
2R076.4	2R:10974423	8 bp	
2R076.7	2R:10974699	1 bp	
2R076.10	2R:10974758..10974759	2 bp	
2R076.23	2R:10975177	1 bp	
2R081.5	2R:11530312	1 bp	
2R081.6	2R:11530326	1 bp	
2R081.8	2R:11530339..11530342	4 bp	
2R083	2R:11749438..11749448	11 bp	
2R087.3	2R:12093105	2 bp	
2R087.4	2R:12093124	6 bp	
2R087.5	2R:12093348	4 bp	
2R088.1	2R:12179149	2 bp	
2R088.2	2R:12179157	1 bp	
2R088.6	2R:12179766..12179769	4 bp	
2R088.7	2R:12179786	1 bp	
2R090	2R:12466118	1 bp	
2R094.20	2R:12919255	4 bp	
2R094.22	2R:12919320..12919327	8 bp	confirmed "PLP"-Assay available

2R096	2R:13256954	29 bp	
2R097.9	2R:13372294..13372296	3 bp	confirmed "PLP"-Assay available
2R097.13	2R:13372555	5 bp	confirmed "PLP"-Assay available
2R097.14	2R:13372556	3 bp	
2R097.15	2R:13372559	3 bp	
2R101.4	2R:13992673..13992677	3 bp	
2R101.12	2R:13992904	1 bp	
2R101.13	2R:13992905	1 bp	
2R104	2R:14436207	1 bp	
2R107.4	2R:14933268..14933280	13 bp	
2R107.12	2R:14933482..14933485	4 bp	
2R109	2R:15200071..15200083	11 bp	
2R111.5	2R:15313752	1 bp	
2R111.18	2R:15314122..15314136	14 bp	confirmed "PLP"-Assay available
2R111.23	2R:15314419	1 bp	
2R114	2R:15541095..15541105	11 bp	
2R116.3	2R:15823034	1 bp	
2R116.7	2R:15823109	3 bp	
2R117.2	2R:15840311..15840318	8 bp	confirmed "PLP"-Assay available
2R117.11	2R:15840906	1 bp	
2R117.12	2R:15840976	2 bp	
2R118	2R:16117998	2 bp	
2R119.12	2R:16154421	2 bp	
2R119.15	2R:16154530	1 bp	
2R120.1	2R:16261681	4 bp	confirmed "PLP"-Assay available
2R120.2	2R:16261689	8 bp	
2R120.8	2R:16262221	9 bp	
2R124	2R:16841016	14 bp	
2R125.2	2R:16985241	1 bp	
2R125.12	2R:16985523	1 bp	
2R128	2R:17275552	1 bp	
2R130	2R.17944415	9 bp	
2R134.3	2R:18395540..18395572	33 bp	confirmed "PLP"-Assay available
2R134.5	2R:18395602	1 bp	
2R134.10	2R:18395949	1 bp	
2R139	2R:18613351	18 bp	
2R140	2R:18627214..18627219	6 bp	
2R141.4	2R:18641638	5 bp	
2R141.5	2R:18641698..18641700	3 bp	
2R141.8	2R:18641921	3 bp	
2R143	2R:18979211	5 bp	
2R145	2R:19189216	1 bp	
2R146	2R:19309208..19309211	4 bp	
2R147	2R:19435767	4 bp	
2R150	2R:19891477	3 bp	
3L009	3L:1862477..1862480	5 bp	
3L013	3L:8817743	1 bp	
3L015.7	3L:2448419	2 bp	
3L015.9	3L:2448481	1 bp	
3L015.12	3L:2448664	3 bp	
3L015.13	3L:2448666	6 bp	
3L021	3L:2808041	12 bp	
3L022	3L:2862110..2862121	11 bp	confirmed "PLP"-Assay available
3L023	3L:2907068	1 bp	
3L025.1	3L:3012192	1 bp	
3L025.10	3L:3012361	4 bp	
3L025.14	3L:3012384	2 bp	
3L025.15	3L:3012714	1 bp	
3L026	3L:3090357	13 bp	confirmed "PLP"-Assay available
3L030	3L:3542647	3 bp	
3L031	3L:3757025	19 bp	
3L033	3L:3973466	1 bp	
3L034.2	3L:4202140	1 bp	
3L034.3	3L:4202471	1 bp	
3L035.2	3L:4319550	1 bp	
3L035.4	3L:4319563	4 bp	
3L035.6	3L:4319929..4319934	6 bp	
3L035.8	3L:4319957	1 bp	
3L035.13	3L:4320346..53	8 bp	
3L035.19	3L:4320412..4320418	7 bp	
3L035.21	3L:4320544	17 bp	
3L035.24	3L:4320572	1 bp	
3L036	3L:4420492..4420497	6 bp	
3L037.2	3L:4616760 ..4616765	6 bp	
3L037.3	3L:4616772 ..4616815	45 bp	confirmed "PLP"-Assay available
3L037.7	3L:4616995	1 bp	
3L037.8	3L:4617130	25 bp	confirmed "PLP"-Assay available
3L037.16	3L:4617197	11 bp	
3L039	3L:3889000	1 bp	
3L041	3L:5182778..5182806	28 bp	
3L043	3L:5363727	4 bp	
3L045	3L:5540352	1 bp	
3L048.5	3L:5853416	3 bp	
3L048.11	3L:5853425	3 bp	
3L048.14	3L:5853481	4 bp	
3L048.18	3L:5853521	1 bp	
3L048.27	3L:5854035	2 bp	
3L048.28	3L:5854048	2 bp	
3L052	3L:6079860..6079861	2 bp	
3L056	3L:6392307	1 bp	
3L057	3L:6472326	1 bp	
3L058	3L:6563733	11 bp	
3L059.4	3L:6662154..6662156	3 bp	confirmed "PLP"-Assay available
3L059.5	3L:6662158	1 bp	
3L059.6	3L:6662161	1 bp	
3L059.7	3L:6662164..6662166	3 bp	
3L060.9	3L:6745384	1 bp	
3L060.14	3L:6745396	2 bp	
3L063.4	3L:6928344	1 bp	
3L063.9	3L:6928446	1 bp	
3L064	3L:7023027..7023047	21 bp	
3L066.9	3L:7390780..7390784	5 bp	
3L066.10	3L:7390786	1 bp	
3L070.2	3L:7765049	3 bp	
3L070.3	3L:7765066..7765073	8 bp	
3L072.15	3L:7869508	1 bp	
3L072.16	3L:7869517	1 bp	
3L072.18	3L:7869519	1 bp	

3L073.1	3L:7948541	12 bp	confirmed "PLP"-Assay available
3L073.10	3L:7949231	1 bp	
3L076	3L:8220866..8220869	4 bp	
3L077	3L:8382141	26 bp	confirmed "PLP"-Assay available
3L079	3L:8663494	1 bp	
3L083	3L:9257026	5 bp	
3L084	3L:9396606	2 bp	
3L086	3L:9701769	15 bp	
3L088	3L:10005193	1 bp	
3L089	3L:10158269	1 bp	
3L090	3L:10271526	1 bp	
3L093.1	3L:10500538	1 bp	
3L093.4	3L:10500551	2 bp	
3L093.36	3L:10501189	1 bp	
3L093.38	3L:10501260	3 bp	
3L094	3L:10614419..10614428	10 bp	
3L095.2	3L:10766245	1 bp	
3L095.7	3L:10766484	1 bp	
3L095.17	3L:10766657	4 bp	
3L098.2	3L:11375746..11375749	4 bp	
3L098.11	3L:11375912	6 bp	
3L098.13	3L:11375917	1 bp	
3L098.16	3L:11375964..11375980	17 bp	
3L098.17	3L:11375986..11376070	85 bp	confirmed "PLP"-Assay available
3L098.19	3L:11376092	2 bp	
3L098.23	3L:11376217	1 bp	
3L098.25	3L:11376220	2 bp	
3L098.26	3L:11376275	2 bp	
3L098.41	3L:11376622..11376642	21 bp	confirmed "PLP"-Assay available
3L098.45	3L:11376702..11376709	8 bp	
3L098.47	3L:11376715..11376722	8 bp	
3L098.48	3L:11376728..11376730	3 bp	
3L104.5	3L:12050159..12050160	2 bp	
3L104.6	3L:12050186	1 bp	
3L104.9	3L:12050194	1 bp	
3L105	3L:12442330..124423346	17 bp	
3L113.4	3L:13122727..13122738	12 bp	confirmed "PLP"-Assay available
3L113.8	3L:13122959	1 bp	
3L115.9	3L:13350356	1 bp	
3L115.11	3L:13350388..13350416	29 bp	confirmed "PLP"-Assay available
3L115.17	3L:13350490	10 bp	
3L115.18	3L:13350495	21 bp	
3L115.25	3L:13350552	1 bp	
3L115.33	3L:13350576	1 bp	
3L115.35	3L:13350612	1 bp	
3L115.36	3L:13350616	3 bp	
3L115.38	3L:13350622	2 bp	
3L115.39	3L:13350624	1 bp	
3L116	3L:13477759	13 bp	confirmed "PLP"-Assay available
3L118.7	3L:13738397	1 bp	
3L118.11	3L:13738711	1 bp	
3L118.13	3L:13738743	1 bp	
3L119.25	3L:13885697	1 bp	
3L119.26	3L:13885705	1 bp	
3L120.7	3L:14009133..14009134	2 bp	
3L120.9	3L:14009138..14009143	6 bp	
3L120.12	3L:14009251	1 bp	
3L120.13	3L:14009281	1 bp	
3L120.14	3L:14009291..14009292	2 bp	
3L120.15	3L:14009313..14009323	11 bp	confirmed "PLP"-Assay available
3L121.6	3L:14291187	1 bp	
3L121.7	3L:14291377..14291381	5 bp	
3L124	3L:14690818..14690822	5 bp	
3L125.5	3L:14851835..14851836	2 bp	
3L125.7	3L:14851949..14851965	17 bp	confirmed "PLP"-Assay available
3L126.2	3L:15134444	1 bp	
3L126.9	3L:15134451	4 bp	
3L126.10	3L:15134452	3 bp	
3L126.11	3L:15134455	9 bp	
3L127	3L:15416175..15416186	12 bp	
3L128.1	3L:15556606	2 bp	
3L128.3	3L:15556878	1 bp	
3L130.2	3L:15815682	1 bp	
3L130.4	3L:15815766	1 bp	
3L130.5	3L:15815805	6 bp	
3L135.2	3L:16357228	1 bp	
3L135.9	3L:16358179	6 bp	
3L137.2	3L:16605243	2 bp	
3L137.3	3L:16605249	4 bp	
3L137.4	3L:16605255	2 bp	
3L137.6	3L:16605453	1 bp	
3L140	3L:16721765	1 bp	
3L148	3L:17527516..17527520	5 bp	
3L158	3L:18316764	1 bp	
3L159	3L:18497857	1 bp	
3L163.9	3L:18871643	1 bp	
3L163.15	3L:18871947	2 bp	
3L163.19	3L:18871978	1 bp	
3L164	3L:1905868	1 bp	
3L165	3L:19151190	3 bp	
3L166	3L:19241268	2 bp	
3L167	3L:19244958	1 bp	
3L169.1	3L:19432263	2 bp	
3L169.3	3L:19432636	2 bp	
3L170.5	3L:19625255..19625259	5 bp	
3L170.6	3L:19625262	1 bp	
3L170.12	3L:19625450	3 bp	
3L171	3L:19826748..19826764	17 bp	confirmed "PLP"-Assay available
3L173	3L:20322167..20322206	40 bp	confirmed "PLP"-Assay available
3L177.3	3L:20902735..20902737	3 bp	
3L177.4	3L:20902739	1 bp	
3L180	3L:21476236	1 bp	
3L181	3L:21656546	3 bp	
3L183.1	3L:22030780	1 bp	
3L183.7	3L:22031238	1 bp	
3L184	3L:22226259..22226298	40 bp	
3L185	3L:22583786..22583790	5 bp	confirmed "PLP"-Assay available

3L186	3L:22775660	11 bp	confirmed "PLP"-Assay available
3R010	3R:1552157..1552165	9 bp	
3R011	3R:1599768	9 bp	confirmed "PLP"-Assay available
3R012.3	3R:1761135..1761156	22 bp	confirmed "PLP"-Assay available
3R012.4	3R:1761237..1761245	9 bp	
3R013.1	3R:1923170..1923177	8 bp	
3R013.5	3R:1923717	1 bp	
3R013.6	3R:1923718	1 bp	
3R013.7	3R:1923720	4 bp	
3R015	3R:2162943..2163000	58 bp	confirmed "PLP"-Assay available
3R019	3R:2800696	1 bp	
3R020.1	3R:2961042	1 bp	
3R020.2	3R:2961246	1 bp	
3R023.4	3R:3410348	2 bp	
3R023.6	3R:3410413	1 bp	
3R026.3	3R:3780912	1 bp	
3R026.5	3R:3781147	4 bp	
3R029	3R:4181507	3 bp	
3R032	3R:4672533..4672547	15 bp	confirmed "PLP"-Assay available
3R034	3R:4985172..4985173	2 bp	
3R035	3R:5304873..5304880	8 bp	
3R038	3R:5716628..5716629	2 bp	
3R039	3R:5796772..5796779	8 bp	confirmed "PLP"-Assay available
3R043.6	3R:6279153..6279157	5 bp	
3R043.9	3R:6279165	6 bp	
3R045.17	3R:6507996..6508001	6 bp	
3R045.22	3R:6508585	6 bp	
3R046.5	3R:6559253	1 bp	
3R046.7	3R:6559254	2 bp	
3R046.11	3R:6559447	1 bp	
3R047	3R:6759078	1 bp	
3R048	3R:6909315	1 bp	
3R050	3R:7082702	2 bp	
3R052.6	3R:7314541	1 bp	
3R052.7	3R:7314548	7 bp	
3R053.15	3R:7381669	1 bp	
3R053.16	3R:7381836	1 bp	
3R057	3R:8054196	1 bp	
3R061	3R:8555144..8555151	8 bp	
3R062.1	3R:8716124	1 bp	
3R062.2	3R:8716134	1 bp	
3R062.5	3R:8716328	1 bp	
3R062.9	3R:8716465	3 bp	
3R062.10	3R:8716563	1 bp	
3R064	3R:9036569	1 bp	
3R065	3R:9181888..9181919	32 bp	confirmed "PLP"-Assay available
3R066.4	3R:9355810	11 bp	confirmed "PLP"-Assay available
3R066.6	3R:9356315	1 bp	
3R066.7	3R:9356317	1 bp	
3R068.1	3R:9675570	1 bp	
3R068.2	3R:9675572	1 bp	
3R068.3	3R:9675575	4 bp	
3R068.9	3R:9675918	2 bp	
3R070	3R:10118729	1 bp	
3R071	3R:10158219	4 bp	
3R072	3R:10316017	1 bp	
3R074	3R:1063037	1 bp	
3R075	3R:10795309	1 bp	
3R082.1	3R:11589689	1 bp	
3R082.3	3R:11590076	6 bp	
3R083.2	3R:11753633	6 bp	confirmed "PLP"-Assay available
3R083.3	3R:11753634	1 bp	
3R083.4	3R:11753636	3 bp	
3R083.5	3R:11753637	4 bp	
3R083.8	3R:11754132	10 bp	confirmed "PLP"-Assay available
3R085	3R:12074352..12074364	13 bp	confirmed "PLP"-Assay available
3R086	3R:12234229..12234237	9 bp	confirmed "PLP"-Assay available
3R090	3R:12714059	1 bp	
3R091.2	3R:12873878	2 bp	
3R091.4	3R:12873960	1 bp	
3R091.13	3R:12874178	3 bp	
3R091.27	3R:12874396..12874403	8 bp	
3R092	3R:13035467	35 bp	
3R096	3R:13396517	1 bp	
3R105	3R:13770858	1 bp	
3R108	3R:13914979	1 bp	
3R109	3R:13963273	1 bp	
3R118	3R:14313562	1 bp	
3R122	3R:14763119..14763130	12 bp	
3R123.2	3R:14793328	10 bp	
3R123.4	3R:14793415	13 bp	confirmed "PLP"-Assay available
3R123.17	3R:14793878..14793889	12 bp	
3R125	3R:14940814	2 bp	
3R127.1	3R:14988403..14988411	9 bp	
3R127.13	3R:14988677	1 bp	
3R127.22	3R:14988787	8 bp	
3R127.26	3R:14988823	1 bp	
3R127.31	3R:14988977	1 bp	
3R151	3R:16242467	16 bp	
3R152	3R:16279648..16279649	2 bp	
3R156.5	3R:16878348	1 bp	
3R156.7	3R:16878425	1 bp	
3R156.9	3R:16878543	1 bp	
3R156.11	3R:16878552	1 bp	
3R156.14	3R:16878660..16878677	18 bp	confirmed "PLP"-Assay available
3R160	3R:17363059	1 bp	
3R162	3R:17618247	4 bp	
3R169	3R:18484180	6 bp	
3R170	3R:18486183	1 bp	
3R171	3R:18571156	2 bp	
3R174	3R:18763223	1 bp	
3R176	3R:19043017	1 bp	
3R186	3R:19738651	3 bp	
3R187.5	3R:19880268..19880270	3 bp	
3R187.11	3R:19880833..19880835	3 bp	
3R192	3R:20557003	5 bp	
3R200	3R:21622103	1 bp	
3R204	3R:22075492	10 bp	
3R205	3R:22168325	1 bp	

3R212	3R:23452172	1 bp	
3R217.1	3R:24093857..24093858	2 bp	
3R217.2	3R:24093864..24093866	3 bp	
3R218	3R:24255318..24255321	4 bp	
3R221	3R:24908015..24908060	46 bp	
3R222.20	3R:25034769	2 bp	
3R222.22	3R:25034973..25034989	17 bp	confirmed "PLP"-Assay available
3R224	3R:25285450..25285463	10 bp	
3R227.10	3R:25534538	2 bp	
3R227.11	3R:25534539	4 bp	
3R227.14	3R:25534611..25534624	14 bp	confirmed "PLP"-Assay available
3R228.6	3R:25573620..25573621	2 bp	
3R228.9	3R:25573624..25573625	2 bp	
3R230	3R:25765333..25765341	9 bp	
3R231.1	3R:25875660	18 bp	confirmed "PLP"-Assay available
3R231.3	3R:25876079	3 bp	
3R231.6	3R:25876291	3 bp	
3R232	3R:25957905	11 bp	confirmed "PLP"-Assay available
3R233	3R:25997115	50 bp	confirmed "PLP"-Assay available
3R236.8	3R:26189922	1 bp	
3R236.9	3R:26189941	14 bp	confirmed "PLP"-Assay available
3R238.5	3R:26515872	2 bp	
3R238.6	3R:26515881	1 bp	
3R238.21	3R:26516008..26516077	70 bp	confirmed "PLP"-Assay available
3R238.23	3R:26516134	9 bp	confirmed "PLP"-Assay available
3R244.2	3R:27155235	1 bp	
3R244.3	3R:27155237	2 bp	
3R244.4	3R:27155251..27155259	9 bp	
3R245	3R:27189945..27189950	6 bp	
3R246	3R:27316459	1 bp	
3R248.3	3R:27635556	4 bp	
3R248.4	3R:27635558	4 bp	
3R248.5	3R:27635559	8 bp	
3R249	3R:27766585..27766603	19 bp	confirmed "PLP"-Assay available
